# Supplementary material for: Discovery of oncogenic ROS1 missense mutations with sensitivity to tyrosine kinase inhibitors
Source: EMBO Mol Med. 2023 Aug 17;15(10):e17367. doi: 10.15252/emmm.202217367 (PMC10565643; doi:10.15252/emmm.202217367)
Supplement: Supplementary file 1 — Expanded View Figures PDF [file EMMM-15-e17367-s011.pdf]

Expanded View Figures

A

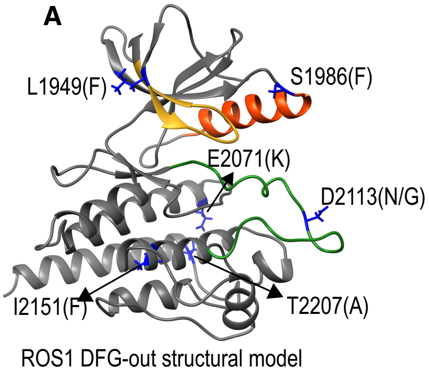

B

Mutation	Region of Kinase Domain	Tumor Type	Frequency of Somatic Mutations
L1949F	P-Loop	Melanoma	1/5402 (0.02%)
S1986F	αC-Helix	Glioma	1/9061 (0.01%)
		Melanoma	1/5402 (0.02%)
		Ovarian Cancer	1/5428 (0.02%)
E2071K	αE-Helix	Breast Cancer	1/856 (0.12%)
		Glioma	1/9061 (0.01%)
		Melanoma	5/5402 (0.06%)
D2113G	A-Loop	Esophageal Adenocarcinoma	1/53 (1.9%)
D2113N	A-Loop	Glioma	1/9061 (0.01%)
		Melanoma	5/5402 (0.1%)

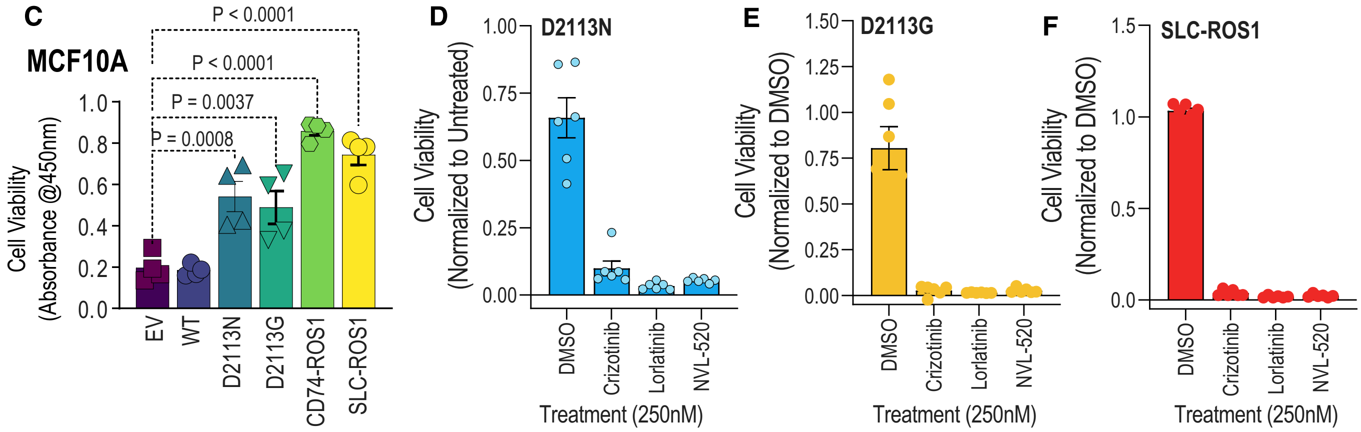

Figure EV1. EGF-independent growth of MCF10A ROS1 D2113N, D2113G or SLC34A2-ROS1 fusions is sensitivity to ROS1 TKI.

A Ribbon diagram of ROS1 kinase domain structural model annotated with amino acid substitutions that increased catalytic activity.

B Frequency of activating ROS1 alterations in cancer.

C Colorimetric cell viability data after growth of MCF10A cells expressing Empty Vector (EV), ROS1 wildtype (WT), $ROS1^{D2113N}$, $ROS1^{D2113G}$, CD74-ROS1 fusion and SLC34A2-ROS1 (SLC-ROS1) fusion in low EGF medium for 108 h (N = 4, biological replicates). CCK-8 (water-soluble tetrazolium salt, WST-8) reagent was added to the 96-well plates after completion of real-time imaging in the Incucyte® live imaging platform.

D–F Cell viability of MCF10A $ROS1^{D2113N}$ (D), D2113G (E) and SLC-ROS1 (F) in low EGF medium with or without ROS1 TKI, crizotinib, lorlatinib, and NVL-520 (250 nM in all cases) for 108 h, measured using CCK-8 colorimetric reagent (N = 6, biological replicates). One-way ANOVA with Dunnett's multiple comparisons test was used with alpha of 0.05. Error bars in figure represent mean ± SEM.

Source data are available online for this figure.

© 2023 The Authors

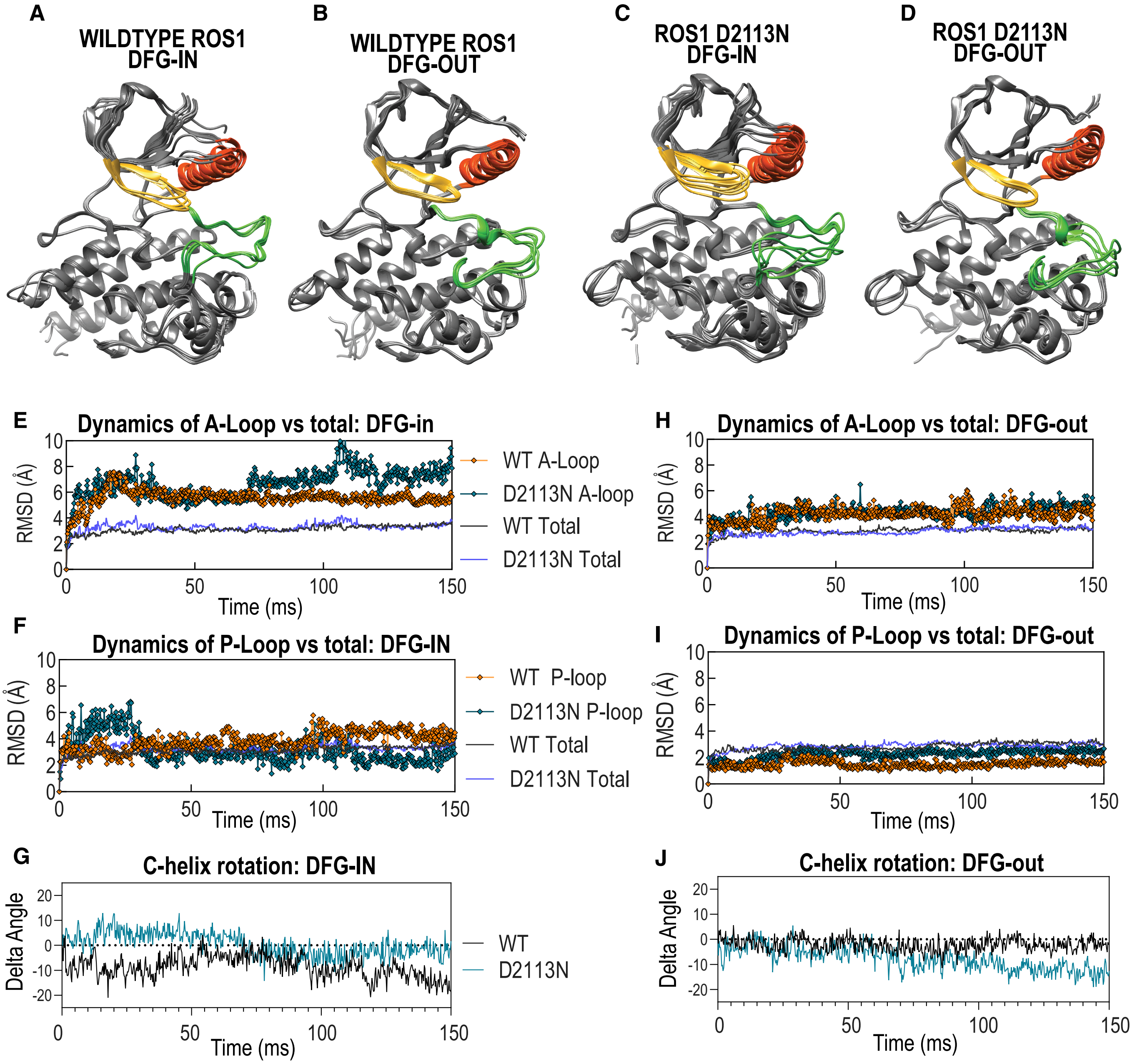

Figure EV2. Molecular dynamic simulations of ROS1^{WT} and ROS1^{D2113N} kinase demonstrate effects on A-loop in the DFG-in conformation but minimal impact on other domains.

A–J (A–D) Six individual representative poses adopted by ROS1^{WT} and ROS1^{D2113N} in DFG-in and DFG-out conformations as indicated by labels. Root Mean Square Deviation values (*Y*-axis) plotted over 150 ms of simulation duration (*X*-axis) for A-loop in DFG-in conformation (E), A-loop DFG-out conformation (H), P-loop in DFG-in (F) and DFG-out (I) conformations and αC-helix in DFG-in (G) and DFG-out (J) conformations.

 © 2023 The Authors

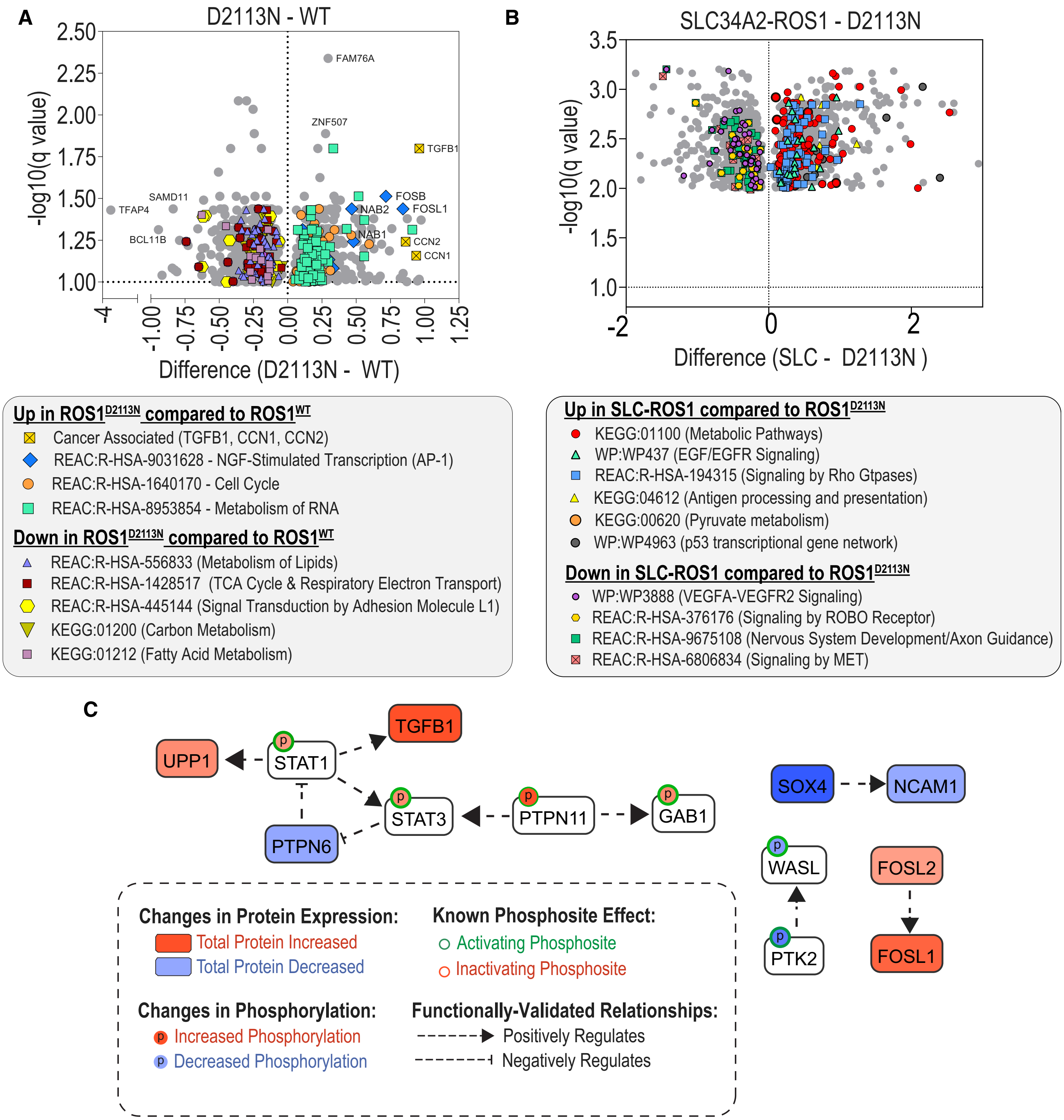
A
D2113N - WT
-log10(q value)
Difference (D2113N - WT)
FAM76A
ZNF507
TGFB1
SAMD11
TFAP4
BCL11B
FOSB
NAB2
FOSL1
NAB1
CCN2
CCN1
Up in ROS1D2113N compared to ROS1WT
Cancer Associated (TGFB1, CCN1, CCN2)
REAC:R-HSA-9031628 - NGF-Stimulated Transcription (AP-1)
REAC:R-HSA-1640170 - Cell Cycle
REAC:R-HSA-8953854 - Metabolism of RNA
Down in ROS1D2113N compared to ROS1WT
REAC:R-HSA-556833 (Metabolism of Lipids)
REAC:R-HSA-1428517 (TCA Cycle & Respiratory Electron Transport)
REAC:R-HSA-445144 (Signal Transduction by Adhesion Molecule L1)
KEGG:01200 (Carbon Metabolism)
KEGG:01212 (Fatty Acid Metabolism)
B
SLC34A2-ROS1 - D2113N
-log10(q value)
Difference (SLC - D2113N)
Up in SLC-ROS1 compared to ROS1D2113N
KEGG:01100 (Metabolic Pathways)
WP:WP437 (EGF/EGFR Signaling)
REAC:R-HSA-194315 (Signaling by Rho Gtpases)
KEGG:04612 (Antigen processing and presentation)
KEGG:00620 (Pyruvate metabolism)
WP:WP4963 (p53 transcriptional gene network)
Down in SLC-ROS1 compared to ROS1D2113N
WP:WP3888 (VEGFA-VEGFR2 Signaling)
REAC:R-HSA-376176 (Signaling by ROBO Receptor)
REAC:R-HSA-9675108 (Nervous System Development/Axon Guidance)
REAC:R-HSA-6806834 (Signaling by MET)
C
UPP1
STAT1
TGFB1
STAT3
PTPN6
PTPN11
GAB1
SOX4
NCAM1
WASL
PTK2
FOSL2
FOSL1
Changes in Protein Expression:
Total Protein Increased
Total Protein Decreased
Known Phosphosite Effect:
Activating Phosphosite
Inactivating Phosphosite
Changes in Phosphorylation:
Increased Phosphorylation
Decreased Phosphorylation
Functionally-Validated Relationships:
Positively Regulates
Negatively Regulates

Figure EV3.

◀ **Figure EV3. Global proteomics and causal path analysis for ROS1^{D2113N}.**

A Volcano plots showing differential expression of proteins in HEK293A ROS1^{D2113N} cells compared to HEK293A ROS1^{WT} cells (N = 3, biological replicates). Multiple unpaired *t*-test analysis was performed on $\log_2$ transformed ratio of normalized intensities (D2113N—WT). Multiple comparisons were via false discovery rate (FDR) with Desired FDR Q = 10%. Two-stage step-up Benjamini, Krieger, and Yekutieli method was used for the false discovery rate approach. The *X*-axis features significant discoveries of ROS1^{D2113N} relative to ROS1^{WT} (D2113N—WT) and *Y*-axis features corresponding log10(q) values (shown as –log10(q)). G:Profiler: a web server for functional enrichment analysis was used to curate significantly upregulated or downregulated pathways associated with KEGG, Reactome or Wikipathways databases (Raudvere *et al*, 2019) and indicated in annotated box below.

B Volcano plots showing differential expression of proteins in HEK293A SLC-ROS1 compared to HEK293A ROS1^{D2113N} cells (N = 3, biological replicates). All analysis parameters are identical to described above in panel (A) with the exception of the Desired FDR Q = 1% in the SLC-ROS1—D2113N analysis.

C Causalpath analysis of global proteomics and phosphoproteomics log2-transformed spectral counts highlights signaling changes promoted by ROS1^{D2113N} relative to ROS1^{WT}. Source data is located in Dataset EV2.

© 2023 The Authors

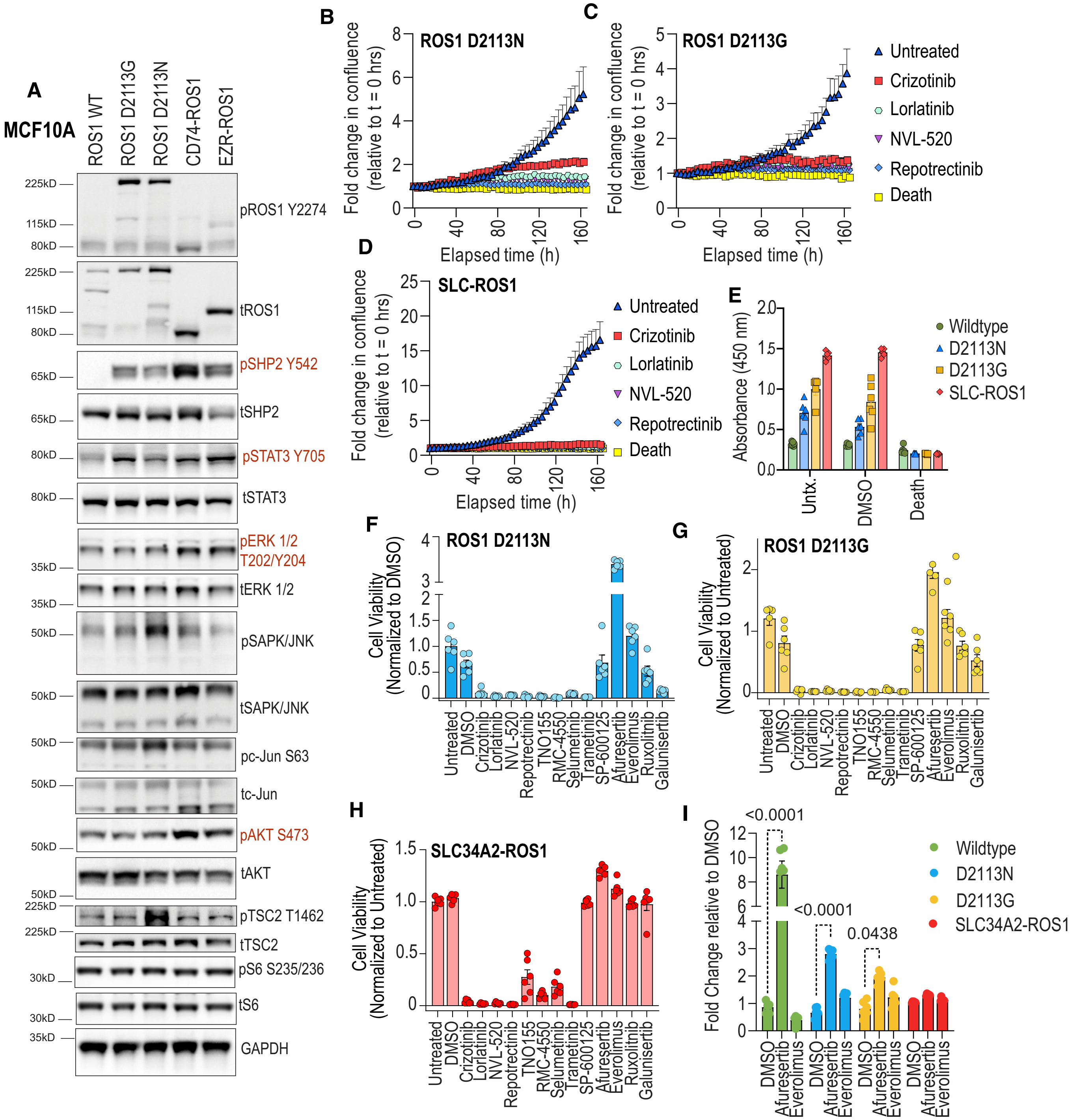
A
MCF10A
ROS1 WT
ROS1 D2113G
ROS1 D2113N
CD74-ROS1
EZR-ROS1
pROS1 Y2274
tROS1
pSHP2 Y542
tSHP2
pSTAT3 Y705
tSTAT3
pERK 1/2 T202/Y204
tERK 1/2
pSAPK/JNK
tSAPK/JNK
pc-Jun S63
tc-Jun
pAKT S473
tAKT
pTSC2 T1462
tTSC2
pS6 S235/236
tS6
GAPDH
B
ROS1 D2113N
C
ROS1 D2113G
Fold change in confluence (relative to t = 0 hrs)
Elapsed time (h)
Untreated
Crizotinib
Lorlatinib
NVL-520
Repotrectinib
Death
D
SLC-ROS1
E
Absorbance (450 nm)
Wildtype
D2113N
D2113G
SLC-ROS1
Untx.
DMSO
Death
F
ROS1 D2113N
Cell Viability (Normalized to DMSO)
G
ROS1 D2113G
Cell Viability (Normalized to Untreated)
Untreated
DMSO
Crizotinib
Lorlatinib
NVL-520
Repotrectinib
TNO155
RMC-4550
Selumetinib
Trametinib
SP-600125
Afuresertib
Everolimus
Ruxolitinib
Galunisertib
H
SLC34A2-ROS1
I
Fold Change relative to DMSO
<0.0001
<0.0001
0.0438
Wildtype
D2113N
D2113G
SLC34A2-ROS1

Figure EV4.

Figure EV4. Regulation of signaling pathways in MCF10A ROS1 variant expressing cells.

A Immunoblots performed on cell lysates generated from stable MCF10A cell lines expressing $ROS1^{WT}$, $ROS1^{D2113N}$, $ROS1^{D2113G}$, CD74-ROS1 and EZR-ROS1 fusion proteins. Phosphosite specific and total antibodies used are indicated in the blot labels. Following target proteins were interrogated: ROS1, SHP2, STAT3, ERK1/2, JNK/SAPK, c-Jun, AKT1, TSC2, S6, and GAPDH (Loading control). Red colored western blot labels indicate pathways that are upregulated either in ROS1 point mutations, ROS1 fusions or both. Prior to cell lysis, EGF withdrawal was done for 4 h.

B–D Inhibition of cell proliferation in ROS1 TKI treated MCF10A $ROS1^{D2113N}$ (B), $ROS1^{D2113G}$ (C) and SLC34A2-ROS1 (D) cell lines ($N = 6$, biological replicates). Cells were treated with 250 nM crizotinib, lorlatinib, NVL-520 and repotrectinib for the duration of the live-imaging experiment. “Death” indicates a cocktail of 10 μM staurosporine plus YM155 inhibitors that induce complete death and serve a positive control for cell death in live imaging and colorimetric cell viability assays.

E Colorimetric cell viability data from MCF10A $ROS1^{WT}$, $ROS1^{D2113N}$, $ROS1^{D2113G}$, and SLC34A2-ROS1 cell lines that were untreated, treated with DMSO, or “Death” cocktail as explained previously ($N = 6$, biological replicates).

F–H Colorimetric cell viability data from $ROS1^{D2113N}$ (F), $ROS1^{D2113G}$ (G), and SLC34A2-ROS1 (H) cell lines treated with the indicated signaling effector inhibitors ($N = 6$, biological replicates). 250 nM treatment with DMSO, crizotinib, lorlatinib, NVL-520, repotrectinib, TNO155, RMC-4550, selumetinib, trametinib, afuresertib, everolimus, SP-600125, and ruxolitinib. 500 nM treatment with galusertinib.

I Colorimetric cell viability data from MCF10A $ROS1^{WT}$, $ROS1^{D2113N}$, $ROS1^{D2113G}$, and SLC34A2-ROS1 treated with DMSO, 250 nM afuresertib, or 250 nM everolimus. *P* values from multiple comparison tests displayed in chart. Cell viability measurements displayed in figure were either raw absorbance of reduced tetrazolium salt, WST-8 (CCK-8), measured at 460 nm, or fold change of raw absorbance relative to DMSO control. Data in panels (E–I) was analyzed using two-way ANOVA with alpha of 0.05. Dunnett’s multiple comparison test was used. Statistical analyses are reported in Source Data. Error bars in figure represent mean ± SEM.

Source data are available online for this figure.

 © 2023 The Authors

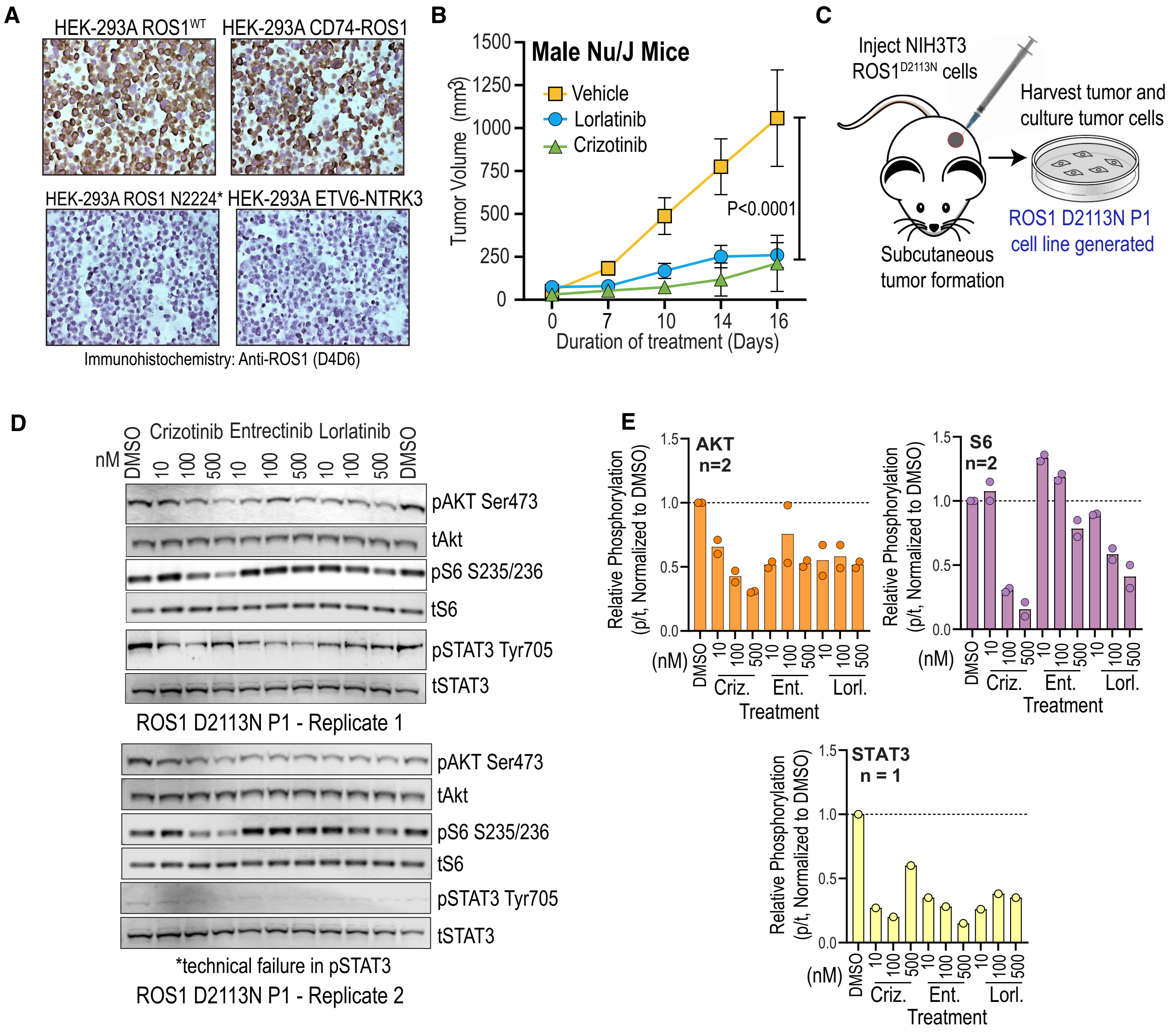

Figure EV5. ROS1^{D2113N} tumors and the tumor-derived cell line respond to ROS1-TKI treatment.

A Immunohistochemistry (ICC) of 20 μm sections from formalin-fixed, paraffin-embedded HEK-293A cell transduced with ROS1^{WT}, CD74-ROS1, ROS1^{N2224*}, and ETV6-NTRK3 to demonstrate specificity of ROS1 D4D6 antibody.

B Tumor growth of NIH-3T3 ROS1^{D2113N} cells subcutaneously injected into male Nu/J mice and treated for 14 days with vehicle, crizotinib (100 mg/kg), or lorlatinib (3 mg/kg). $N = 5$ apart from mice treated with crizotinib ($N = 3$), biological replicates. Two-way ANOVA with Dunnett's multiple comparisons test used to assess statistical significance (alpha 0.05). *P* value comparing vehicle vs. crizotinib (same value as vehicle vs. lorlatinib) on day 16 indicated within graph.

C Diagram showing generation of NIH-3T3 P1-ROS1^{D2113N} cell line from subcutaneous tumor in Nu/J mice.

D Immunoblots on NIH-3T3 P1-ROS1^{D2113N} cell lysates generated after treatment of cells with 10, 100 and 500 nM crizotinib, entrectinib and lorlatinib and immunoblotting with phospho-site specific antibodies as indicated in panel.

E Densitometry of replicate immunoblot experiments for evaluating effects of ROS1 TKI in NIH-3T3 P1-ROS1^{D2113N} tumor derived cells on AKT and S6 phosphorylation. $N = 2$ biological replicates except for phospho-STAT3, which is $N = 1$ because of technical failure in replicate experiment.

Source data are available online for this figure.

© 2023 The Authors
