# Supplementary material for: Discovery of oncogenic ROS1 missense mutations with sensitivity to tyrosine kinase inhibitors
Source: EMBO Mol Med. 2023 Aug 17;15(10):e17367. doi: 10.15252/emmm.202217367 (PMC10565643; doi:10.15252/emmm.202217367)
Supplement: Supplementary file 3 — Movie EV2 [file EMMM-15-e17367-s004.zip › Movie EV2/Movie EV2 Legend.docx]

Movie EV2 – Proliferation of MCF10A cells transduced with ROS1^WT^.
