# Supplementary material for: Discovery of oncogenic ROS1 missense mutations with sensitivity to tyrosine kinase inhibitors
Source: EMBO Mol Med. 2023 Aug 17;15(10):e17367. doi: 10.15252/emmm.202217367 (PMC10565643; doi:10.15252/emmm.202217367)
Supplement: Supplementary file 5 — Movie EV4 [file EMMM-15-e17367-s007.zip › Movie EV4/Movie EV4 Legend.docx]

Movie EV4 – Proliferation of MCF10A cells transduced with ROS1^D2113N^.
