# Supplementary material for: Discovery of oncogenic ROS1 missense mutations with sensitivity to tyrosine kinase inhibitors
Source: EMBO Mol Med. 2023 Aug 17;15(10):e17367. doi: 10.15252/emmm.202217367 (PMC10565643; doi:10.15252/emmm.202217367)
Supplement: Supplementary file 11 — PDF+ [file EMMM-15-e17367-s001.pdf]

Article

SOURCE DATA | TRANSPARENT PROCESS | OPEN ACCESS

EMBO Molecular Medicine

Discovery of oncogenic *ROS1* missense mutations with sensitivity to tyrosine kinase inhibitors

Sudarshan R Iyer[1,†], Kevin Nusser[1,†], Kristen Jones[1,†], Pushkar Shinde[2], Clare Keddy[1], Catherine Z Beach[1], Erin Aguero[1], Jeremy Force[3], Ujwal Shinde[2] & Monika A Davare[1,*]

Abstract

ROS1 is the largest receptor tyrosine kinase in the human genome. Rearrangements of the ROS1 gene result in oncogenic ROS1 kinase fusion proteins that are currently the only validated biomarkers for targeted therapy with ROS1 TKIs in patients. While numerous somatic missense mutations in ROS1 exist in the cancer genome, their impact on catalytic activity and pathogenic potential is unknown. We interrogated the AACR Genie database and identified 34 missense mutations in the ROS1 tyrosine kinase domain for further analysis. Our experiments revealed that these mutations have varying effects on ROS1 kinase function, ranging from complete loss to significantly increased catalytic activity. Notably, Asn and Gly substitutions at Asp2113 in the ROS1 kinase domain were found to be TKI-sensitive oncogenic variants in cell-based model systems. *In vivo* experiments showed that ROS1 D2113N induced tumor formation that was sensitive to crizotinib and lorlatinib, FDA-approved ROS1-TKIs. Collectively, these findings highlight the tumorigenic potential of specific point mutations within the ROS1 kinase domain and their potential as therapeutic targets with FDA-approved ROS1-TKIs.

Subject Categories Cancer; Molecular Biology of Disease; Signal Transduction

DOI 10.15252/emmm.202217367 | Received 27 December 2022 | Revised 24 July 2023 | Accepted 26 July 2023 | Published online 17 August 2023

EMBO Mol Med (2023) 15: e17367

Introduction

Targeted therapy has revolutionized the treatment of cancers characterized by abnormal receptor tyrosine kinase (RTK) signaling, yielding remarkable clinical outcomes during the last two decades (Schram *et al*, 2017; Drilon *et al*, 2021). Normally, RTKs facilitate the connection between extracellular signals and intracellular signaling pathways, thereby influencing diverse cellular processes, including but not limited to cell proliferation, differentiation, and metabolic changes (Lemmon & Schlessinger, 2010). Constitutive activation of RTKs leads to dysregulation of several pathways linked to the hallmarks of cancer (Du & Lovly, 2018; Hanahan, 2022). The development of tyrosine kinase inhibitors (TKIs) has been pivotal for suppressing oncogenic kinase activity in tumor cells, thereby effectively halting the constitutive upregulation of cell proliferation signaling. Numerous clinical trials have provided compelling evidence of the robust efficacy of precision targeted TKIs in reducing cancer burden among patients (Cocco *et al*, 2018; Landi *et al*, 2019; Drilon *et al*, 2020, 2021).

Diverse germline or somatic aberrations result in a gene gaining oncogene characteristics that drive malignant transformation. A well-established example is the gain-of-function nonsynonymous point mutation in EGFR, L848R, which confers constitutive catalytic activity that promotes oncogenic signaling (Sharma *et al*, 2007; Brewer *et al*, 2013). The clinical implementation of next-generation sequencing (NGS) for tumor analysis, alongside development of publicly available datasets from consortium efforts such as The Cancer Genome Atlas Project, unveiled a plethora of novel somatic variants in known protooncogenes. However, the majority of these somatic variants remain classified as variants of unknown significance (VUS). Determining whether a specific variant contributes to cancer pathogenesis or is a functionally neutral "passenger" variant is a significant challenge limiting the utility of NGS data for clinical translation. Several *in silico* algorithms have been developed to predict the impact of mutations on protein structure and function (Reva *et al*, 2007, 2011; Thusberg & Vihinen, 2009; Sim *et al*, 2012; Vaser *et al*, 2016). However, based on our experience, these algorithms are neither sufficiently accurate nor reliable to replace the need for functional validation in laboratory settings. A more practical approach involves an initial *in silico* prediction step to filter the variants and generate a smaller, more manageable list for subsequent laboratory-based functional testing.

Like other RTKs, the protooncogene *ROS1*, has demonstrated the ability to facilitate oncogenic transformation when it is aberrantly activated (Drilon *et al*, 2021). Currently, the involvement of ROS1 in cancer focuses on oncogenic ROS1 fusion proteins generated by chromosomal rearrangements. These ROS1 fusion oncogenes harbor

1 Division of Pediatric Hematology/Oncology, Department of Pediatrics, Papé Family Pediatric Research Institute, Oregon Health and Sciences University, OR, Portland, USA
2 Department of Chemical Physiology, Oregon Health and Sciences University, OR, Portland, USA
3 Department of Medicine, Division of Medical Oncology, Duke Cancer Institute, Duke University, NC, Durham, USA
*Corresponding author. Tel: +1 503 494 5056; E-mail: davarem@ohsu.edu
[†]These authors contributed equally to this work

© 2023 The Authors. Published under the terms of the CC BY 4.0 license

unchecked constitutive catalytic activities resulting from the loss of the regulatory amino-terminal domain of the receptor. ROS1 fusion proteins are established oncogenic drivers in a diverse set of cancers, particularly in lung adenocarcinomas (Arai *et al*, 2013; Saborowski *et al*, 2013; Inoue *et al*, 2016; Lin & Shaw, 2017).

Earlier preclinical studies established that tyrosine kinase inhibitors (TKIs) effectively target oncogenic ROS1 fusion proteins (Davies *et al*, 2012; Davare *et al*, 2013). Notably, substantial clinical responses were achieved with the TKIs crizotinib and entrectinib in ROS1-fusion-positive non-small cell lung cancer (NSCLC), leading to their FDA approval (Shaw *et al*, 2014; Drilon *et al*, 2017b). However, it remains unclear whether ROS1 missense mutations or amplifications can independently drive or contribute to tumor development (Drilon *et al*, 2021).

In this study, we hypothesized that a subset of *ROS1* nonsynonymous missense mutations could be gain-of-function variants contributing to tumor formation and responsive to treatment with TKIs. To address this hypothesis, we interrogated the AACR Genie dataset to identify ROS1 missense variants in a tumor-agnostic manner. Through *in silico* filtering strategies, we selected mutations located specifically within the tyrosine kinase domain for further investigation in wet-lab experiments (Sim *et al*, 2012; Vaser *et al*, 2016; Consortium, 2017). Using biochemical approaches, we identified gain of function ROS1 mutations, performed *in vitro* and *in vivo* transformation experiments, structural modeling including molecular dynamics simulation, and global proteomics to characterize novel oncogenic *ROS1* variants.

Results

Functional screening of clinical tumor sequencing data identifies several ROS1 variants that exhibit increased catalytic activity relative to wildtype ROS1

Our study aimed to identify gain-of-function mutations in ROS1 that drive oncogenic transformation. We queried the AACR Genie database (genie.cbioportal.org), a publicly available portal that contains clinical tumor sequencing data from multiple cancer centers and cancer types (Cerami *et al*, 2012; Gao *et al*, 2013; Consortium, 2017). We found that 3.5% of samples (total samples = 138,915) in this dataset contained *ROS1* alterations (Dataset EV1; Cerami *et al*, 2012; Gao *et al*, 2013; Consortium TAPG, 2017). We prioritized somatic mutations for laboratory testing based on three criteria. First, we included only *ROS1* tyrosine kinase domain (TKD; amino acids 1945-2222 of ROS1) nonsynonymous missense mutations confirmed to be somatic or with mean allele frequency (MAF; < 0.01, 2). Second, we excluded ROS1 mutations from tumor samples that harbored > 15 total alterations (> 15 total mutation burden for samples where we had this information). Finally, we used the algorithm 'Sorting Intolerant From Tolerant' (SIFT) that predicts potential impact of mutation on protein function and included ones that showed deleterious impact. This filtering process yielded 33 missense somatic mutations in the *ROS1* TKD that we functionally characterized. As shown in Fig 1A, these 33 mutations do not cluster in any subdomain within the ROS1 TKD, such as the P-loop, C-helix, HRD motif, or the activation loop (A-loop).

Using site-directed mutagenesis, we generated 33 *ROS1* variants to compare to wildtype *ROS1* ($ROS1^{WT}$) and a negative control, ROS1 K1980E, a kinase dead variant with inability to coordinate ATP. To assess gain-of-function, we transiently transfected these ROS1 constructs into HEK293T/17 cells and immunoblotted the resulting lysates with a phospho-specific ROS1 antibody that detects the C-terminal ROS1 autophosphorylation (Y2274); this phosphosite serves as a surrogate measure for kinase catalytic activity in our studies (Fig 1B). The engineered kinase dead variant, $ROS1^{K1980E}$ did not have any detectable kinase activity, as expected. The ROS1 amino acid changes of potential interest for gain of function were: L1949F, S1986F, E2071K, D2113N, and I2151F (Fig 1C). Among these, $ROS1^{D2113N}$ exhibited the highest activity with a median 4.8-fold increase in auto-phosphorylation (range 2.02–114.3) relative to $ROS1^{WT}$. To further investigate the functional impact of perturbing the D2113 position that resides in the activation loop of the kinase, we generated other substitutions, D2113E, D2113G, and D2113Y that were subsequently also discovered in the AACR Genie sequencing data (Dataset EV1). We also engineered the D2113Q mutant to understand the structural impact of substituting an uncharged polar residue at this position. As an additional negative control, we designed a compound mutation, $ROS1^{D2113N/K1980E}$. Transient transfection and immunoblotting for ROS1 autophosphorylation showed that conservative substitution of D2113E does not alter kinase behavior, D2113Y substitution with a large hydrophobic residue introduction was deleterious for catalytic function, D2113Q was modestly activating, and the small hydrophobic substitution with D2113G robustly increased catalytic activity to the same extent as D2113N (Fig 1D and E). As expected, $ROS1^{K1980E}$ exhibited no catalytic activity, and the compound mutation D2113N/K1980E phenocopied, confirming the phosphorylation increase at ROS1 Y2274 is due to increase in intrinsic catalytic activity. Thus, our functional screening approach yielded the following potential gain-of-function variants that warranted further functional validation: ROS1 L1949F, S1986F, E2071K, D2113G/N, T2207A, I2151F. The location of these residues is mapped on the structural model of the ROS1 kinase domain (Fig EV1A). The relative frequency of activating ROS1 mutations in cancer is low (< 0.1%); curated data from AACR Genie portal is shown in Fig EV1B.

Cell-based transformation and proliferation assays reveal that $ROS1^{D2113N}$ is a gain-of-function missense variant that promotes ROS1 TKI-sensitive oncogenic growth

One of the key *in vitro* indicators of the tumorigenic potential of oncogenes is their ability to confer anchorage-independent cell growth. To investigate whether enhanced ROS1 catalytic activity of the mutants is capable of inducing neoplastic transformation, we performed NIH-3T3 soft agar colony formation assay (Arai *et al*, 2013; Davare *et al*, 2013, 2018; Borowicz *et al*, 2014). We transduced NIH-3T3 cells with ROS1 mutants, including L1949F, S1986F, E2071K, D2113N, I2151F along with $ROS1^{WT}$, as well as a negative control, $ROS1^{K1980E}$, and first assessed the autophosphorylation levels of these mutants in the stable NIH3T3 cell lines (Fig 2A). Among the ROS1 activating mutations, $ROS1^{D2113N}$ exhibited the highest catalytic activation, as indicated by ROS1 Y2274 autophosphorylation, surpassing the other ROS1 activating mutants (Fig 2A).

 © 2023 The Authors

Only the NIH-3T3 ROS1^{D2113N} cells demonstrated significant lorlatinib-sensitive induction of colony formation in soft agar compared to ROS1^{WT} cells (Fig 2B); ROS1 L1949F, S1986F and E2071K had higher colony numbers than ROS1^{WT} or negative control, ROS1^{WT}; however, these were not statistically insignificant increases. Given that ROS1 fusions are recognized oncogenes, we evaluated the oncogenic potential of the known SLC34A2-ROS1 fusion (referred to as SLC-ROS1) in comparison to ROS1^{D2113N} in

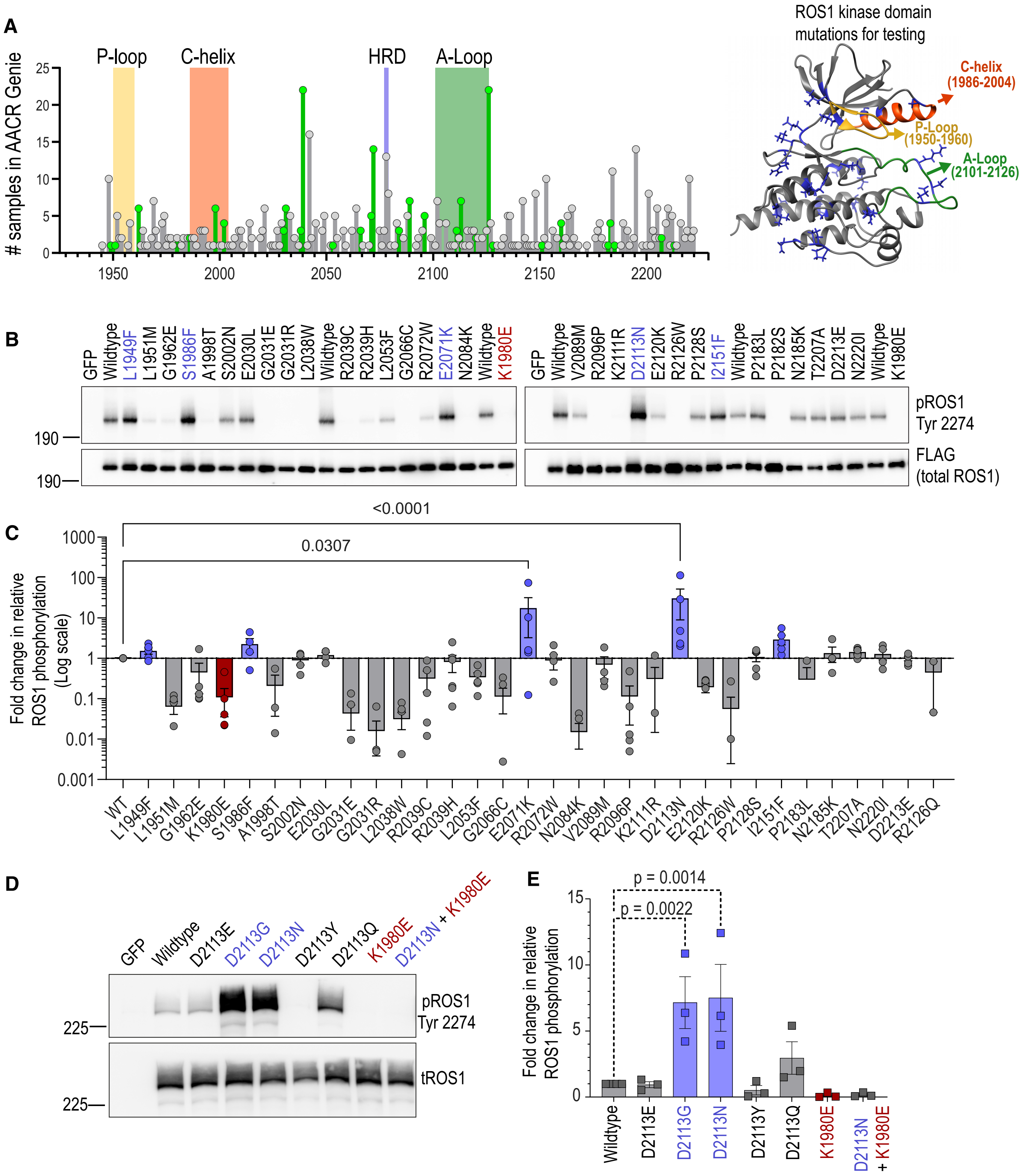

Figure 1.

Figure 1. Functional screening of missense ROS1-TKD mutations identifies potential gain of function variants.

A Lollipop diagram shows missense mutations in the ROS1 TKD from AACR Genie. *Y*-axis represents number of samples with mutation at kinase domain position while *X*-axis shows amino acid position of ROS1 (Kinase Domain: AA 1945-2222). Gray colored mutations did not pass the filtering criteria while green colored mutations were included in functional testing. Key kinase domain motifs are shaded and labeled as indicated in corresponding with the ribbon diagram of the TKD on the right.

B Representative immunoblot of transfected missense ROS1 TKD mutations in transfected HEK-293A lysates.

C Densitometry of immunoblots ($N = 3$, biological replicates) as represented in (B). Fold-change in relative ROS1 phosphorylation is calculated by first calculating the ratio of phospho-ROS1 to total ROS1 protein for each variant and then normalizing to ROS1^{WT}.

D Representative immunoblot of ROS1 D2113 position substitutions (E, G, Y, and Q as indicated) in HEK-293A cells.

E Densitometry of immunoblots ($N = 3$, biological replicates) as represented in (D). pROS1- phospho-ROS1, tROS1- total-ROS1. One-way ANOVA and Sidak's test were used to control for multiple comparisons in (C) and (E). Error bars in figure represent mean ± SEM.

Source data are available online for this figure.

this experimental model system. As shown in Fig 2C, SLC-ROS1 exhibited superior oncogenicity with nearly five-fold higher colony count than ROS1^{D2113N}.

To assess the transformative potential of ROS1^{D2113N} in an independent model system, we employed the immortalized MCF10A mammary epithelial cell line, known for its dependence on epidermal growth factor (EGF) and insulin for proliferation. The MCF10A model has been widely utilized to investigate the functional consequences and oncogenic properties of proto-oncogenes, including variants in PI3KCA (Debnath *et al*, 2003; Isakoff *et al*, 2005) and PDGFRA (Ip *et al*, 2018). For this study, we generated stable MCF10A cell lines through viral transduction using constructs for EV and ROS1 WT, K1980E, S1986F, E2071K, D2113N, D2113G. Notably, among the stably transduced MCF10A cells, only ROS1^{D2113N} and ROS1^{D2113G} exhibited detectable increase in Y2274 autophosphorylation using the same antibody dilutions as previous experiments (Fig 2D). We investigated the role of ROS1^{D2113N} and ROS1^{D2113G} in regulating cell proliferation in MCF10A cells cultured in 0.01 ng/ml EGF, a concentration 1,000-fold lower than that of the regular MCF10 growth medium containing 10 ng/ml EGF. These experiments were conducted in comparison with Empty Vector (EV), ROS1^{WT}, and oncogenic CD74-ROS1, and SLC34A2-ROS1 ROS1 fusions. Using the Incucyte® real-time imaging and analysis platform, we found that the rate of MCF10A proliferation was significantly increased with ROS1^{D2113G} and ROS1^{D2113N} expression as compared to WT or EV expression (Fig 2E). Representative images after 106 h of live imaging are shown in the right panel (Fig 2F). Congruent with NIH3T3 soft agar data, the ROS1 fusions, SLC-ROS1 and CD74-ROS1 stimulated cell proliferation to a greater extent than activating ROS1 mutations (Fig 2E and F). Expression of ROS1^{WT} is insufficient to accelerate EGF-independent cell proliferation (Fig 2G). Movies EV1–EV6 consist of cell proliferation movies from live imaging studies. To confirm that increased cell proliferation is causally linked to higher ROS1 catalytic activity, we performed MCF10A cell proliferation experiments with or without concurrent ROS1 TKI treatment with crizotinib, lorlatinib, and NVL-520, and found that all inhibitors attenuated MCF10A proliferation (Fig 2H–J). Notably, NVL-520, the most recently developed ROS1 TKI with the highest selectivity and robust potency to inhibit ROS1 (Drilon *et al*, 2023), completely blocked proliferation of MCF10A ROS1^{D2113N}, ROS1^{D21113G} and SLC34A2-ROS1 cells. Thus, these inhibitory effects on cell proliferation are unlikely to be attributable to off-target inhibition of other pathways. At the termination of the live-imaging experiment, we assessed cell viability using the water-soluble tetrazolium salt (WST-8/CCK-8 kit), a colorimetric assay. The data presented in Fig EV1C–F corroborate the findings from the live-imaging studies performed with the Incucyte® system.

Structural modeling reveals that Asn substitution at the ROS1 D2113 position dramatically increasing local flexibility within that region of the A-loop in DFG-in kinase conformation

We aimed to understand the structural basis of the ROS1 catalytic activation induced by the D2113N mutation. To this end, we developed structural models of ROS1^{WT} and ROS1^{D2113N} in their 'active' or DFG-in and 'inactive' of DFG-out states. The highly conserved residues 'DFG' appear at the start of the activation loop and the positioning of the DFG motif broadly dictates the active or inactive conformation of the kinase (Modi & Dunbrack, 2019). Changes in catalytic activity due to mutations can result from altered structure, stability, and dynamics. Here, we performed molecular dynamics simulations of ROS1^{WT} and ROS1^{D2113N} DFG-in and DFG-out structural models to measure conformational changes and structural stability. Six representative conformations for four kinases (WT: DFG-in and DFG-out and D2113N: DFG-in and DFG-out) are depicted in Fig EV2A–D. Initial observations with root mean square deviation (RMSD) analysis revealed that the ROS1^{D2113N} mutation affords dramatically enhanced protein flexibility surrounding the activation loop (A-loop) region of the kinase, specifically in the DFG-in conformation (Fig EV2E), as compared to the P-loop or αC-helix subdomains in DFG-in (Fig EV2F–G). Notably, ROS1^{D2113N} does not appear to influence RMSD in the DFG-out conformation for any sub-domains in the kinase domain (Fig EV2H–J). ROS1^{WT} and ROS1^{D2113N} remained stable during simulations with global changes in the kinase domain as shown in Total RMSD in Fig EV2E, F, H, and I (WT Total, D2113N Total).

To quantify the differences in local residue fluctuation more closely, we examined the RMSF (Root Mean Square Fluctuation) at each residue over the simulation course: mobile residues would display higher averaged RMSF values while constrained ones would display lower RMSF values (Martínez, 2015). As shown in Fig 3A. RMSF analysis suggests that in ROS1^{WT} kinase domain, the DFG-in (active) and DFG-out (inactive) conformations display dramatic differences in activation loop mobility (black lines in Fig 3A), with the DFG-out (inactive) conformation displaying far greater mobility of the activation loop than the DFG-in conformation. Intriguingly, ROS1^{D2113N} inverts this trend, and now the DFG-in conformation's A-loop displays larger flexibility. The P-loop and αC-helix, as seen in RMSD analysis, do not exhibit any substantial changes in fluctuations (Fig 3C and D), strongly suggesting that the mutation-induced hypermobility is restricted to the A-loop. The lack of overt changes

© 2023 The Authors

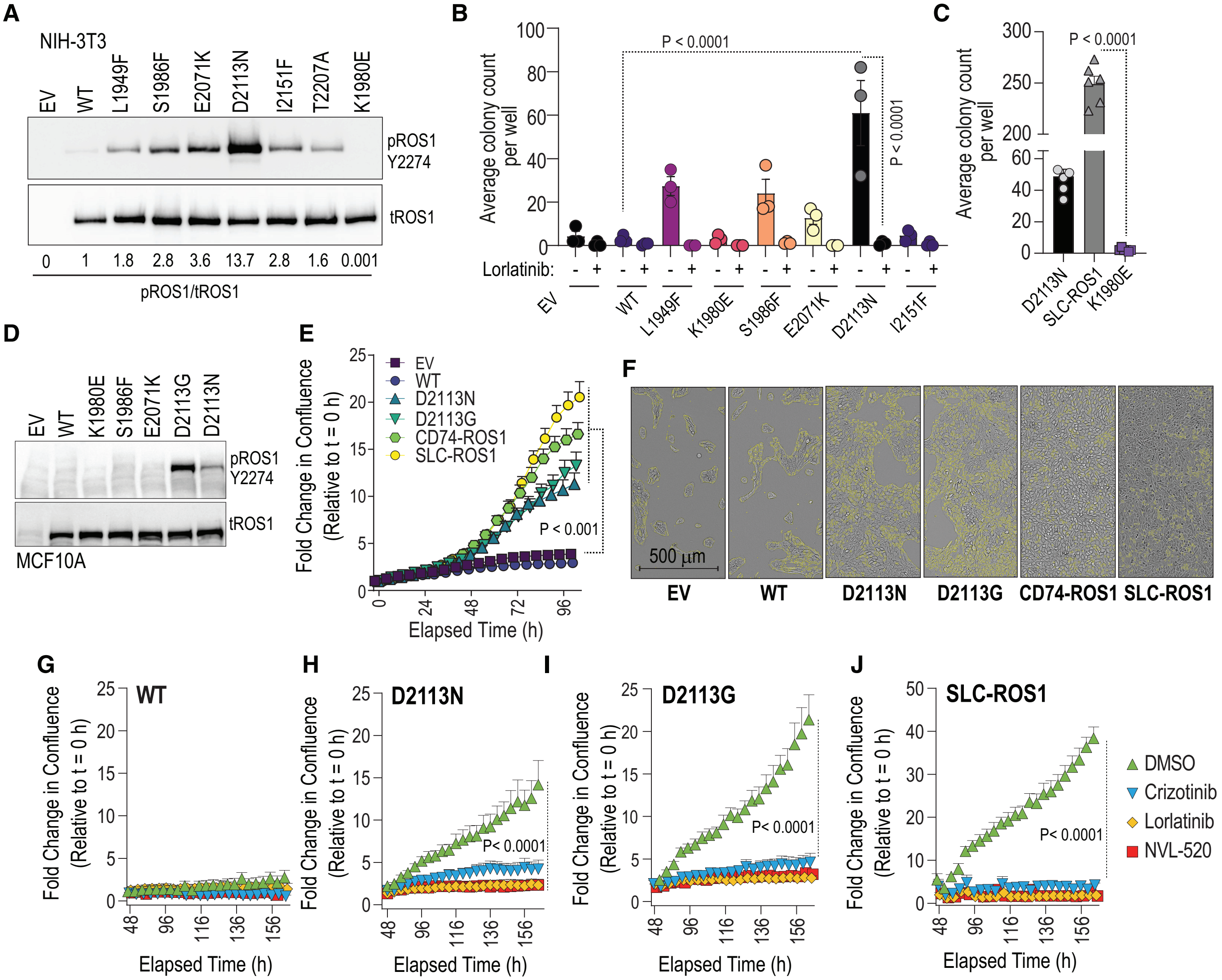

Figure 2. Cell-based transformation assays with catalytically activated ROS1 TKD variants.

A Immunoblot analysis of phospho-ROS1 and total ROS1 protein expression of NIH-3T3 cells stably transduced with empty vector (EV) or indicated ROS1 TKD mutants.

B Soft-agar assay of NIH-3T3 ROS1 cell lines (*N* = 3, biological replicates) treated with DMSO or 50 nM lorlatinib for 3 weeks. Two-way ANOVA with multiple comparisons test was used to determine statistical significance comparing $ROS1^{WT}$ colony growth to ROS1 variants with *P* values as indicated. +/− lorlatinib treated samples (*N* = 3) were compared for significance using two-way RM ANOVA (matching across rows) with Šídák's multiple comparisons test (*P* values are indicated in figure).

C Soft-agar assay of NIH-3T3 $ROS1^{D2113N}$ compared with NIH-3T3 SLC-ROS1 fusion cells (positive control) or $ROS1^{K1980E}$ (kinase-dead, negative control). +/− lorlatinib treated samples were compared for significance using two-way RM ANOVA (matching across rows, *N* = 3, biological replicates) with Šídák's multiple comparisons test (*P* values are indicated in figure).

D Immunoblot analysis of phospho-ROS1 and total ROS1 protein expression in MCF10A cells stably transduced with indicated ROS1 variants.

E Real-time MCF10A cell proliferation assay with reduced EGF (0.01 ng/ml) performed using the Incucyte® live-cell imaging system. For all cell lines, all confluence data (*N* = 4 wells that are biological replicates [with 5 fields imaged per well]) were normalized to the first scan (*t* = 0 h). Ordinary one-way ANOVA (Alpha = 0.05) was used to assess statistical difference at the hour 108 after the start of imaging. Šídák's multiple comparisons test showed significant differences as indicated by *P* values in figure.

F–J (F) Representative image taken from Incucyte platform with confluence mask definition (indicated by blue outline) was used to calculate % confluence values at indicated time point. Cell proliferation data showing effects of treatment with DMSO (Vehicle), crizotinib (250 nM), lorlatinib (250 nM), and NVL-520 (250 nM) in MCF10A ROS1 wildtype (WT) (G), D2113N (H), D2113G (I) and SLC34A2-ROS1 (SLC-ROS1) (J) as indicated. Ordinary One-way ANOVA with Dunnett's multiple comparisons test was used to determine significant differences. *N* = 6 wells, biological replicates (H, I). Error bars in figure represent mean ± SEM.

Source data are available online for this figure.

© 2023 The Authors

in the P-loop and αC-helix also serve as an internal negative control and a landmark for the kinase domain.

Next, we hypothesized that these dramatic fluctuations in the A-loop of the D2113N mutant kinase will ultimately influence the kinase pose. To assess this we performed principal component analysis (PCA) on the ensemble of poses and used clustering algorithm to discern hypothesized conformational clusters. Data in Fig 3D and E demonstrate that ROS1^{WT} and ROS1^{D2113N} exhibit greatest difference in conformation when in the DFG-in position as compared to when they are in the DFG-out position. These data are consistent with RMSD and RMSF findings and strongly indicate that the ROS1^{D2113N} in DFG-in conformation is a different kinase than ROS1^{WT}.

To quantify the difference in A-loop fluctuations between ROS1^{WT} and ROS1^{D2113N}, we subtracted the RMSF values between - DFG-in and DFG-out confirmations, and between mutations (D2113 or N2113), and plotted the differences (Fig 3F and G). An impressive, nearly four-fold difference local fluctuation at or surrounding the D2113 residue is noted between ROS1^{WT} and ROS1^{D2113N} in the DFG-in conformation but not in the DFG-out conformation as expected (Fig 3F and G). The modeled structures provide another intriguing angle. In general, these structures are colored by their surface electrostatic potential: blue suggests positive charge; red suggests negative charge; gray suggests neutral (Fig 3H–K). The mutated residue (2113) is colored green, while phosphorylatable tyrosines (2110, 2114-5) are colored yellow. In the ROS1^{WT} DFG-out structure, the negatively charged D2113 may be pulled into a positively charged pocket produced by several basic residues (R2116, R2118, K2111, and possibly R2107; Fig 3I). This conformation appears to be stabilized not by interactions of the aspartate but by several cation:pi interactions with Y2114 (Fig 3H). The ROS1^{D2113N} mutation alters the DFG-out inactive form moderately; here, the N2113 residue does not appear to come up as far into the pocket as its D2113 counterpart (Fig 3K). However, this mutation dramatically alters the active DFG-in conformation; the model suggests that the cation pi interaction is not formed in the mutant kinase, and the activation loop autophosphorylation tyrosine, Y2114/Y2115 are in a different and potentially more exposed position relative to the kinase domain (Fig 3J). Taken together, these structural modeling data suggest that the ROS1^{D2113N} mutation exerts a major dynamic effect by increasing the flexibility of the A-loop in the active (DFG-in) conformation. An aspartate to asparagine mutation at the residue 2113 position represents the smallest possible structural change. In this context, change to a glycine 2113 represents one of the largest possible change in residue size. Given our data, we envision that the glycine 2113 mutation would even further increase the active form A-loop flexibility. Thus based on knowledge from D2113N mutation, we propose that similar dynamic changes in the A-loop of the DFG-in conformation will drive activation of the D2113G mutant kinase.

Phosphoproteomics analysis uncovers both similarities and differences in effector pathway activation when comparing ROS1^{D2113N} and SLC34A2-ROS1 fusion to ROS1^{WT}

ROS1 fusion proteins have been shown to activate several effector pathways, including PTPN11 (SHP2), RAS/MEK/ERK, JAK/STAT, and in some cases, AKT/MTOR. Notably, it has been previously shown that the N-terminal fusion partner drives subcellular localization, which accordingly modulates the robustness of signaling pathway activation downstream of different ROS1 fusions (Neel *et al*, 2019; Keddy *et al*, 2022).

Since it is unknown if activated, oncogenic ROS1 full-length receptor signals similarly to ROS1 fusions, we chose an unbiased approach and performed global proteomics and phosphoproteomics (IMAC and phosphotyrosine enrichment) on HEK-293A cells that were stably transduced with ROS1^{WT}, ROS1^{D2113N} and SLC34A2-ROS1. Dataset EV2 has requisite methodological details as well as the raw data files from these studies. Both IMAC and pTyr phosphoproteomics revealed numerous statistically significant differences in a host of key downstream signaling proteins (Fig 4A and B; Dataset EV2) when comparing differences between ROS1^{WT} and ROS1^{D2113N}. Increased phosphorylation and activation were observed in the RTK adapter proteins PTPN11 (SHP2) and GAB1, the transcription factors STAT1 and STAT3, RICTOR (contributor to the mTORC2 signaling complex), and ribosomal protein s6 (downstream effector of mTOR signaling; Fig 4A and B; Dataset EV2).

To more broadly understand the effects of ROS1^{D2113N} on downstream signaling pathways, we performed Kinase-Substrate Enrichment Analysis (KSEA) (Casado *et al*, 2013; Horn *et al*, 2014; Hornbeck *et al*, 2014; Wiredja *et al*, 2017) on the IMAC phosphoproteomic data. Briefly, KSEA is a web-based tool that infers activity of kinases in given phosphoproteomic datasets by scoring each kinase based on the relative abundance of its substrates (phospho-peptides ≤ of substrates in dataset); the substrates are inferred from phosphosite-specific databases and negative or positive value of the kinase *Z*-score implies upregulation or downregulation of the kinase's activity in comparison to control. We set the following parameters for KSEA analysis: used the PhosphoSitePlus + NetworkKIN kinase-substrate datasets for predictions, set the NetworkKIN score cutoff to 3 for ROS1^{D2113N} and 5 for SLC34A2-ROS1 data, and set the substrate count cutoff to 5 for both datasets. The substrate count cutoff decides the minimum number of phosphorylated substrates represented in the proteomics data for inclusion in the bar plot, and only *Z*-score differences with $P \leq 0.05$ are plotted. Dataset EV2 has raw data for kinase scores as well as kinase-substrate findings. This analysis revealed activation of kinases routinely associated with other RTKs (e.g., ALK, EGFR, RET) in cancer. MAPK1 was activated but was the lowest significantly scored kinase in ROS1^{D2113N} cells (Fig 4C). Figure 4D–F shows differential expression of IMAC and pTyr antibody enriched phosphopeptide targets, and KSEA analysis in SLC-ROS1 expressing cells. The ROS1 fusion more robustly increased global phosphorylation of targets as seen with via the abundance of significantly upregulated phopsho-peptides in the volcano plots, as compared to ROS1^{D2113N} (Fig 4D and E). KSEA analysis suggests that SLC34A2-ROS1 may have more productive activation of the MAPK1 and less productive activation of JAK/STAT pathway as compared to ROS1^{D2113N}, indicated by the kinase *Z*-scores (Fig 4F). The upregulated and downregulated kinases that are commonly regulated between ROS1^{D2113N} and SLC34A2-ROS1 are shown in red color font.

We also conducted a comparison of global changes in protein expression between HEK293A ROS1^{D2113N} and ROS1^{WT} protein-expressing cells (Fig EV3A). Broadly, we observed that ROS1^{D2113N} upregulated the expression of proteins involved in RNA metabolism, cell cycle regulation, and the AP-1 complex transcriptional factors (Fos/NAB/ATF and Jun members). This upregulation was

 © 2023 The Authors

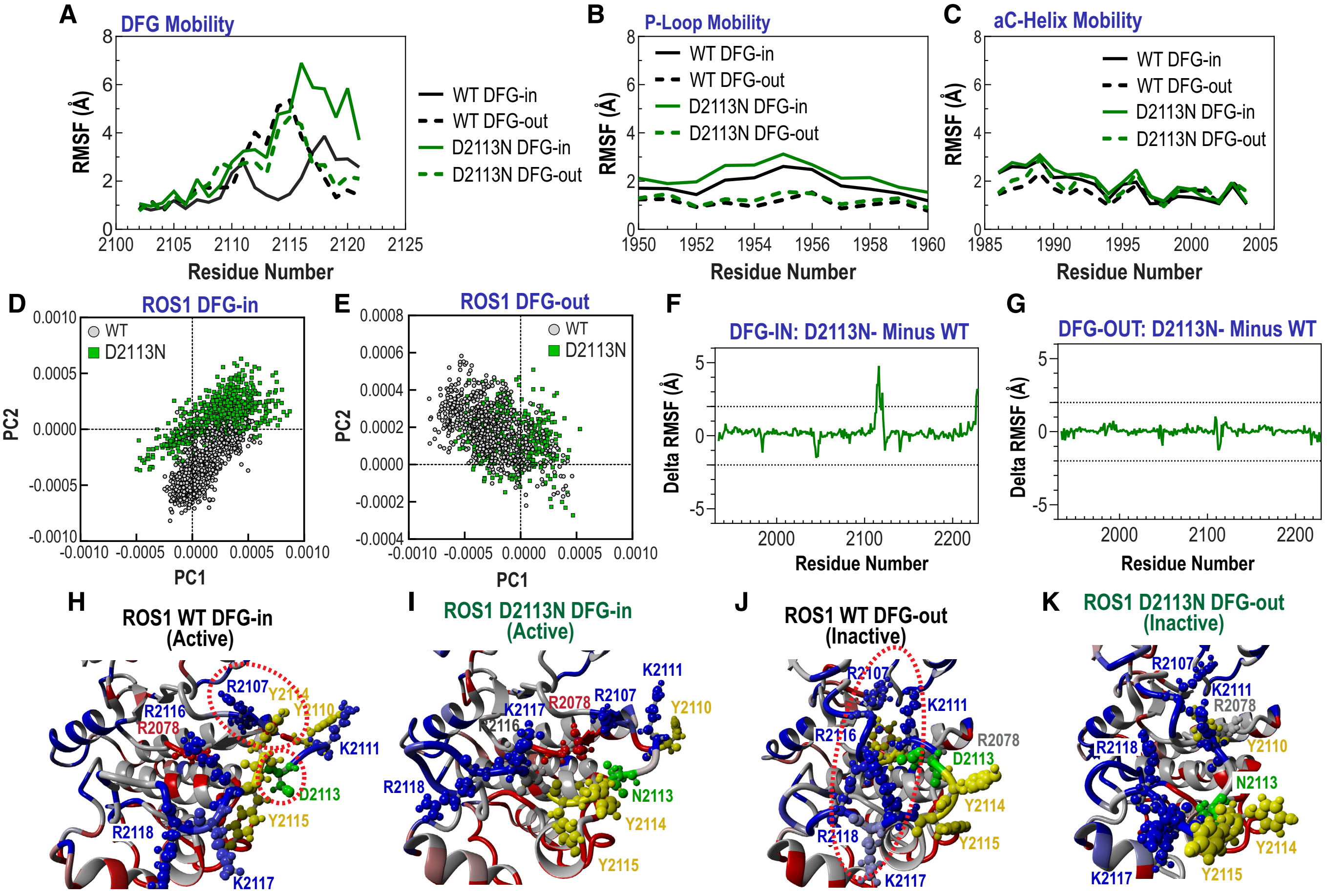

Figure 3. Structural modeling studies reveal dramatically increased dynamicity in the activation loop of ROS1^{D2113N} in DFG-in conformation.

A Root Mean Square Fluctuation (RMSF), Angstroms (Å) shown relative to residue location within A-loop from amino acid 2100 to 2125 in ROS1 wildtype (WT) and ROS1^{D2113N} (D2113N) DFG-in (active) and DFG-out (inactive) conformations.

B, C RMSF shown relative to residue location within P-loop from amino acid 1950–1960 (in B) and αC (aC) helix from amino acids 1985–2005 (in C) in ROS1 wildtype (WT) and ROS1^{D2113N} (D2113N) DFG-in (active) and DFG-out (inactive) conformations.

D, E Principal Component Analysis comparing relative distribution of ROS1^{WT} versus ROS1^{D2113N} in DFG-in (active, panel D) and DFG-out (inactive, panel E) conformations.

F Difference in RMSF of D2113N—WT in DFG-in conformation.

G Difference in RMSF of D2113N—WT in DFG-in conformation.

H–K Structural model annotated and color-coded by residue for ROS1^{WT} DFG-in (H), ROS1^{D2113N} DFG-in (I), ROS1^{WT} DFG-out (J), ROS1^{D2113N} DFG-out (K).

Source data are available online for this figure.

consistent with NGF-stimulated transcription as reported in Reactome (Gillespie *et al*, 2022). Proteins involved in oxidative phosphorylation (TCA cycle), carbon and lipid metabolism, L1 adhesion molecule-mediated signal transduction, and antigen presentation were downregulated in ROS1^{D2113N} cells. Additionally, we found a significant increase in the expression of TGFB1 and the Cysteine-rich protein 61 (CCN) family members, CCN1 and CCN2. To compare the ROS1^{D2113N} to ROS1 fusion, we analyzed proteomic expression in SLC34A2-ROS1 relative to ROS1^{D2113N} (SLC34A2-ROS1—D2113N analysis) as shown in Fig EV3B. These findings provided insights into pathways that are particularly amplified by the fusion compared to the receptor, including metabolic pathways, antigen processing, pyruvate metabolism, the p53 transcriptional gene network, Rho GTPase signaling, and EGF/EGFR signaling. ROS1^{D2113N} may have elevated signaling via VEGFR2, ROBO/SLIT, and MET receptor pathways, and appeared to upregulate genes involved in nervous system development as compared to the ROS1 fusion. We also performed unbiased pathway analysis of phosphoproteomics and global proteomics using Causalpath (Babur *et al*, 2021; Luna *et al*, 2021), which validated a link between ROS1^{D2113N} and increased SHP2, STAT, and TGFB1 signaling (Fig EV3C).

Targeted analysis of select signaling pathway activation in ROS1^{D2113N} and ROS1^{D2113G} activating mutations compared to ROS1 fusion proteins

We sought to perform targeted analysis on a select set of signaling proteins to elucidate the signaling pathways influenced by ROS1. To

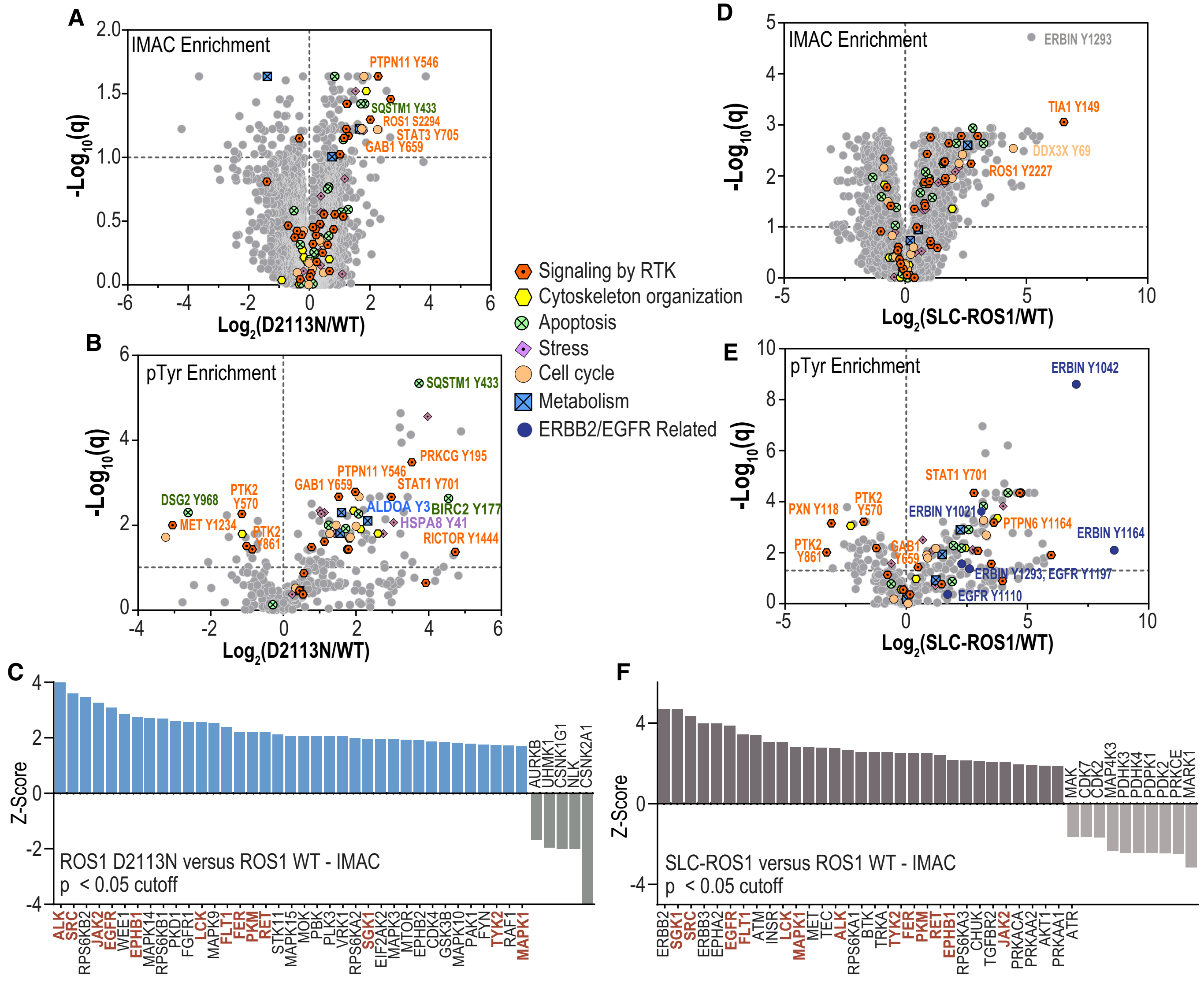

Figure 4. Phospho- and global proteomics identifies PTPN11, STAT, AP-1 transcription, and TGFB1 as ROS1^{D2113N}-upregulated signaling effector pathways.

A, B Volcano plots of statistical discoveries from IMAC-enriched (A) and phospho-tyrosine-enriched (B) phosphoproteomic spectral counts comparing ROS1^{D2113N} data to ROS1^{WT}. Select phosphosites linked to indicated cellular functions are annotated within the graph. Unpaired t tests of log2 transformed counts from ROS1^{WT} and ROS1^{D2113N} cells were conducted using Graphpad Prism with variance assumption of individual variance for each role ($N = 3$, biological replicates). Multiple Comparison tests used False Discovery Rate (FDR) with two-stage step-up (Benjamini, Krieger, and Yekutieli) with desired FDR of 5%. *X* axis features log_2-transformed ratio of normalized intensities of ROS1^{D2113N} relative to ROS1^{WT} while *y* axis features corresponding $log_{10}(q)$ values.

C Kinase substrate enrichment analysis (KSEA) of IMAC phosphoproteomic data from (A). comparing ROS1^{D2113N} to ROS1^{WT}. PhosphoSitePlus and NetworkKIN databases were queried for significant enrichment of kinase substrates analysis using KSEA. The NetworkKin score cutoff was set to 3, *P* value cutoff is 0.05 and substrate count cutoff was 5. Only kinase pathways with statistically different ($P \leq 0.05$) *Z*-scores between ROS1^{D2113N} relative to ROS1^{WT} are shown in the graph. Kinase names in red indicate differentially regulated pathways that are common between ROS1^{D2113N} and SLC34A2-ROS1 (SLC-ROS1, see D).

D, E Volcano plots of IMAC-enriched (D) and phospho-tyrosine-enriched (E) phosphoproteomic spectral counts comparing SLC23A2-ROS1 (SLC-ROS1) data to ROS1^{WT}. Unpaired t tests of log_2 transformed counts from ROS1 WT and SLC34A2-ROS1 cells were conducted using Graphpad Prism with variance assumption of individual variance for each role ($N = 3$, biological replicates). Multiple Comparison tests used False Discovery Rate (FDR) with two-stage step-up (Benjamini, Krieger, and Yekutieli) with desired FDR of 5%. *X* axis features log2-transformed ratio of normalized intensities of ROS1^{D2113N} relative to ROS1^{WT} while *y* axis features corresponding $log_{10}(q)$ values. Selected phosphosites linked to indicated cellular functions are annotated as indicated in graph legend. *X* axis features log_2-transformed ratio of normalized intensities of SLC-ROS1 relative to ROS1^{WT} while *y* axis features corresponding $log_{10}(q)$ values.

F Kinase substrate enrichment analysis (KSEA) of IMAC phosphoproteomic data from (D). comparing SLC23A2-ROS1 (SLC-ROS1) data to ROS1^{WT}. Analysis parameters identical to as described for panel (C).

© 2023 The Authors

achieve this, we performed immunoblotting using lysates obtained from transiently transfected HEK293 cells (Fig 5A). We compared the downstream activation of SHP2, ERK1/2, STAT3, c-Jun, MTOR, and S6 following transfection with ROS1^{WT}, ROS1^{D2113N}, ROS1^{D2113G}, CD74-ROS1, EZR-ROS1, and SLC34A2-ROS1 (SLC-ROS1; Fig 5A). Densitometric analysis of the immunoblots from two replicates is presented in Fig 5B.

These data show that ROS1^{D2113N} and ROS1^{D2113G} exhibited over a 50-fold increase in autophosphorylation compared to ROS1^{WT}. The three fusion proteins displayed approximately a 20–40-fold increase in autophosphorylation (phospho-ROS1/total ROS1 ratio shown). Notably, in the HEK293 cells, the ROS1 fusions led to higher levels of activation of SHP2, ERK1/2, and STAT3 when compared to the point mutations. However, activated ROS1 did not induce measurable activation of the canonical mTOR1 pathway downstream of AKT, as evidenced by the absence of increased mTOR or S6 phosphorylation.

The proteomics analysis showed notable increases in the expression levels of JunB, FosB, and FosL1 in ROS1^{D2113N} cells compared to ROS1^{WT} cells (Fig EV3). Through immunoblotting studies, we observed elevated levels of phospho-c-Jun at the well-established Ser63 site, which correlated with the higher total levels of c-Jun. These findings suggest that the observed increase in phosphorylation is merely a consequence of elevated total c-Jun expression.

To complement the gain-of-function studies, we employed a pharmacological approach to inhibit ROS1 with ROS1 TKI, lorlatinib and entrectinib, as indicated. Figure 5C presents representative immunoblot images, while Fig 5D provides densitometry data from two replicates. MAPK1 activation (ERK1/2 phosphorylation) is more robustly inhibited by ROS1 TKI in the CD74-ROS1 fusion cells as compared to ROS1 D2113N (Fig 5C and D). The comparatively weaker inhibition of ERK1/2 phosphorylation observed in the HEK293A ROS1^{D2113N} and NIH3T3 ROS1^{D2113N} cells could not be attributed to weaker inhibition of ROS1 itself or the adaptor-effector protein SHP2, as their phosphorylation is inhibited by $\geq 90\%$ in ROS1 TKI-treated cells. Interestingly, the ROS1 inhibitor lorlatinib robustly suppressed STAT3 phosphorylation in NIH3T3 CD74-ROS1 but not in HEK293A CD74-ROS1 cells; only partial inhibition of STAT3 phosphorylation was observed in HEK293A and NIH3T3 ROS1^{D2113N} mutant cells (Fig 5D).

Pharmacological approaches to elucidate ROS1 mutant and fusion-driven signaling pathways involved in EGF-independent MCF10A proliferation

Considering a some observed disparities in ROS1-mediated signaling pathway activation between NIH3T3 and HEK293 cell lines, we examined signaling pathway activation in MCF10A ROS1^{D2113N}, ROS1^{D2113G}, CD74-ROS1, and EZR-ROS1 cell lines as compared to ROS1^{WT} cells. We assessed phosphorylation of SHP2, STAT3, ERK1/2, SAPK/JNK, c-Jun, AKT, TSC2, and S6, and GAPDH as a loading control (Fig EV4A). In MCF10A cells, both ROS1 fusions and point mutations induced activation of SHP2 and STAT3; however, only CD74-ROS1 and EZR-ROS1 but not ROS1 point mutations cells had notable upregulation of pERK1/2 and pAKT in these cells. The increase in AKT activation in MCF10A CD74-ROS1 and EZR-ROS1 cells did not propagate downstream to TSC2/S6, given the absence of increased phosphorylation in these downstream targets.

To investigate the effector pathways essential for ROS1-induced cell proliferation, we conducted EGF-independent cell growth and proliferation assays in MCF10A cells expressing ROS1^{WT}, ROS1^{D2113N}, ROS1^{D2113G} and SLC34A2-ROS1 (SLC-ROS1) cells. These cells were pharmacologically treated with ROS1 inhibitors (crizotinib, lorlatinib, NVL-520, and repotrectinib), SHP2 inhibitors (TNO155 and RMC4550), MEK1/2 inhibitors (selumetinib and trametinib), AKT inhibitor afuresertib, MTOR inhibitor everolimus, JNK1/2/3 inhibitor SP600125, JAK1/2 inhibitor ruxolitinib, and TGFBR1 inhibitor galunisertib. Control groups included untreated cells, DMSO (vehicle), and a death cocktail consisting of staurosporine and YM-155, which served as a positive control for forced cell death.

Consistent with previous findings, ROS1 TKIs inhibited cell proliferation in both activated ROS1 receptor and ROS1 fusion cells (Fig EV4B–D). Also consistent with real-time proliferation assays, endpoint colorimetric assays using WST-8 reagent indicated an increase in viable cell numbers after 164 h in ROS1^{D2113N}, ROS1^{D2113G}, and SLC-ROS1 cells compared to ROS1^{WT} cells (Fig EV4E). Inhibition of SHP2 (TNO155 and RMC4550) and MEK1/2 (selumetinib) effectively blocked cell proliferation in MCF10A ROS1^{D2113N}, ROS1^{D2113G}, and SLC-ROS1 cells (Fig 6A–C). The JNK inhibitor SP600125 exhibited contrasting effects in ROS1 mutants and fusions, with decreasing inhibition from D2113N to D2113G, and no effect in SLC-ROS1 cells. A caveat of the studies employing SP600125 is that the 250 nM concentration used in these experiments is 10–20-fold lower than previously reported cell based experiments.

Unexpectedly, treatment with the pan-AKT inhibitor afuresertib resulted in an increased rate of cell proliferation in ROS1 mutants but exhibited modest inhibition in SLC-ROS1 cells (Fig 6D–F). MTOR inhibition with everolimus did not display any significant effects on these cell lines. Furthermore, differences were observed with the JAK inhibitor ruxolitinib, which inhibited cell growth in ROS1^{D2113N} and ROS1^{D2113G} but had no effect on SLC-ROS1 cells (Fig 6G–I). Since TGFB1 was found to be statistically upregulated in the global proteomics data (Fig EV3) we tested galunisertib, a TGFBR1 inhibitor, which effectively blocked cell proliferation in ROS1^{D2113N}, ROS1^{D2113G}, and SLC-ROS1 cells, albeit its effectiveness was reduced in SLC-ROS1 cells. Cell viability was assessed at end of imaging period by addition of WST-8 (Fig EV4E–J). The data presented in Fig EV4F–G support the findings on real-time proliferation shown in Fig 5. Unless otherwise noted, these differences were statistically significant. Statistical analyses for Fig EV4F–I can be found in Source Data.

In vivo studies validate that ROS1^{D2113N} promotes tumor formation that is sensitive to inhibition by ROS1-TKI treatment

We assessed the tumorigenic potential conferred by the gain-of-function ROS1 mutations as well as in-vivo efficacy of ROS1-TKI by implanting NIH-3T3 Empty Vector (EV), ROS1^{WT}, ROS1^{E2071K}, ROS1^{D2113N}, ROS1^{K1980E} (negative control), and SLC-ROS1 (positive control) cells subcutaneously into Nu/J mice. NIH-3T3 SLC-ROS1 fusion cells formed aggressive fast-growing tumors as expected. Mice injected with either EV or ROS1^{K1980E} cells did not form tumors throughout the duration of the study (74 days; Fig 7A and B). ROS1^{D2113N} cells formed palpable tumors by day 32, reaching humane tumor volume limit by day 52 (Fig 7A and B). Mice injected

© 2023 The Authors

with ROS1^{WT} or ROS1^{E2071K} cells also started to form tumors at week 6. We examined expression of ROS1 and phosphorylated effectors, pSHP2 and pSTAT3 in ROS1^{D2113N} tumors via immunohistochemistry (IHC) (Fig 7C). The D4D6 ROS1 antibody was validated for its sensitivity and specificity for ROS1 using HEK293A cell pellets from stable cell lines expressing ROS1^{WT}, CD74-ROS1 fusion, a truncated ROS1, ROS1^{N2224*} lacking the epitope for D4D6, and ETV6-NTRK3 fusion (Fig EV5A). ROS1^{D2113N} tumors express ROS1 and have upregulated phosphorylation of SHP2 and STAT3, in line with phospho-immunoblot and proteomics findings (Fig 7C). These results confirm that ROS1^{D2113N} is tumorigenic *in vivo*.

We tested if pharmacological treatment with ROS1 TKI could attenuate the tumor growth driven by ROS1^{D2113N}. For these studies, we expressed firefly luciferase via lentiviral transduction into NIH-3T3 ROS1^{D2113N} cells and performed flank injections in female Nu/J mice for tumor formation. Once palpable tumors were 90–120 mm^3 as measured with caliper, we randomized mice into vehicle, crizotinib (100 mg/kg, po, qd), and lorlatinib (3 mg/kg, po, bd)

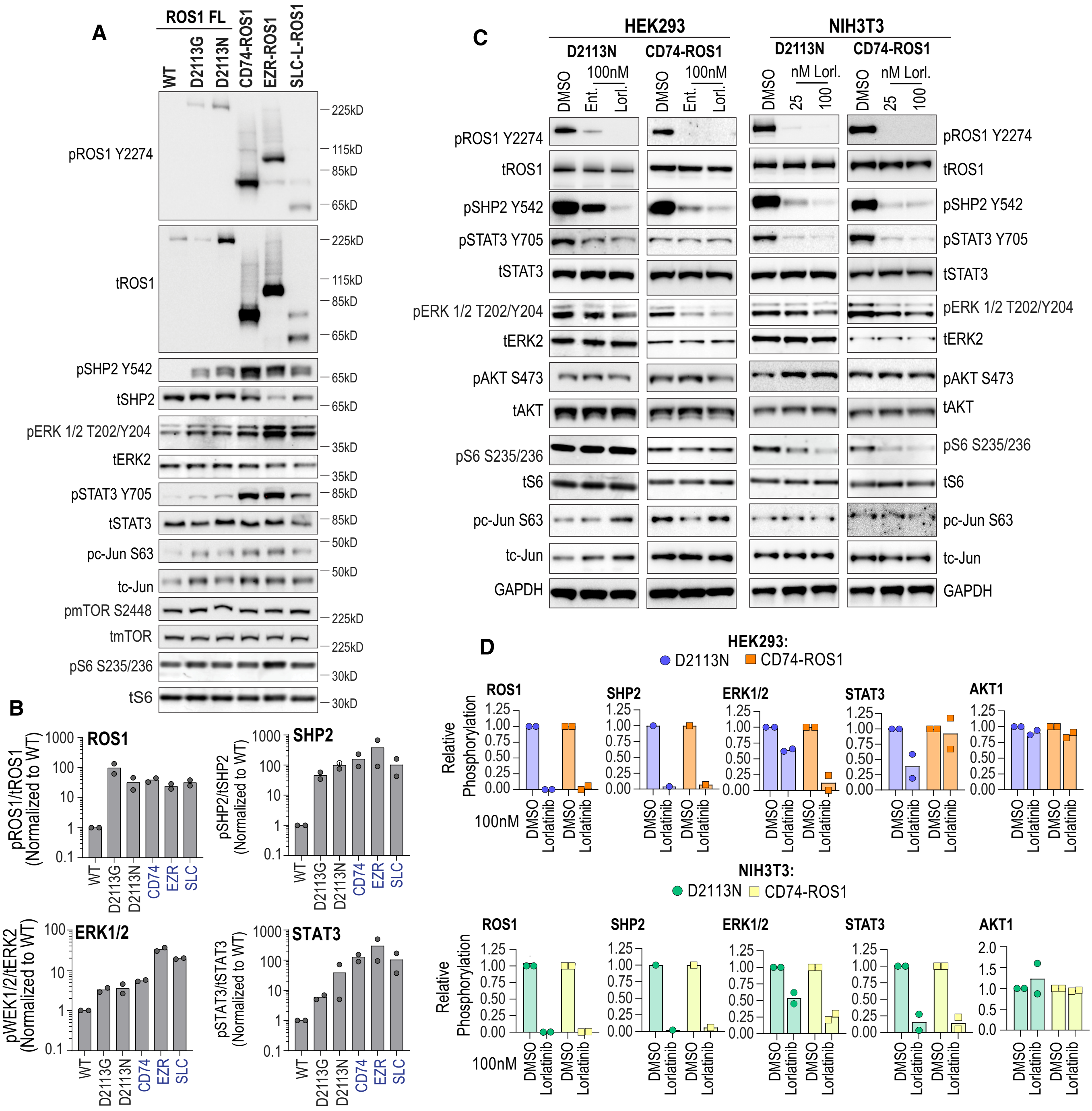

Figure 5.

© 2023 The Authors

Figure 5. Signaling pathway activation and sensitivity to ROS1 TKI in ROS1$^{D2113N/G}$ compared to ROS1 fusions expressing cells.

A Immunoblotting was performed on cell lysates generated via transient transfection of the following plasmids: pCX4 ROS1 wildtype (WT), ROS1^{D2113G} (D2113G), ROS1^{D2113N} (D2113N) and the ROS1 fusions, CD74-ROS1, EZR-ROS1 and SLC34A2-ROS1 (SLC-ROS1). The targets interrogated are indicated in the labels on the left side of the image panels and the catalog numbers as well as dilution for antibodies are reported in Methods.

B Immunoblot densitometry graphs to quantitatively compare activation of ROS1, SHP2, ERK1/2 and STAT3 (N = 2, biological replicates). Fusions are listed only by fusion partner: CD74-ROS1 (CD74), EZR-ROS1 (EZR), SLC34A2-ROS1 (SLC).

C Immunoblotting was performed on cell lysates generated from stable HEK293A ROS1^{D2113N}, HEK293A CD74-ROS1 and NIH ROS1^{D2113N} and NIH3T3 CD74-ROS1 after treatment with ROS1 TKI. HEK293A stable cell lines were treated with DMSO, 100 nM entrectinib and 100 nM lorlatinib, and NIH3T3 stable cell lines were treated with 25 nM and 100 nM lorlatinib. The targets interrogated are indicated in the labels on the left side of the image panels in HEK293 images and on the right side of image panels in NIH3T3 images.

D Immunoblot densitometry graphs to quantitatively compare extent of inhibition ROS1, SHP2, ERK1/2 and STAT3 and AKT1 phosphorylation from two independent replicate experiments. Pixel density of phosphorylated protein signal was divided by total protein signal, and this ratio was internally normalized to DMSO (Vehicle) treated cell ratio. Note that only the 100 nM lorlatinib condition that was common to the HEK293A and NIH3T3 experiments is depicted in graphs.

Source data are available online for this figure.

treatment groups (n = 3; Fig 7D). Over 2 weeks of treatment, both crizotinib and lorlatinib significantly attenuated ROS1^{D2113N}-driven tumor growth in mice relative to vehicle (Fig 7D and E) and increased survival relative to vehicle treatment (Fig 7F). To ensure that ROS1^{D2113N} is tumorigenic in both sexes and test TKI effects, we repeated this *in vivo* TKI efficacy study in male Nu/J mice (Fig EV5A). These data show that ROS1^{D2113N} is tumorigenic independent of sex and crizotinib effectively attenuates tumor formation; in these studies with male mice, lorlatinib was effective at inhibiting tumor growth but did not achieve statistical significance. Therefore, our data suggest that ROS1^{D2113N} drives tumor formation that can be attenuated or blocked with ROS1-TKI, *in vivo*, supporting the data from cell-based *in vitro* models.

We created a tumor-derived cell line from the NIH-3T3 ROS1^{D2113N} tumors called P1-ROS1^{D2113N} (Fig EV5C). P1-ROS1^{D2113N} retained ROS1 expression that was TKI-sensitive (Fig 7G and H). In terms of ROS1 TKI effects on effector signaling, the degree of SHP2 inhibition was best correlated with the inhibition of ROS1 (Fig 7G and I). In contrast, activation of ERK1/2 was nearly completely abrogated only with the higher doses of crizotinib (100 and 500 nM), but minimally affected by lorlatinib. Crizotinib inhibits multiple kinases with equipotency to ROS1, this includes MET and ALK. Given that NIH3T3 cells lack ALK, we conclude that the robust of inhibition of ERK1/2 phosphorylation in crizotinib treated cells may be due to concurrent inhibition of MET since cells treated with 100 nM crizotinib show only partial inhibition of ROS1 and SHP2 but robust inhibition of pERK1/2. In contrast to crizotinib, lorlatinib is not only a more selective inhibitor of ROS1, but also more potent (Zou *et al*, 2015). We can best appreciate the contribution of ROS1 to ERK1/2 activation in the lorlatinib treated samples, where ROS1 activation is nearly abrogated, but phospho-ERK1/2 inhibition, while statistically significant, is only partial (~ 40–60% inhibited) (Fig 7H and J). Similar to effects on ERK1/2 phosphorylation, phosphorylation of STAT3, AKT and S6 were only partially inhibited with higher doses of lorlatinib that did fully inhibit detectable ROS1 phosphorylation (Fig EV5D and E).

Discussion

The domain organization of receptor tyrosine kinases (RTKs) comprises an extracellular ligand-binding region (ECD), a single-pass transmembrane helix, and an intracellular portion consisting of a juxtamembrane domain (JM), a tyrosine kinase domain (TKD), and a C-terminal domain (CTD). RTKs play a critical role in connecting extracellular signals to intracellular signaling pathways that regulate cell proliferation, differentiation, and metabolic changes (Schlessinger, 2014). It is widely recognized that aberrant constitutive activation of RTKs leads to the upregulation of downstream signaling pathways, promoting uncontrolled cell proliferation and survival, which are key hallmarks of cancer (Hanahan & Weinberg Robert, 2011; Fleuren *et al*, 2016; Du & Lovly, 2018; Sanchez-Vega *et al*, 2018). Multiple mechanisms contribute to the hyperactivation of RTKs. Firstly, chromosomal rearrangements involving RTK genes result in chimeric fusion oncoproteins consisting of a constitutively active TKD that drive various types of liquid and solid tumors (Rikova *et al*, 2007). Several studies have identified over 55 partner genes capable of fusing via translocation or intrachromosomal deletion to the intracellular regions of the ROS1 gene, leading to the formation of fusion oncogenes (Drilon *et al*, 2021). Secondly, RTK amplification of RTK genes, results in increased expression and subsequent activation of downstream signaling pathways. Examples of this include HER2 amplification in breast cancer (Paik *et al*, 2008), *EGFR* amplification in gliomas (Smith *et al*, 2001; preprint: Ni *et al*, 2022), and *MET* amplification in non-small-cell lung cancer (NSCLC; Drilon *et al*, 2017a). Lastly, gain-of-function nonsynonymous point mutations are also recognized as mechanisms leading to RTK overactivation in cancer. While gain-of-function mutations in the ECD of RTKs (such as EGFR) have been reported, gain of function mutations in the RTK TKD are more prevalent in adult cancers (Lahiry *et al*, 2010; Medves & Demoulin, 2012; Bresler Scott *et al*, 2014).

An important challenge in translating clinical cancer genomic sequencing data is the lack of functional classification for variants of unknown significance, to allow distinguishing druggable oncogenes from passenger mutations. Therefore, validating potential gain-of-function mutations is crucial to maximize the clinical value of next-generation sequencing (NGS) data. In pursuit of this goal, numerous *in silico* algorithms have been developed to predict the impact of mutations on protein structure and function. (Reva *et al*, 2007, 2011; Thusberg & Vihinen, 2009; Sim *et al*, 2012; Vaser *et al*, 2016); However, in our perspective, these algorithms are still not precise enough to serve as a replacement for wet-lab functional validation. The risk of exclusive reliance on *in silico* approaches is revealed via this example: ALK F1174L, an oncogenic mutation in neuroblastoma (George *et al*, 2008; Mossé, 2016), is classified as a "neutral" functional impact variant by the Mutation Assessor

© 2023 The Authors

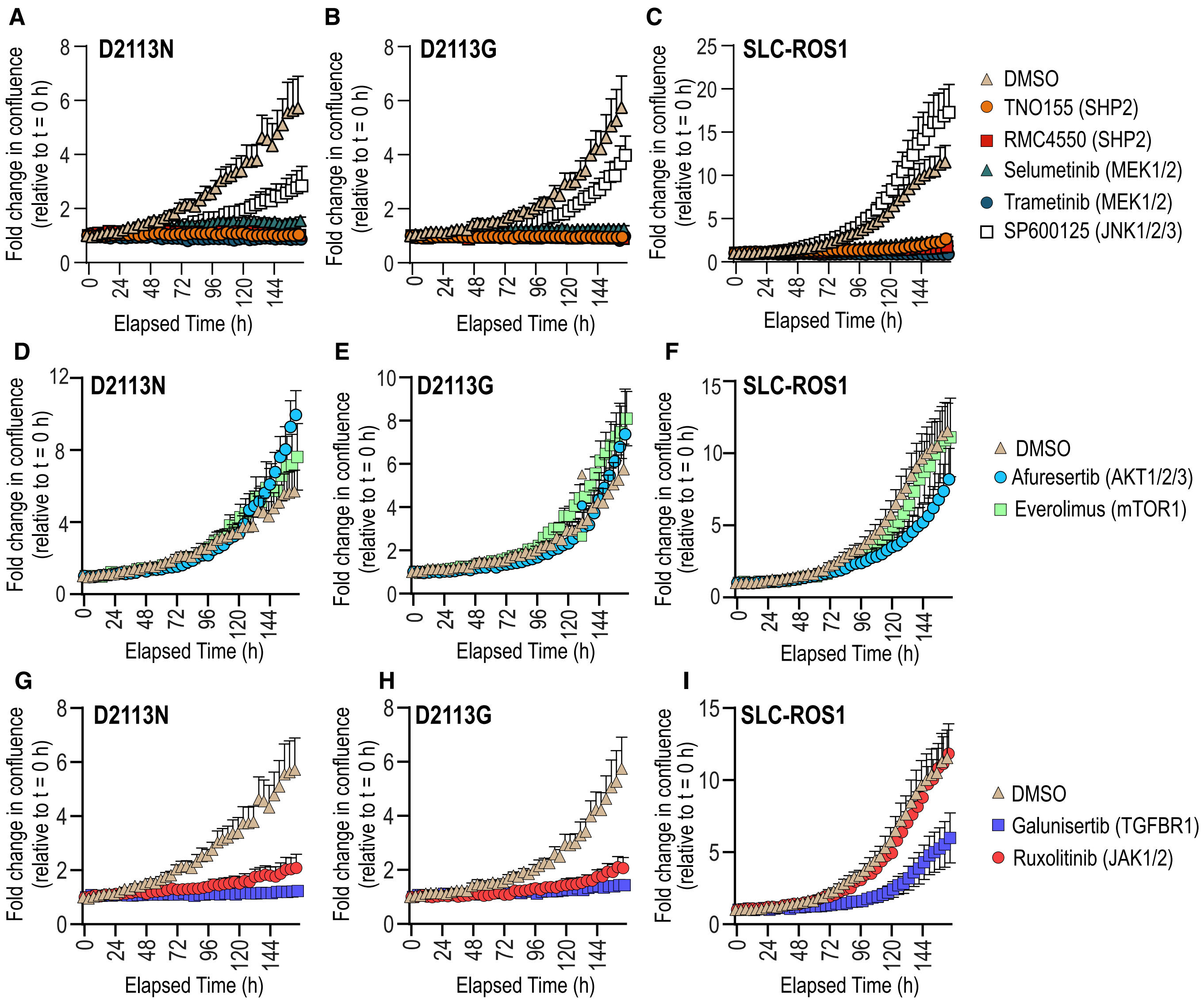

Figure 6. Signaling pathway inhibitor effects on MCF10A ROS1^{D2113N}, ROS1^{D2113G} and SLC34A2-ROS1 cell proliferation.

A–C Cell proliferation effects after treatment with SHP2 inhibitors (TNO155, RMC4550), MEK1/2 inhibitors (selumetinib, trametinib), and JNK1/2/3 inhibitor, SP600125 as indicated (N = 6, biological replicates).

D–F Cell proliferation effects after treatment with AKT1/2/3 inhibitor afuresertib, and MTOR1 inhibitor, everolimus, as indicated (N = 6, biological replicates).

G–I . Cell proliferation effects after treatment with JAK1/2 inhibitor ruxolitinib, and TGFBR1 inhibitor galunisertib (N = 6, biological replicates). All inhibitors were used at 250 nM final concentration, with the exception of galunisertib, which was used at 500 nM final concentration. Note that the DMSO data are the same in for each cell line when compared to different class of inhibitors. Error bars in figure represent mean ± SEM.

platform. Therefore, without laboratory-based validation, ALK F1174L would not have been recognized as an oncogene and a biomarker for response to next-generation ALK TKIs. *In silico* approaches may have potential in the future, especially with the advancement of functionalities incorporating machine learning or artificial intelligence, provided they are rigorously trained on datasets that include functionally validated mutations.

In this study, we identified oncogenic gain-of-function substitutions involving aspartate 2113 within ROS1 kinase domain. Our findings establish that the FDA-approved tyrosine kinase inhibitors (TKIs) crizotinib and lorlatinib effectively reduced tumor growth driven by the ROS1^{D2113N} mutation. This discovery provides a potential therapeutic approach for tumors harboring this mutation. However, it is important to note that the overall frequency of ROS1 activating mutations in the cancer genome is low, at ≤ 0.5%. Over the past decade, the basket trial design has gained prominence as a means to identify highly effective treatment options for even very rare molecular subtypes of cancer, by pooling patients with similar molecular profiles. An illustrative example is the rapid histology-agnostic FDA approval of NTRK inhibitors, such as larotrectinib and entrectinib, for the treatment of patients with validated NTRK fusions.

© 2023 The Authors

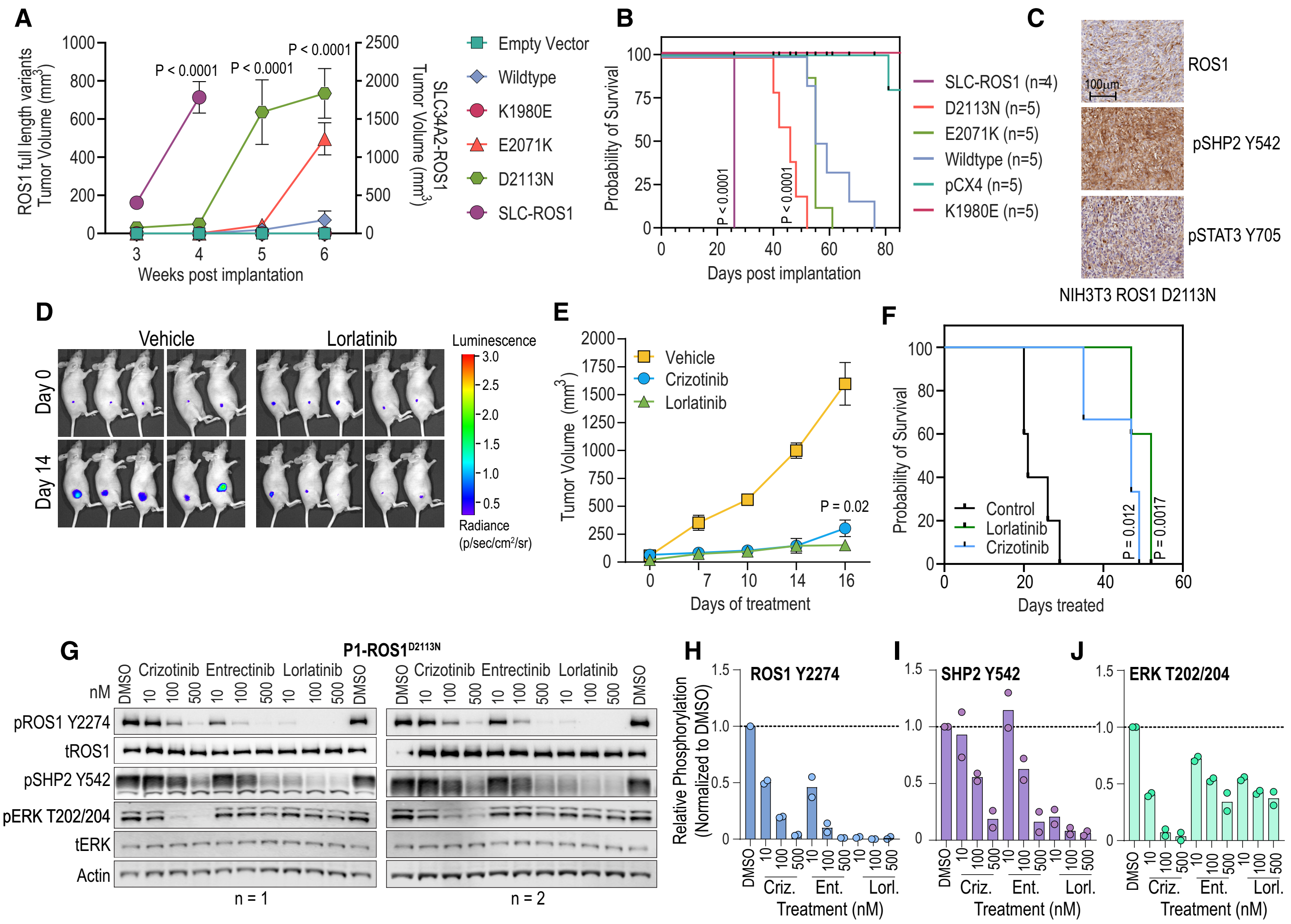

Figure 7. *In vivo* tumorigenesis studies confirm ROS1^{D2113N} as a gain-of-function oncogenic variant.

A Tumor volume (mm^3) of NIH-3T3 empty vector, ROS1^{WT}, ROS1^{K1980E}, ROS1^{E2071K}, ROS1^{D2113N}, and SLC-ROS1 cells subcutaneously injected into flank of female Nu/J mice as monitored for 6 weeks. N = 5 biological replicates, with exception of SLC34A2-ROS1 which had four biological replicates. Two-way ANOVA with Alpha = 0.05 test was used. Tukey's multiple comparison test showed statistical significance as indicated by P values within graph (SLC34A2-ROS1 or ROS1^{D2113N} vs. ROS1^{WT}). All statistical analyses are in Source Data.

B Kaplan–Meier survival curve of mice described in (A). Comparison of survival curves using Log-rank (Mantel-Cox) test was used to compare difference between EV (N = 5) and D2113N (N = 5), and EV and SLC34A2-ROS1 (N = 4); significance is indicated as P values within graph.

C Representative images of immunohistochemistry of NIH-3T3 ROS1^{D2113N} tumors stained with total ROS1, phospho-SHP2 (Y542), and phospho-STAT3 (Y705) expression.

D Representative images of photonic flux from luciferase-engineered ROS1^{D2113N} cell implanted into Nu/J mice and treated with Vehicle or lorlatinib (3 mg/kg) on days 0 and 14 of treatment. Non-invasive bioluminescent imaging achieved via IVIS Spectrum™ imaging platform.

E Tumor volume of NIH-3T3 ROS1^{D2113N} cells subcutaneously injected into female Nu/J mice (N = 3, biological replicates) and treated for 16 days with vehicle, crizotinib (100 mg/kg), or lorlatinib (3 mg/kg). Two-way RM ANOVA was used to determine significant differences (Alpha 0.05) with Dunnett's multiple comparison test. P value comparing vehicle vs. crizotinib (same value as vehicle vs. lorlatinib) on day 16 indicated within graph.

F Kaplan–Meier survival curve of mice described in (C).

G Immunoblot analysis of the phosphorylated (p) and total (t) proteins from NIH-3T3 ROS1^{D2113N} P1 cell lysates prepared from cells treated with vehicle (DMSO) or 10, 100, or 500 nM of crizotinib, entrectinib, or lorlatinib for 4 h.

H-J Immunoblot densitometry graphs to quantitatively compare extent of inhibition ROS1, SHP2 and ERK1/2 phosphorylation (panel H data) after treatment with indicated inhibitors from two independent biological replicate experiments.

Source data are available online for this figure.

We found ROS1^{D2113N} has oncogenic effects in different cell types. ROS1^{D2113N} was found in melanoma and oligodendroglioma. ROS1^{D2113G} was present in esophageal cancer (Cerami *et al*, 2012; Gao *et al*, 2013; Consortium TAPG, 2017). This suggests that activated ROS1 has the potential to be a pathogenic cancer driver in diverse cancers, aligning with the occurrence of ROS1 fusion oncogenes in a wide range of adult and pediatric cancers.

Understanding of the signaling pathways activated by constitutively activated full-length ROS1 receptor is limited. Thus, our proteomics data offer novel insight into the impact of

© 2023 The Authors

ROS1^{D2113N} on cell-signaling pathways in comparison to ROS1 fusions. Through KSEA analysis of the phosphoproteomic data, we observed that both the activated receptor and fusion upregulate similar pathways, albeit with some variations in the degree of activation. For instance, MAPK1 substrates exhibit higher rankings in SLC34A2-ROS1 fusion cells compared to ROS1^{D2113N}. However, both variants show a statistically significant increase in MAPK1 activation as compared to ROS1^{WT}. Conversely, JAK2 substrates are ranked higher in ROS1^{D2113N} compared to the SLC-ROS1 fusion. These findings suggest that the ROS1 fusions, which lack the N-terminal domain of the ROS1 receptor, do exhibit some signaling differences in comparison to the activated receptor. It is essential to note that *in vitro* studies of ROS1^{D2113N} were conducted without a ligand, the identity of which is still not definitively proven. The extent to which activation mediated by ROS1^{D2113N} is autonomous or influenced by ligand-mediated activation in its native setting remains unknown. Therefore, it is plausible that ligand binding might further enhance the oncogenicity of the ROS1 kinase mutant.

An additional intriguing finding in the context of cancer signaling that emerged from the global proteomics analysis is the upregulation of AP-1 transcription, TGFBR ligand TGFB1 and the CCN family of proteins in ROS1^{D2113N} cells. Previous literature has demonstrated a connection between TGFβ upregulation and AP-1 transcription (Derynck *et al*, 2021), as well as the upregulation of CCN proteins by TGFβ (Nakerakanti *et al*, 2011; Tejera-Muñoz *et al*, 2021). Both TGFβ and the CCN family of proteins have been implicated in poorer prognosis in cancer due to their ability to promote stem cell-like behavior, decrease cell adhesion, increase invasiveness and metastatic potential, and induce angiogenesis across a wide range of cancer types (Massagué, 2008; Haque *et al*, 2011; Lau, 2012; Kim *et al*, 2018).

Structural modeling gives initial insight into the potential mechanism via which ROS1^{D2113N} increases catalytic activity. Our data point to the dramatic impact of this mutation on local structural changes that lead to enhanced mobility within the region of the A-loop that D2113 resides in. Notably, D2113 is adjacent to key auto-phosphorylation sites, Y2114 and Y2115 that are thought to be required for sustained catalytic activation and maintenance of the kinase structure in the DFG-in or open/active kinase conformation. Thus, we can theorize that these alterations in the A-loop surrounding D2113 residue may increase propensity of the tyrosine kinase to stay in the DFG-in like state resulting in presumed enhanced ATP binding and/or accessibility for both intrinsic (Y2114/Y2115) and extrinsic substrates such as effector proteins. We previously showed that ROS1^{D2113N} and to an even greater extent, ROS1^{D2113G}, confers resistance to type II binding mode ROS1-TKI cabozantinib, in the context of ROS1 fusion protein, CD74-ROS1 (Davare *et al*, 2015). Briefly, type II inhibitors have higher affinity and binding preference for the DFG-out (inactive) kinase conformation. We established cabozantinib as a type II ROS1 inhibitor. In contrast, type I inhibitors have preferential binding to the DFG-in (active) kinase conformation. These previous data showing that the D2113N/G substitutions reduce cabozantinib sensitivity of ROS1, provides strong independent support for the conclusion that D21113 mutations activate kinase by favoring the DFG-in conformation that is not favorable for type II inhibitor binding. From a translational perspective, it will be important to test if ROS1^{D2113N} in the full-length receptor context is also resistant to type II ROS1-TKI treatment akin to these previous observations with ROS1 fusion proteins. Additionally, future crystallographic or cryo-electron microscopy studies of mutant ROS1 kinase will be essential to confirm the findings from the modeling studies and for a deeper understanding of the impact of these mutations on ROS1 kinase structure–function relationship.

Ultimately, we hope that identifying gain of function mutations among the plethora of reported variants of unknown significance in actionable genes such as ROS1 may enable an expanded cohort of patients to benefit from targeted kinase inhibitors to improve their outcomes.

Materials and Methods

Cell lines, compounds, and plasmids

HEK-293T/17 (ATCC Cat# CRL-11268, RRID:CVCL_1926), MCF10A cells (ATCC Cat# CRL-10317, RRID:CVCL_0598), and NIH-3T3 cells (ATCC Cat# CRL-1658, RRID:CVCL_0594) were purchased from American Type Culture Collection (ATCC; Manassas, VA, USA). HEK-293A cells (Cat# R70507; RRID:CVCL_6910) was purchased from Thermo Fisher Scientific (Waltham, MA, USA). Platinum-A and Platinum-E cells were purchased from Cell BioLabs, Inc. (San Diego, CA, USA). Primary antibodies used in the study are as follows: phospho-ROS1 (Cell Signaling Technology, Inc. (CST; Danvers, MA, USA); Y2274; #3078; RRID:AB_2180473), ROS1 (CST; #3287; RRID:AB_2797603), phospho-STAT3 (CST; Y705; #9145; RRID:AB_2491009), STAT3 (CST; #9139; RRID:AB_331757), phospho-ERK (CST; T202/Y204; #9101; RRID:AB_331646), ERK (CST; #4696; RRID:AB_390780), anti-FLAG (Thermo Fisher Scientific Cat# 701629, RRID:AB_2532497), α-TUBULIN (Developmental Studies Hybridoma Bank (DSHB; Iowa City, IA, USA); 12G10; RRID: AB_1157911), ACTIN (DSHB; JLA20; RRID:AB_528068), GAPDH (Santa Cruz Biotechnology, Inc.; Dallas, TX, USA; sc-47724; RRID: AB_627678), phospho-S6 (CST; S235/236, #4858; RRID: AB_916156), and S6 (CST; #2317; RRID:AB_2238583), ERK2 (Santa Cruz Biotechnology, Inc.; sc-1647; RRID: AB_627547). All antibodies with the following exceptions were diluted 1:1,000 in 5% BSA incubation buffer; Total ROS1 (CST; #3287; RRID:AB_2797603), ACTIN (DSHB; JLA20; RRID:AB_528068), and ERK2 (Santa Cruz Biotechnology, Inc.; sc-1647; RRID: AB_627547) were used at a 1:2,000 dilution. DMEM, DME-F/12, L-glutamine, and antibiotics were purchased from Genesee Scientific (San Diego, CA, USA). Fetal bovine serum (FBS) was procured from Neuromics (CA3 Biosciences, Inc., Edina, MN, USA). Bovine growth serum (BGS) was procured from VWR International (Radnor, PA, USA). Horse serum (HS) and calf serum (CS) were purchased from Thermo Fisher Scientific. Bovine pancreas insulin solution was purchased from Millipore Sigma (Cat# I0516; Burlington, MA, USA). Recombinant human EGF was purchased from Peprotech (Cranbury, MA, USA), hydrocortisone powder from Millipore Sigma (Cat# H0888), and cholera toxin from Cayman Chemical (Ann Arbor, MI, USA). 0.05% Trypsin/0.53 mM EDTA was procured from Corning Inc. (Corning, NY, USA) while TrypLE™ was purchased from Thermo Fisher Scientific. Crizotinib, cabozantinib, and staurosporine were purchased from LC Laboratories® (Woburn, MA, USA), and lorlatinib, entrectinib, repotrectinib, and cycloheximide from Selleck Chemicals Inc. (Houston, TX, USA). TransIT-LT1 transfection reagent was purchased

 © 2023 The Authors

from Mirus Bio LLC (Madison, WI, USA). Gateway LR clonase was purchased from ThermoFisher. Protease and phosphatase inhibitors were purchased from Bimake (Houston, TX, USA). Pre-cast Bolt™ and NuPAGE™ 4–12% Bis-Tris protein gels and 4X LDS sample buffer were purchased from ThermoFisher. Pierce™ BCA Protein Assay Kit was purchased from ThermoFisher. CCK-8 reagent was purchased from Bimake (Milpitas, CA, USA). Infusion Cloning HD Kit was procured from Takara Bio USA Inc. (San Jose, CA, USA). pCMV-VSV-G was a gift from Bob Weinberg (Addgene plasmid # 8454; http://n2t.net/addgene:8454; RRID:Addgene_8454), pENTR4-FLAG (w210-2) was a gift from Eric Campeau & Paul Kaufman (Addgene plasmid # 17423; http://n2t.net/addgene:17423; RRID: Addgene_17423), pGP (retroviral Pol and Rev gene plasmid) was a gift from Romel Somwar, and pCX4-puro was a gift from Tsuyoshi Akagi. Wild-type full-length ROS1 human cDNA was purchased from DNASU (Tempe, AZ, USA). Oligonucleotides were ordered from Integrated DNA Technologies Corp. (Newark, NJ, USA) and Eurofins Genomics (Eurofins Scientific, Luxembourg).

Growth and propagation of cell lines

All cell lines were grown in a tissue culture incubator with 5% CO_2 at 37°C. Cells were maintained in 75 cm^2 flasks and subcultured when approaching 75% confluence. NIH-3T3 cells were maintained in DMEM medium supplemented with 10% (vol/vol) CS and 1% (vol/vol) L-glutamine solution and detached using 0.05% Trypsin/0.53 mM EDTA. HEK-293T/17, HEK-293A, Platinum-A, and Platinum-E cells were maintained in DMEM medium supplemented with 10% (vol/vol) BGS and 1% (vol/vol) L-glutamine solution and detached using 0.05% Trypsin/0.53 mM EDTA. MCF10A cells were maintained in DMEM/F12 medium supplemented with 5% (vol/vol) horse serum (HS), 1% (vol/vol) L-glutamine solution, 0.5 mg/ml hydrocortisone, 100 ng/ml cholera toxin, 10 μg/ml insulin, and 20 ng/ml EGF (MCF10A growth medium) as previously described (Isakoff *et al*, 2005). MCF10A cells were detached using 2× TrypLE™ in 1× Versene and neutralized with MCF10A resuspension medium (DMEM/F12 supplemented with 20% [vol/vol] HS and 1% [vol/vol] L-glutamine solution). All cell lines were routinely tested for mycoplasma contamination (Lonza MycoAlertTM PLUS Mycoplasma Detection Kit) and verified to be free of contamination. Antibiotics were included in all cell culture media.

Cloning

The retroviral construct pCX4 ROS1 was cloned as described previously (Davare *et al*, 2013). Briefly, full-length ROS1 cDNA was cloned using the SalI and XhoI sites in the multiple cloning site of pENTR4-No ccDB (696-1) vector (Addgene Plasmid #17424) via In-Fusion™ cloning using PCR amplification that included the addition of C-terminal Flag tag. Simultaneously, pCX4-puro was converted into a Gateway™ Destination vector (pCX4-DEST-Puro) by inserting the Gateway™ Reading Frame Cassette A (ThermoFisher) into the multiple cloning site using EcoRI and XhoI restriction sites. ROS1-FLAG cDNA was subcloned into pCX4-DEST-Puro via LR clonase reaction. ROS1 mutants were generated using site-directed mutagenesis (QuikChange™ Mutagenesis Protocol, Agilent Technologies Inc., Santa Clara, CA, USA) of the pENTR4-ROS1-FLAG construct and subsequently subcloned into pCX4-DEST-Puro.

Generation of stable isogenic cell lines

Replication incompetent, infectious ecotropic and VSV-G pseudotyped amphotropic retroviral particles were generated using Platinum-E and HEK293T/17 cells or Platinum A cells, respectively. Platinum-E cells were transfected in 6-well plates with 2 μg pCX4 ROS1 transfer plasmid complexed with 8 μl TransIT-LT1 reagent while HEK293T/17 cells were transfected in 10 cm dishes with 6 μg transfer plasmid, 5 μg pGP, and 4 μg pCMV-VSV-G complexed with 45 μl TransIT-LT1 reagent. Ecotropic viral supernatant collected at 48- and 72-h post transfection was filtered with 0.45 μm syringe filter, and used for transduction of cells pre-treated with 2 μg/ml polybrene. VSV-G pseudotyped viral supernatant was concentrated via ultracentrifugation and used for cell transduction. Transduced cells were selected with 1 μg/ml puromycin for 4 days.

Immunoblotting

Lysates were prepared from cells using a standard cell lysis buffer as described before (Davare *et al*, 2015). Protein quantitation was performed with the Pierce™ BCA Protein Assay Kit. For immunoblotting, we loaded 15 μg of reducing LDS sample buffer-extracted cleared cell lysates on pre-cast 4–12% Bolt™/NuPAGE™ Bis-Tris gels. Spectra Multicolor Broad Range Protein Ladder (Thermo Fisher Scientific) was used to determine relative molecular weights of protein bands after imaging. Proteins were transferred to nitrocellulose membranes and probed with indicated antibodies as recommended by the manufacturer. Western blots were imaged using the ChemiDoc™ (Bio-Rad Laboratories, Hercules, CA, USA) for detection of horseradish peroxidase-conjugated secondary antibodies or the Odyssey® DLx Imaging System (a LICOR, Lincoln, NE, USA) for detection of near-infrared fluorescent-conjugated secondary antibodies. Phospho-ROS1 detection required the SuperSignal™ West Femto Maximum Sensitivity Substrate (Thermo Fisher Scientific). Densitometry was performed using Image Lab™ Software (RRID: SCR_014210).

Transient transfection assays to screen ROS1 TKD mutants

HEK-293T/17 cells were grown to 70% confluence in 12-well standard tissue culture dishes, and transfected with 2 μg pCX4 ROS1 wildtype or mutant variants using 8 μl TransIT-LT1 reagent. After 48 h, lysates prepared from transfected cells were harvested and an equal volume of 10 μl was loaded on 4–12% NuPAGE™ Bis-Tris protein gels. Methods for immunoblotting are described above. Relative ROS1 catalytic activity was determined using a ratio of densitometry data from phospho-ROS1 (pROS1) and total ROS1 (tROS1) antibody signals. Mutants showing higher pROS1/tROS1 ratio relative to wild-type were considered activating.

Cell proliferation and oncogenic transformation assays

MCF10A proliferation assay: Stable MCF10A cells expressing ROS1 variants were seeded in 96 well plates and treated with DMSO or different ROS1-TKIs ($n = 5$ per condition). For this assay, a modified growth medium was used where concentrations were reduced from 2 ng/ml to 0.01 or 0.02 ng/ml for EGF and 10% to 2% for HS, respectively. The final DMSO % was kept at ≤ 0.1%. For 96-well

© 2023 The Authors

plate experiments, 2,000 cells were seeded in a final working volume of 200 μl. Inhibitor treated cell proliferation experiments were done in 384 well plates; 200 cells per well were seeded and 20 h post seeding, indicated inhibitors were added to the wells using the HP300e Digital Dispenser. Cells were grown in these conditions for 5–7 days. Real-time proliferation was monitored using the Incucyte® ZOOM imaging platform and final cell confluence was measured via the CCK-8 (WST-8, tetrazolium)-based cell viability assay per manufacturer's protocol; 460 nm absorbance was read at 4 h post addition of CCK-8 reagent using a BioTek Synergy™ H1 plate reader. Data analysis and graph generation was using Microsoft Excel and GraphPad Prism v9.3 (GraphPad Software, RRID: SCR_002798, San Diego, CA, USA).

NIH-3T3 anchorage-independent soft agar growth assay: Anchorage-independent soft agar growth experiments were performed as described (Davare *et al*, 2013). Briefly, 8,000 cells (stable NIH-3T3 ROS1 variant cell lines) were seeded in a 0.2% top agarose layer layered on top of a 0.4% bottom agar layer. As indicated inhibitors were added in the feeding medium after top matrix solidified for 24 h. Every week, for upto 4 weeks, colonies were counted using the Gelcount™ colony counter (Oxford Optronix Ltd., Milton Park, Abingdon, UK). Resulting data were analyzed with Microsoft Excel (Microsoft Excel, RRID:SCR_016137) and GraphPad Prism.

Analysis of AACR Genie clinical sequencing data to find somatic ROS1 TKD mutations

AACR Genie data was queried for the ROS1 gene and then downloaded from genie.cbioportal.org (Cerami *et al*, 2012; Gao *et al*, 2013). All datasets on the website, including data from GENIE Cohort v11.0-public, DFCI-Profile Glioma Cohort 2013–2018, AACR Project GENIE AKT1 Cohort, and Metastatic Breast Cancer 2013–2016 datasets were included (Consortium TAPG, 2017; Touat *et al*, 2020; Garrido-Castro *et al*, 2021). The full list of ROS1 aberrations was narrowed down further using the 'Sorting Intolerant From Tolerant' (SIFT) scores (Sim *et al*, 2012) and limited to the kinase domain of ROS1.

Structural modeling and molecular dynamic simulation studies

The ROS1 kinase domain was modeled in the DFG-in and DFG-out conformations using YASARA Version 20.12.24 as we described previously for the NTRK kinase domain (Somwar *et al*, 2020). Point mutations were introduced, molecular dynamics simulations performed, and principal component analysis conducted using ProDy (Bakan *et al*, 2011; Bakan *et al*, 2014) with methods described in detail in our previous publication (Keddy *et al*, 2022).

Global proteomics and phosphoproteomics

Disulfide bond reduction/alkylation: Protein solutions (200 μg) were in 50 mM HEPES at 2 μg/μl in 1.5 ml Eppendorf low-bind tubes. Disulfide bonds within the proteins were reduced by adding tris (2-carboxyethyl) phosphine to a final concentration of 5 mM and mixing at room temperature for 15 min. The reduced proteins were alkylated by adding 2-chloroacetamide to a final concentration of 10 mm and mixing in the dark at room temperature for 30 min. Excess 2-chloroacetamide was quenched by adding dithiothreitol to a final concentration of 10 mM and mixing at room temperature for 15 min. Methanol/Chloroform precipitation and protease digestion: Alkylated samples were subjected to protein precipitation as follows: 400 μl of methanol was added to the sample and vortexed for 5, 100 μl of chloroform was added to the sample and vortexed for 5 s, 300 μl of water was added to the sample and vortexed for 5 s, and the samples were centrifuged for 1 min at 14,000 *g*. The aqueous and organic phases were removed, leaving a protein wafer in the tube. The protein wafers were washed with 400 μl of methanol and centrifuged at 21,000 *g* at room temperature for 2 min. The supernatants were removed, and the pellets were allowed to air dry but not to complete dryness. The samples were resuspended in 70 μl 100 mM HEPES (pH 8.5) and digested with rLys-C protease (100:1, protein to protease) with mixing at 37°C for 4 h. Trypsin protease (100:1, protein to protease) was added and the reaction was mixed overnight at 37°C. TMTpro15plex labeling: TMTpro16-plex labeling reagent (Pierce, 500 μg) was brought up in 30 μL acetonitrile and added to the digested peptide solution (200 μg) yielding a final organic concentration of 30% (v/v) and mixed at room temperature for 1 h. A 2 μg aliquot from each sample was combined, dried to remove the acetonitrile, processed with a C18 ZipTip (Millipore) and analyzed via LC/MS as a "label check". Equalization ratios and labeling efficiency were determined and the reactions were quenched with the addition of hydroxylamine to a final concentration of 0.3% (v/v) for 15 min with mixing. The TMTpro labeled samples were pooled at 1:1 ratio based on the equalization ratios from the label check and concentrated in a speedvac to remove acetonitrile. The material was desalted with a SepPak C18 3 ml cartridge (Waters) with the elution being split into two fractions, with 1 mg being used for pTyr enrichment and 2 mg being fractionated by basic reverse phase fractionation. Both fractions were taken to dryness using a SpeedVac. bRP Fractionation: The 2 mg combined TMT sample was resuspended in 100 μl 10 mM ammonium bicarbonate pH 8. The material was loaded on to a Zorbax 2.1 × 150 mm (5 μm particle size) Extend-C18 column (Agilent) for basic reverse-phase fractionation. The sample was gradient-eluted from the column at a flowrate of 250 μl/min over 55 min using a combination of solvents "A" (10 mM ammonium carbonate) and "B" (acetonitrile). The gradient used was as follows: from 0 to 5 min, "B" was held at 1%, from 5 to 55 min, "B" varied from 5% to 40%, followed by an increase to 90% "B" over 5 min, and then a hold for an additional 5 min at 90% "B". The UV signal was monitored at 210 nm. Ninety-six 50 s fractions were collected and combined into 24 pools by concatenation (where every 24th fraction was combined into a pool). The pools were subsequently taken to near-dryness by vacuum centrifugation. Pools were brought up to 100 μl in 98/2/0.1% (v:v) water:acetonitrile/formic acid and 5% from each pool was analyzed by LC–MS with an Orbitrap Eclipse. The pools were further combined to 12 fractions and taken to dryness by vacuum centrifugation for subsequent IMAC enrichment.

IMAC Enrichment: A 100 μl slurry of 5% Ni-NTA magnetic beads (Qiagen, part# 36113) was placed in 12 1.5 ml Eppendorf tubes and the tubes were placed in a magnetic stand and the supernatant was removed. The beads were rinsed three times with 100 μl water, each time placing the samples in the magnetic stand and removing the supernatant. A 100 μl solution of 40 mM EDTA was added to the beads, vortexed, mixed at ~ 1,400 rpm for 30 min at room

© 2023 The Authors

temperature, and the supernatant was removed. The beads were rinsed three times with 100 μl water, each time placing the sample on the magnetic stand and removing the supernatant. A 100 μl solution of 10 mM FeCl3 was added to the beads, vortexed and mixed at ~ 1,400 rpm for 30 min at room temperature, and the supernatant was removed. The beads were rinsed three times with 100 μl water, each time placing the sample on the magnet and removing the supernatant. The beads were rinsed three times with 100 μl 80% ACN/0.1% TFA before resuspending them in 100 μl 80% ACN/0.1% TFA. The bRP fractionated TMT labeled peptides (12 fractions) were resuspended in 200 μl 80% ACN/0.1% TFA and added to the resuspended beads. The samples were vortexed and mixed at ~ 1,400 rpm for 30 min at room temperature. Samples were spun down quickly at 1,000 *g* for 10 s and placed on the magnetic stand to remove the supernatant. The samples were washed three times with 300 μl 80% ACN/0.1% TFA, each time placing the samples on the magnetic stand and removing the supernatant. Phosphopeptide elution was carried out by adding 200 μl of 70% ACN/1% ammonium hydroxide to the beads, mixing at room temperature for 1 min, placing the samples on the magnetic stand, and transferring the supernatants into fresh Eppendorf tubes containing 60 μl of 10% formic acid and mixing. The samples were taken to dryness by vacuum centrifugation. Phosphotyrosine peptide enrichment: The 1 mg TMT-labeled peptide fraction underwent phosphotyrosine peptide enrichment using the PTMscan HS Phospho-Tyrosine (P-Tyr-1000) Kit (Cell Signaling Technology) following the manufacturer's instructions. Eluted peptides were desalted on an Ultra-micro Spin Column (Harvard Apparatus) and taken to dryness by vacuum centrifugation prior to mass spectrometry analysis. LC/MS: The generated basic reverse phase fractions were brought up in 2% acetonitrile in 0.1% formic acid (20 μl) and analyzed (2 μl, 18 μl IMAC and pY) by LC/ESI MS/MS with a Thermo Scientific Easy1200 nLC (Thermo Scientific, Waltham, MA) coupled to a tribrid Orbitrap Eclipse with FAIMS pro (Thermo Scientific, Waltham, MA) mass spectrometer. In-line de-salting was accomplished using a reversed-phase trap column (100 μm × 20 mm) packed with Magic C18AQ (5-μm 200 Å resin; Michrom Bioresources, Auburn, CA) followed by peptide separations on a reversed-phase column (75 μm × 270 mm) packed with ReproSil-Pur C18AQ (3-μm 120 Å resin; Dr. Maisch, Baden-Würtemburg, Germany) directly mounted on the electrospray ion source. A 120-min gradient from 4% to 44% B (80% acetonitrile in 0.1% formic acid/water) at a flow rate of 300 nl/min was used for chromatographic separations. A spray voltage of 2,300 V was applied to the electrospray tip in-line with a FAIMS pro source using varied compensation voltage −40, −60, −80 while the Orbitrap Eclipse instrument was operated in the data-dependent mode, MS survey scans were in the Orbitrap (Normalized AGC target value 300%, resolution 120,000, and max injection time 50 ms) with a 3 s cycle time and MS/MS spectra acquisition were detected in the Orbitrap (Normalized AGC target value of 250%, resolution 50,000 and max injection time 100 ms) using higher energy collision-induced dissociation (HCD) activation with HCD collision energy of 38%. Data analysis: Data analysis was performed using Proteome Discoverer 2.5 (Thermo Scientific, San Jose, CA). The data were searched against a Mouse database (UP00000598 Human 030721) that included common contaminants (cRAPome). Searches were performed with settings for the proteolytic enzyme trypsin. Maximum missed cleavages were set to 2. The precursor ion tolerance was set to 10 ppm and the fragment ion tolerance was set to 0.6 Da. Dynamic peptide modifications included oxidation (+15.995 Da on M). Dynamic modifications on the protein terminus included acetyl (+42.11 Da on N-terminus), Met-loss (−131.040 Da on M) and Met-loss+Acetyl (−89.030 Da on M) and static modifications TMTpro (+304.207 Da on any N-terminus), TMTpro (+304.207 DA on K) and carbamidomethyl (+57.021 on C). IMAC searches included phosphorylation (+79.966 Da on S, T, and Y) as a dynamic modification. Sequest HT was used for database searching. IMP-ptmRS was used for phospho searches. All search results were run through Percolator for scoring. Finally, downstream analysis using the KSEA and Causalpath software packages were run according to previously published protocols (Casado *et al*, 2013; Wiredja *et al*, 2017; Babur *et al*, 2021; Luna *et al*, 2021). Discoveries of significantly upregulated and downregulated proteins: Unpaired *t* tests of log2 transformed counts from ROS1 WT, ROS1 D2113N and SLC34A2-ROS1 cells were conducted using Graphpad Prism with variance assumption of individual variance for each role. Multiple Comparison tests used False Discovery Rate (FDR) with two-stage step-up (Benjamini, Krieger, and Yekutieli). Desired FDR was set to 5% for PTM data (IMAC and pTyr enrichment) and 10% for global peptide counts.

In vivo efficacy studies

All animal model studies were conducted in accordance with the Animal Welfare Act (AWA), Public Health Service (PHS), the United States Department of Agriculture (USDA), and under auspices of an approved protocol from the OHSU Institutional Animal Care and Use Committee (IACUC). Four-to eight-week-old female and male athymic nude mice (Nu/J, Strain # 002019, RRID: IMSR_JAX:002019) were obtained from The Jackson Laboratory (Bar Harbor, ME) and housed and handled under specific pathogen-free conditions in the University's Animal Care Facilities. After an initial two-week environmental adjustment period, mice were placed under anesthesia using 2% isoflurane/oxygen, weighed, and ear punched for identification purposes. Tumor cells (1–5 × 106 were mixed with 50 μl of matrigel and injected subcutaneously into the left or right flank. Animals were allowed to recover under supervision before being returned to animal facilities. Injected animals were checked daily until tumor were palpable nodules, at which time both animal weight and tumor size were measured thrice weekly using balance and a digital caliper (cat 14-648-17, Fisher Scientific, Federal Way, WA). Tumors were allowed to grow until they reached the humane limit of 1,500–2,000 mm^3 at which time animals were sacrificed and tumors were collected. At collection, tumors were washed in PBS and dissected with sterile scalpel blades and processed as follows: half the tumor was fixed in 10% normal formalin (24 h then placed in 70% EtOH) for immunohistochemistry, the rest was divided into aliquots for freezing in liquid nitrogen or tumor-derived cell line was generated (D2113N-P1). Initial studies of tumor formation were carried out in 4 female Nu/j mice (JAX lab) 4–6 weeks of age and repeated in male mice as shown in Fig EV5B.

Once tumor formation was documented, a follow-up study to test effect of TKI inhibitors on tumor growth was performed. ROS1-D2113N mutated NIH 3T3 cells were transduced with Firefly Luciferase lentiviral particles and selected with blasticidin (10 μg/ml)

according to the manufacturer protocol (Cellomics Technology, Rockville, MD). Using luciferase enabled monitoring of tumor growth using non-invasive bioluminescent imaging with the IVIS® Spectrum *in vivo* imaging system (Caliper LifeSciences, Hopkinton, MA). For this, luciferin dissolved in PBS was administered to mice by IP injection at a final dose of 150 mg/kg. After 6 min anesthesia was induced by 2.5% isoflurane/2.5% oxygen and mice were imaged with data being recorded. For the TKI treatments, 26 Nu/j mice (13 females/13 males) were injected with luciferase expressing ROS1-D2113N mutated NIH 3T3 cells (1 × 106) in the right flank after animals had been anesthetized with 2.5% isoflurane/2.5% oxygen. *In vivo* tumor measurement began 3 days after injection and caliper measurements were initiated as soon as palpable nodules were noted. TKI treatment began 29 days after initial injection when tumor volume was reliably ~ 90–120 mm^3). At this time, animals were randomly assigned to TKI treatment groups after photon measurements were ranked from high to low to ensure higher expressing tumors based on photon emission were randomly dispersed among lower expressing tumors. The study was not blinded. Control animals were treated with vehicle by gavage (ethanol/PEG200/Water 10%/40%/50%). The lorlatinib treatment group animals was 3 mg/kg lorlatinib daily via oral gavage (lorlatinib formulation was in ethanol/PEG200/Water 10/40/50). Crizotinib (100 mg/kg) treatment was also via oral gavage; crizotinib was formulated in 0.5% methyl cellulose/0.5% Tween-80. Mice were monitored for tumor growth by caliper measurements twice weekly and once a week assessed with the IVIS® Spectrum *in vivo* imaging system, until tumor volume approaches 2,000 mm^3, the humane end point of the study.

Statistical analysis

Mean ± SEM are shown unless otherwise stated. Student's *t*-test, one-way, or two-way ANOVA with multiple comparisons tests were used and specified in the figure legends. P values < 0.05 were deemed statistically significant. Either asterisks and-or P values are shown in figures and figure legends have corresponding detail on the methods used. All studies that have replicates report data on biological replicates (different individual biological samples from independent experiments e were used). For global proteomics and phosphoproteomics, both pairwise two-sample t tests and Wilcoxon rank-sum test were conducted (R package) to delineate differential expression proteins and Benjamini-Hochberg procedure was used for controlling the familywise error rate (FWER). All data were plotted and analyzed using GraphPad Prism v9.3 (RRID:SCR_002798).

Data availability

This study includes no data deposited in external repositories.

Expanded View for this article is available online.

Acknowledgements

This project was supported with funding from the National Institutes of Health (NRSA: F30CA247253 (Iyer), NIH/NCI R01CA233495 (Davare)), American Cancer Society (RSG-19-082-01-TBG (Davare)), OHSU Knight Cancer Institute (APEDI1366 (Iyer)), as well as the OHSU Doernbecher Children's Hospital Foundation. We also would like to acknowledge the American Association for Cancer Research and its financial and material support in the development of the AACR Project GENIE registry, as well as members of the consortium for their commitment to data sharing. Interpretations are the responsibility of study authors. We would also like to acknowledge the Fred Hutchinson Cancer Center Proteomics & Metabolomics core service, including Phil Gafken, Chenwei Lin, and Lisa Jones for their support in performing the global proteomics and phosphoproteomics experiments. The extensive Incucyte® experiments in this study were supported by the Advanced Light Microscopy Shared Resource at OHSU.

The paper explained

Problem

The functional consequences of numerous ROS1 mutations discovered in the cancer genome are not characterized and remain as variants of unknown significance, hindering understanding of their clinical relevance and potential for targeted therapies. In this study, we focused on investigating the impact of somatic missense mutations residing in the kinase domain of ROS1.

Results

The majority of studied ROS1 mutations have either neutral or deleterious impact on the catalytic function of the tyrosine kinase. However, a rare subset of mutations was activating. Among these, asparagine or glycine substitutions at the aspartate 2113 position in the activation loop of ROS1 robustly activated catalytic activity and promoted ROS1 TKI-sensitive oncogenic transformation and tumor formation *in vivo*.

Impact

The identification of gain-of-function mutations in ROS1 and the corresponding therapeutic response to existing FDA-approved ROS1 TKIs offer potential personalized treatment strategies for ROS1-altered cancers.

Author contributions

Sudarshan R Iyer: Data curation; formal analysis; funding acquisition; investigation; methodology; writing – original draft; writing – review and editing. **Kevin Nusser:** Investigation; methodology. **Kristen Jones:** Investigation; methodology. **Clare Keddy:** Data curation; formal analysis; validation; investigation; methodology. **Pushkar Shinde:** Formal analysis; investigation; methodology; writing – original draft; writing – review and editing. **Catherine Z Beach:** Investigation. **Erin Aguero:** Methodology. **Jeremy Force:** Investigation; methodology; writing – review and editing. **Ujwal Shinde:** Investigation; methodology; writing – original draft; writing – review and editing. **Monika A Davare:** Conceptualization; data curation; formal analysis; supervision; funding acquisition; validation; investigation; methodology; writing – original draft; project administration; writing – review and editing.

Disclosure and competing interests statement

MAD received research funding from Nuvalent, Inc.

For more information

1 https://www.cbioportal.org/
2 https://biit.cs.ut.ee/gprofiler/gost

© 2023 The Authors

References

Arai Y, Totoki Y, Takahashi H, Nakamura H, Hama N, Kohno T, Tsuta K, Yoshida A, Asamura H, Mutoh M *et al* (2013) Mouse model for ROS1-rearranged lung cancer. *PLoS One* 8: e56010

Babur O, Luna A, Korkut A, Durupinar F, Siper MC, Dogrusoz U, Vaca Jacome AS, Peckner R, Christianson KE, Jaffe JD *et al* (2021) Causal interactions from proteomic profiles: Molecular data meet pathway knowledge. *Patterns* 2: 100257

Bakan A, Dutta A, Mao W, Liu Y, Chennubhotla C, Lezon TR, Bahar I (2014) Evol and ProDy for bridging protein sequence evolution and structural dynamics. *Bioinformatics* 30: 2681 – 2683

Bakan A, Meireles LM, Bahar I (2011) ProDy: protein dynamics inferred from theory and experiments. *Bioinformatics* 27: 1575 – 1577

Borowicz S, Scoyk MV, Avasarala S, Rathinam MKK, Tauler J, Bikkavilli RK, Winn RA (2014) The soft agar colony formation assay. *J Vis Exp* e51998 https://doi.org/10.3791/51998

Bresler Scott C, Weiser Daniel A, Huwe Peter J, Park Jin H, Krytska K, Ryles H, Laudenslager M, Rappaport Eric F, Wood Andrew C, McGrady Patrick W *et al* (2014) ALK mutations confer differential oncogenic activation and sensitivity to ALK inhibition therapy in neuroblastoma. *Cancer Cell* 26: 682 – 694

Brewer MR, Yun C-H, Lai D, Lemmon MA, Eck MJ, Pao W (2013) Mechanism for activation of mutated epidermal growth factor receptors in lung cancer. *Proc Natl Acad Sci USA* 110: E3595 – E3604

Casado P, Rodriguez-Prados J-C, Cosulich SC, Guichard S, Vanhaesebroeck B, Joel S, Cutillas PR (2013) Kinase-substrate enrichment analysis provides insights into the heterogeneity of signaling pathway activation in leukemia cells. *Sci Signal* 6: rs6

Cerami E, Gao J, Dogrusoz U, Gross BE, Sumer SO, Aksoy BA, Jacobsen A, Byrne CJ, Heuer ML, Larsson E *et al* (2012) The cBio cancer genomics portal: an open platform for exploring multidimensional cancer genomics data. *Cancer Discov* 2: 401 – 404

Cocco E, Scaltriti M, Drilon A (2018) NTRK fusion-positive cancers and TRK inhibitor therapy. *Nat Rev Clin Oncol* 15: 731 – 747

Consortium TAPG (2017) AACR Project GENIE: powering precision medicine through an international consortium. *Cancer Discov* 7: 818 – 831

Davare MA, Saborowski A, Eide CA, Tognon C, Smith RL, Elferich J, Agarwal A, Tyner JW, Shinde UP, Lowe SW *et al* (2013) Foretinib is a potent inhibitor of oncogenic ROS1 fusion proteins. *Proc Natl Acad Sci USA* 110: 19519 – 19524

Davare MA, Vellore NA, Wagner JP, Eide CA, Goodman JR, Drilon A, Deininger MW, O'Hare T, Druker BJ (2015) Structural insight into selectivity and resistance profiles of ROS1 tyrosine kinase inhibitors. *Proc Natl Acad Sci USA* 112: E5381 – E5390

Davare MA, Henderson JJ, Agarwal A, Wagner JP, Iyer SR, Shah N, Woltjer R, Somwar R, Gilheeney SW, deCarvalho AC *et al* (2018) Rare but recurrent ROS1 fusions resulting from chromosome 6q22 microdeletions are targetable oncogenes in glioma. *Clin Cancer Res* 24: 6471 – 6482

Davies KD, Le AT, Theodoro MF, Skokan MC, Aisner DL, Berge EM, Terracciano LM, Cappuzzo F, Incarbone M, Roncalli M *et al* (2012) Identifying and targeting ROS1 gene fusions in non–small cell lung cancer. *Am Assoc Cancer Res* 18: 4570 – 4579

Debnath J, Muthuswamy SK, Brugge JS (2003) Morphogenesis and oncogenesis of MCF-10A mammary epithelial acini grown in three-dimensional basement membrane cultures. *Methods* 30: 256 – 268

Derynck R, Turley SJ, Akhurst RJ (2021) TGFβ biology in cancer progression and immunotherapy. *Nat Rev Clin Oncol* 18: 9 – 34

Drilon A, Cappuzzo F, Ou S-HI, Camidge DR (2017a) Targeting MET in lung cancer: will expectations finally be MET? *J Thorac Oncol* 12: 15 – 26

Drilon A, Siena S, Ou SI, Patel M, Ahn MJ, Lee J, Bauer TM, Farago AF, Wheler JJ, Liu SV *et al* (2017b) Safety and antitumor activity of the multitargeted Pan-TRK, ROS1, and ALK inhibitor entrectinib: combined results from two phase I trials (ALKA-372-001 and STARTRK-1). *Cancer Discov* 7: 400 – 409

Drilon A, Siena S, Dziadziuszko R, Barlesi F, Krebs MG, Shaw AT, Braud F, Rolfo C, Ahn M-J, Wolf J *et al* (2020) Entrectinib in ROS1 fusion-positive non-small-cell lung cancer: integrated analysis of three phase 1–2 trials. *Lancet Oncol* 21: 261 – 270

Drilon A, Jenkins C, Iyer S, Schoenfeld A, Keddy C, Davare MA (2021) ROS1-dependent cancers – biology, diagnostics and therapeutics. *Nat Rev Clin Oncol* 18: 35 – 55

Drilon A, Horan JC, Tangpeerachaikul A, Besse B, Ou SI, Gadgeel SM, Camidge DR, van der Wekken AJ, Nguyen-Phuong L, Acker A *et al* (2023) NVL-520 is a selective, TRK-sparing, and brain-penetrant inhibitor of ROS1 fusions and secondary resistance mutations. *Cancer Discov* 13: 598 – 615

Du Z, Lovly CM (2018) Mechanisms of receptor tyrosine kinase activation in cancer. *Mol Cancer* 17: 58

Fleuren EDG, Zhang L, Wu J, Daly RJ (2016) The kinome 'at large' in cancer. *Nat Rev Cancer* 16: 83 – 98

Gao J, Aksoy BA, Dogrusoz U, Dresdner G, Gross B, Sumer SO, Sun Y, Jacobsen A, Sinha R, Larsson E *et al* (2013) Integrative analysis of complex cancer genomics and clinical profiles using the cBioPortal. *Sci Signal* 6: pl1

Garrido-Castro AC, Spurr LF, Hughes ME, Li YY, Cherniack AD, Kumari P, Lloyd MR, Bychkovsky B, Barroso-Sousa R, Di Lascio S *et al* (2021) Genomic characterization of *de novo* metastatic breast cancer. *Clin Cancer Res* 27: 1105 – 1118

George RE, Sanda T, Hanna M, Fröhling S, Ii WL, Zhang J, Ahn Y, Zhou W, London WB, McGrady P *et al* (2008) Activating mutations in ALK provide a therapeutic target in neuroblastoma. *Nature* 455: 975 – 978

Gillespie M, Jassal B, Stephan R, Milacic M, Rothfels K, Senff-Ribeiro A, Griss J, Sevilla C, Matthews L, Gong C *et al* (2022) The reactome pathway knowledgebase 2022. *Nucleic Acids Res* 50: D687 – D692

Hanahan D (2022) Hallmarks of cancer: new dimensions. *Cancer Discov* 12: 31 – 46

Hanahan D, Weinberg Robert A (2011) Hallmarks of cancer: the next generation. *Cell* 144: 646 – 674

Haque I, Mehta S, Majumder M, Dhar K, De A, McGregor D, Veldhuizen PJV, Banerjee SK, Banerjee S (2011) Cyr61/CCN1 signaling is critical for epithelial-mesenchymal transition and stemness and promotes pancreatic carcinogenesis. *Mol Cancer* 10: 8

Horn H, Schoof EM, Kim J, Robin X, Miller ML, Diella F, Palma A, Cesareni G, Jensen LJ, Linding R (2014) KinomeXplorer: an integrated platform for kinome biology studies. *Nat Methods* 11: 603 – 604

Hornbeck PV, Zhang B, Murray B, Kornhauser JM, Latham V, Skrzypek E (2014) PhosphoSitePlus, 2014: mutations, PTMs and recalibrations. *Nucleic Acids Res* 43: D512 – D520

Inoue M, Toki H, Matsui J, Togashi Y, Dobashi A, Fukumura R, Gondo Y, Minowa O, Tanaka N, Mori S *et al* (2016) Mouse models for ROS1 -fusion-positive lung cancers and their application to the analysis of multikinase inhibitor efficiency. *Carcinogenesis* 37: 452 – 460

Ip CKM, Ng PKS, Jeong KJ, Shao SH, Ju Z, Leonard PG, Hua X, Vellano CP, Woessner R, Sahni N *et al* (2018) Neomorphic PDGFRA extracellular domain driver mutations are resistant to PDGFRA targeted therapies. *Nat Commun* 9: 4583

Isakoff SJ, Engelman JA, Irie HY, Luo J, Brachmann SM, Pearline RV, Cantley LC, Brugge JS (2005) Breast cancer–associated PIK3CA mutations are oncogenic in mammary epithelial cells. *Cancer Res* 65: 10992 – 11000

© 2023 The Authors

Keddy C, Shinde P, Jones K, Kaech S, Somwar R, Shinde U, Davare MA (2022) Resistance profile and structural modeling of next-generation ROS1 tyrosine kinase inhibitors. *Mol Cancer Ther* 21: 336 – 346

Kim H, Son S, Shin I (2018) Role of the CCN protein family in cancer. *BMB Rep* 51: 486 – 492

Lahiry P, Torkamani A, Schork NJ, Hegele RA (2010) Kinase mutations in human disease: interpreting genotype–phenotype relationships. *Nat Rev Genet* 11: 60 – 74

Landi L, Chiari R, Tiseo M, D'Inca F, Dazzi C, Chella A, Delmonte A, Bonanno L, Giannarelli D, Cortinovis DL *et al* (2019) Crizotinib in MET-deregulated or ROS1-rearranged pretreated non-small cell lung cancer (METROS): a phase II, prospective, multicenter, two-arms trial. *Clin Cancer Res* 25: 7312 – 7319

Lau LF (2012) CCN1 and CCN2: blood brothers in angiogenic action. *J Cell Commun Signal* 6: 121 – 123

Lemmon MA, Schlessinger J (2010) Cell signaling by receptor tyrosine kinases. *Cell* 141: 1117 – 1134

Lin JJ, Shaw AT (2017) Recent advances in targeting ROS1 in lung cancer. *J Thorac Oncol* 12: 1611 – 1625

Luna A, Siper MC, Korkut A, Durupinar F, Dogrusoz U, Aslan JE, Sander C, Demir E, Babur O (2021) Analyzing causal relationships in proteomic profiles using CausalPath. *STAR Protoc* 2: 100955

Martínez L (2015) Automatic identification of mobile and rigid substructures in molecular dynamics simulations and fractional structural fluctuation analysis. *PLoS One* 10: e0119264

Massagué J (2008) TGFβ in cancer. *Cell* 134: 215 – 230

Medves S, Demoulin JB (2012) Tyrosine kinase gene fusions in cancer: translating mechanisms into targeted therapies. *J Cell Mol Med* 16: 237 – 248

Modi V, Dunbrack RL (2019) Defining a new nomenclature for the structures of active and inactive kinases. *Proc Natl Acad Sci USA* 116: 6818 – 6827

Mossé YP (2016) Anaplastic lymphoma kinase as a cancer target in pediatric malignancies. *Clin Cancer Res* 22: 546 – 552

Nakerakanti SS, Bujor AM, Trojanowska M (2011) CCN2 is required for the TGF-β induced activation of Smad1 – Erk1/2 signaling network. *PLoS One* 6: e21911

Neel DS, Allegakoen DV, Olivas V, Mayekar MK, Hemmati G, Chatterjee N, Blakely CM, McCoach CE, Rotow JK, Le A *et al* (2019) Differential subcellular localization regulates oncogenic signaling by ROS1 kinase fusion proteins. *Cancer Res* 79: 546 – 556

Ni J, Yang Y, Wang Q, Bergholz JS, Jiang T, Roberts TM, Gray NS, Zhao JJ (2022) Targeting EGFR in glioblastoma with a novel brain-penetrant small molecule EGFR-TKI. *bioRxiv* https://doi.org/10.1101/2021.01.09.426030 [PREPRINT]

Paik S, Kim C, Wolmark N (2008) HER2 status and benefit from adjuvant trastuzumab in breast cancer. *N Engl J Med* 358: 1409 – 1411

Raudvere U, Kolberg L, Kuzmin I, Arak T, Adler P, Peterson H, Vilo J (2019) g: Profiler: a web server for functional enrichment analysis and conversions of gene lists (2019 update). *Nucleic Acids Res* 47: W191 – W198

Reva B, Antipin Y, Sander C (2007) Determinants of protein function revealed by combinatorial entropy optimization. *Genome Biol* 8: R232

Reva B, Antipin Y, Sander C (2011) Predicting the functional impact of protein mutations: application to cancer genomics. *Nucleic Acids Res* 39: e118

Rikova K, Guo A, Zeng Q, Possemato A, Yu J, Haack H, Nardone J, Lee K, Reeves C, Li Y *et al* (2007) Global survey of phosphotyrosine signaling identifies oncogenic kinases in lung cancer. *Cell* 131: 1190 – 1203

Saborowski A, Saborowski M, Davare MA, Druker BJ, Klimstra DS, Lowe SW (2013) Mouse model of intrahepatic cholangiocarcinoma validates FIG–ROS as a potent fusion oncogene and therapeutic target. *Proc Natl Acad Sci USA* 110: 19513 – 19518

Sanchez-Vega F, Mina M, Armenia J, Chatila WK, Luna A, La KC, Dimitriadoy S, Liu DL, Kantheti HS, Saghafinia S *et al* (2018) Oncogenic signaling pathways in The Cancer Genome Atlas. *Cell* 173: 321 – 337.e10

Schlessinger J (2014) Receptor tyrosine kinases: legacy of the first two decades. *Cold Spring Harb Perspect Biol* 6: a008912

Schram AM, Chang MT, Jonsson P, Drilon A (2017) Fusions in solid tumours: diagnostic strategies, targeted therapy, and acquired resistance. *Nat Rev Clin Oncol* 14: 735 – 748

Sharma SV, Bell DW, Settleman J, Haber DA (2007) Epidermal growth factor receptor mutations in lung cancer. *Nat Rev Cancer* 7: 169 – 181

Shaw AT, Ou S-HI, Bang Y-J, Camidge RD, Solomon BJ, Salgia R, Riely GJ, Varella-Garcia M, Shapiro GI, Costa DB *et al* (2014) Crizotinib in ROS1-rearranged non–small-cell lung cancer. *N Engl J Med* 371: 1963 – 1971

Sim N-L, Kumar P, Hu J, Henikoff S, Schneider G, Ng PC (2012) SIFT web server: predicting effects of amino acid substitutions on proteins. *Nucleic Acids Res* 40: W452 – W457

Smith JS, Tachibana I, Passe SM, Huntley BK, Borell TJ, Iturria N, O'Fallon JR, Schaefer PL, Scheithauer BW, James CD *et al* (2001) PTEN mutation, EGFR amplification, and outcome in patients with anaplastic astrocytoma and glioblastoma multiforme. *J Natl Cancer Inst* 93: 1246 – 1256

Somwar R, Hofmann NE, Smith B, Odintsov I, Vojnic M, Linkov I, Tam A, Khodos I, Mattar MS, de Stanchina E *et al* (2020) NTRK kinase domain mutations in cancer variably impact sensitivity to type I and type II inhibitors. *Commun Biol* 3: 776

Tejera-Muñoz A, Marquez-Exposito L, Tejedor-Santamaría L, Rayego-Mateos S, Orejudo M, Suarez-Álvarez B, López-Larrea C, Ruíz-Ortega M, Rodrigues-Díez RR (2021) CCN2 increases TGF-β receptor type II expression in vascular smooth muscle cells: essential role of CCN2 in the TGF-β pathway regulation. *Int J Mol Sci* 23: 375

Thusberg J, Vihinen M (2009) Pathogenic or not? And if so, then how? Studying the effects of missense mutations using bioinformatics methods. *Hum Mutat* 30: 703 – 714

Touat M, Li YY, Boynton AN, Spurr LF, Iorgulescu JB, Bohrson CL, Cortes-Ciriano I, Birzu C, Geduldig JE, Pelton K *et al* (2020) Mechanisms and therapeutic implications of hypermutation in gliomas. *Nature* 580: 517 – 523

Vaser R, Adusumalli S, Leng SN, Sikic M, Ng PC (2016) SIFT missense predictions for genomes. *Nat Protoc* 11: 1 – 9

Wiredja DD, Koyutürk M, Chance MR (2017) The KSEA App: a web-based tool for kinase activity inference from quantitative phosphoproteomics. *Bioinformatics* 33: 3489 – 3491

Zou HY, Friboulet L, Kodack DP, Engstrom LD, Li Q, West M, Tang RW, Wang H, Tsaparikos K, Wang J *et al* (2015) PF-06463922, an ALK/ROS1 inhibitor, overcomes resistance to first and second generation ALK inhibitors in preclinical models. *Cancer Cell* 28: 70 – 81

License: This is an open access article under the terms of the Creative Commons Attribution License, which permits use, distribution and reproduction in any medium, provided the original work is properly cited.

© 2023 The Authors

Expanded View Figures

A

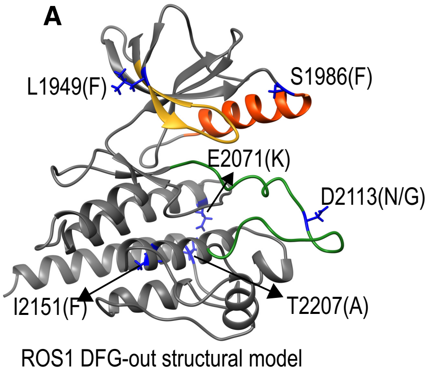

B

Mutation	Region of Kinase Domain	Tumor Type	Frequency of Somatic Mutations
**L1949F**	P-Loop	Melanoma	1/5402 (0.02%)
**S1986F**	αC-Helix	Glioma	1/9061 (0.01%)
		Melanoma	1/5402 (0.02%)
		Ovarian Cancer	1/5428 (0.02%)
**E2071K**	αE-Helix	Breast Cancer	1/856 (0.12%)
		Glioma	1/9061 (0.01%)
		Melanoma	5/5402 (0.06%)
**D2113G**	A-Loop	Esophageal Adenocarcinoma	1/53 (1.9%)
**D2113N**	A-Loop	Glioma	1/9061 (0.01%)
		Melanoma	5/5402 (0.1%)

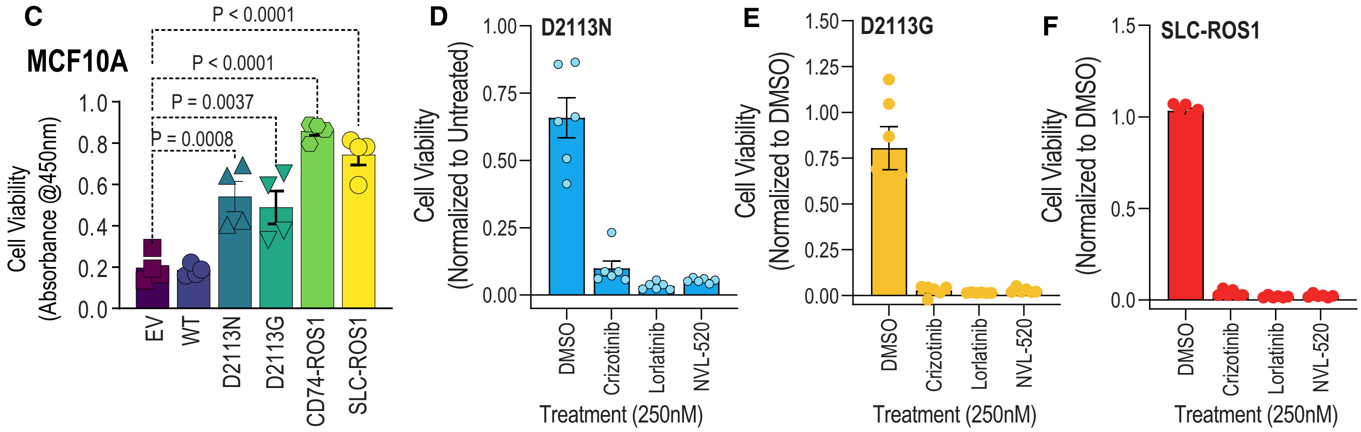

Figure EV1. EGF-independent growth of MCF10A ROS1 D2113N, D2113G or SLC34A2-ROS1 fusions is sensitivity to ROS1 TKI.

A Ribbon diagram of ROS1 kinase domain structural model annotated with amino acid substitutions that increased catalytic activity.

B Frequency of activating ROS1 alterations in cancer.

C Colorimetric cell viability data after growth of MCF10A cells expressing Empty Vector (EV), ROS1 wildtype (WT), $ROS1^{D2113N}$, $ROS1^{D2113G}$, CD74-ROS1 fusion and SLC34A2-ROS1 (SLC-ROS1) fusion in low EGF medium for 108 h (N = 4, biological replicates). CCK-8 (water-soluble tetrazolium salt, WST-8) reagent was added to the 96-well plates after completion of real-time imaging in the Incucyte® live imaging platform.

D–F Cell viability of MCF10A $ROS1^{D2113N}$ (D), D2113G (E) and SLC-ROS1 (F) in low EGF medium with or without ROS1 TKI, crizotinib, lorlatinib, and NVL-520 (250 nM in all cases) for 108 h, measured using CCK-8 colorimetric reagent (N = 6, biological replicates). One-way ANOVA with Dunnett's multiple comparisons test was used with alpha of 0.05. Error bars in figure represent mean ± SEM.

Source data are available online for this figure.

© 2023 The Authors

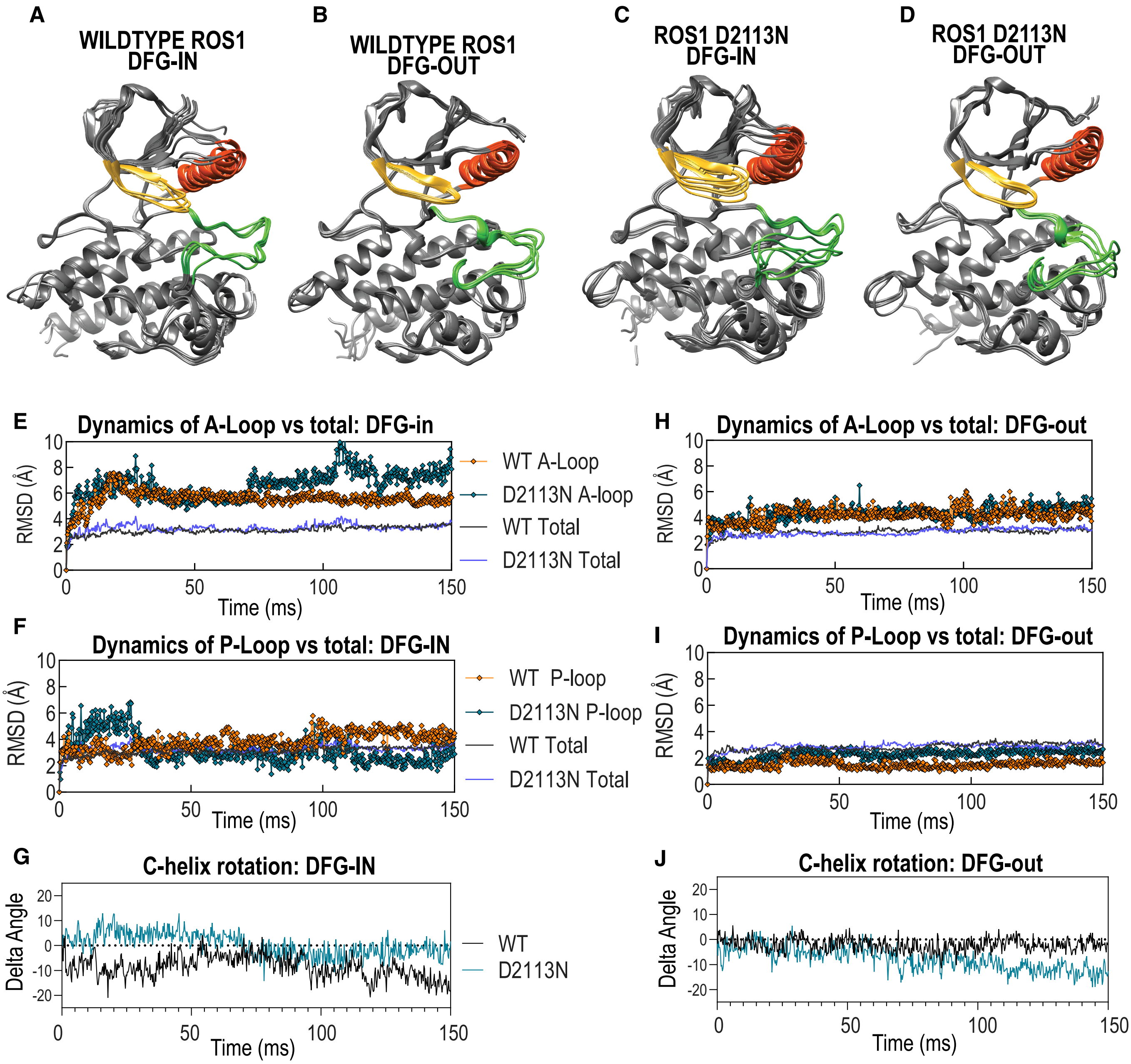

Figure EV2. Molecular dynamic simulations of ROS1^{WT} and ROS1^{D2113N} kinase demonstrate effects on A-loop in the DFG-in conformation but minimal impact on other domains.

A–J (A–D) Six individual representative poses adopted by ROS1^{WT} and ROS1^{D2113N} in DFG-in and DFG-out conformations as indicated by labels. Root Mean Square Deviation values (*Y*-axis) plotted over 150 ms of simulation duration (*X*-axis) for A-loop in DFG-in conformation (E), A-loop DFG-out conformation (H), P-loop in DFG-in (F) and DFG-out (I) conformations and αC-helix in DFG-in (G) and DFG-out (J) conformations.

 © 2023 The Authors

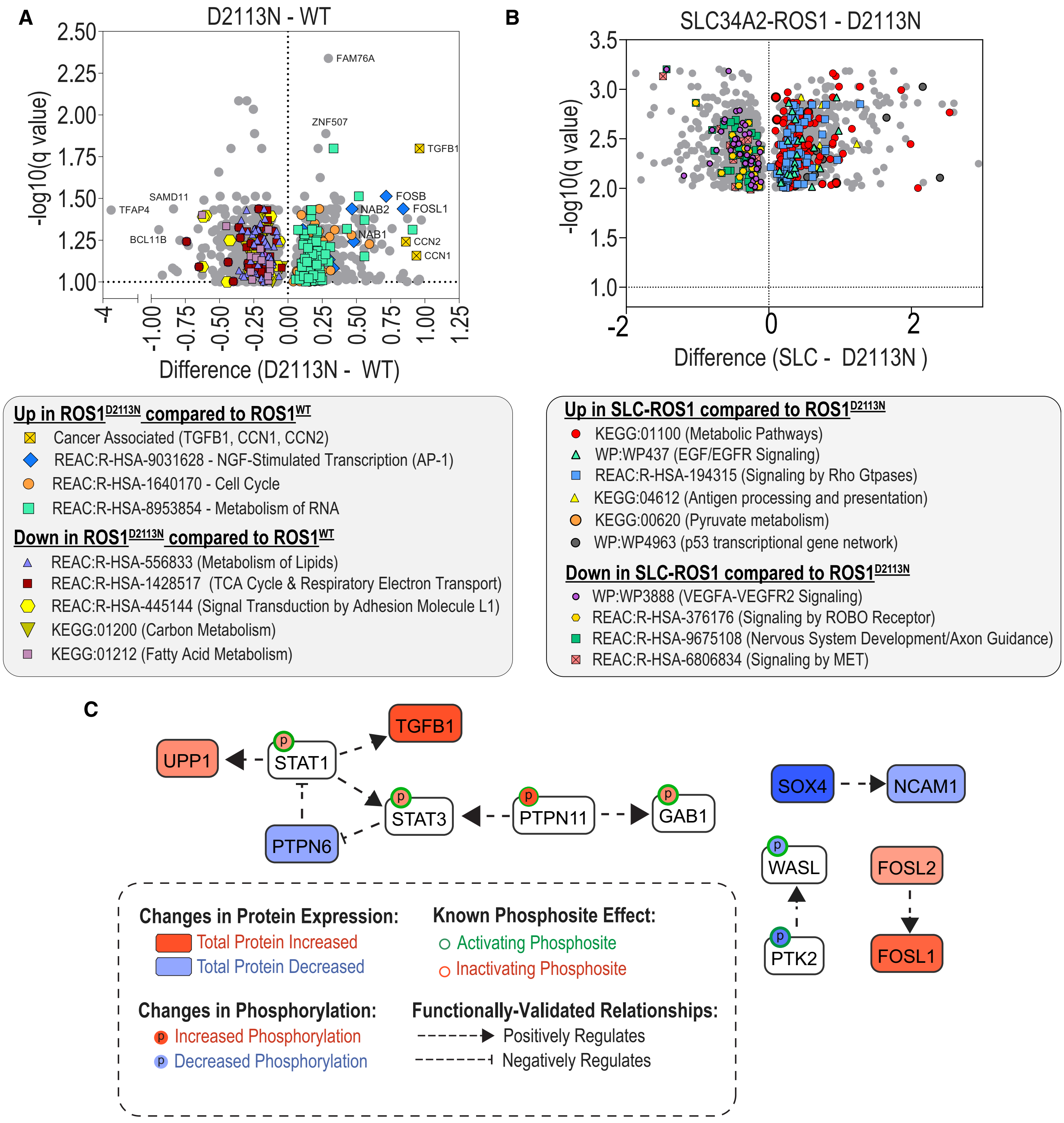
A
D2113N - WT
-log10(q value)
Difference (D2113N - WT)
FAM76A
ZNF507
TGFB1
SAMD11
TFAP4
BCL11B
FOSB
NAB2
FOSL1
NAB1
CCN2
CCN1
B
SLC34A2-ROS1 - D2113N
-log10(q value)
Difference (SLC - D2113N)
Up in ROS1D2113N compared to ROS1WT
Cancer Associated (TGFB1, CCN1, CCN2)
REAC:R-HSA-9031628 - NGF-Stimulated Transcription (AP-1)
REAC:R-HSA-1640170 - Cell Cycle
REAC:R-HSA-8953854 - Metabolism of RNA
Down in ROS1D2113N compared to ROS1WT
REAC:R-HSA-556833 (Metabolism of Lipids)
REAC:R-HSA-1428517 (TCA Cycle & Respiratory Electron Transport)
REAC:R-HSA-445144 (Signal Transduction by Adhesion Molecule L1)
KEGG:01200 (Carbon Metabolism)
KEGG:01212 (Fatty Acid Metabolism)
Up in SLC-ROS1 compared to ROS1D2113N
KEGG:01100 (Metabolic Pathways)
WP:WP437 (EGF/EGFR Signaling)
REAC:R-HSA-194315 (Signaling by Rho Gtpases)
KEGG:04612 (Antigen processing and presentation)
KEGG:00620 (Pyruvate metabolism)
WP:WP4963 (p53 transcriptional gene network)
Down in SLC-ROS1 compared to ROS1D2113N
WP:WP3888 (VEGFA-VEGFR2 Signaling)
REAC:R-HSA-376176 (Signaling by ROBO Receptor)
REAC:R-HSA-9675108 (Nervous System Development/Axon Guidance)
REAC:R-HSA-6806834 (Signaling by MET)
C
UPP1
STAT1
TGFB1
STAT3
PTPN6
PTPN11
GAB1
SOX4
NCAM1
WASL
PTK2
FOSL2
FOSL1
Changes in Protein Expression:
Total Protein Increased
Total Protein Decreased
Known Phosphosite Effect:
Activating Phosphosite
Inactivating Phosphosite
Changes in Phosphorylation:
Increased Phosphorylation
Decreased Phosphorylation
Functionally-Validated Relationships:
Positively Regulates
Negatively Regulates

Figure EV3.

◀ **Figure EV3. Global proteomics and causal path analysis for ROS1^{D2113N}.**

A Volcano plots showing differential expression of proteins in HEK293A ROS1^{D2113N} cells compared to HEK293A ROS1^{WT} cells (N = 3, biological replicates). Multiple unpaired *t*-test analysis was performed on $\log_2$ transformed ratio of normalized intensities (D2113N—WT). Multiple comparisons were via false discovery rate (FDR) with Desired FDR Q = 10%. Two-stage step-up Benjamini, Krieger, and Yekutieli method was used for the false discovery rate approach. The *X*-axis features significant discoveries of ROS1^{D2113N} relative to ROS1^{WT} (D2113N—WT) and *Y*-axis features corresponding log10(q) values (shown as –log10(q)). G:Profiler: a web server for functional enrichment analysis was used to curate significantly upregulated or downregulated pathways associated with KEGG, Reactome or Wikipathways databases (Raudvere *et al*, 2019) and indicated in annotated box below.

B Volcano plots showing differential expression of proteins in HEK293A SLC-ROS1 compared to HEK293A ROS1^{D2113N} cells (N = 3, biological replicates). All analysis parameters are identical to described above in panel (A) with the exception of the Desired FDR Q = 1% in the SLC-ROS1—D2113N analysis.

C Causalpath analysis of global proteomics and phosphoproteomics log2-transformed spectral counts highlights signaling changes promoted by ROS1^{D2113N} relative to ROS1^{WT}. Source data is located in Dataset EV2.

 © 2023 The Authors

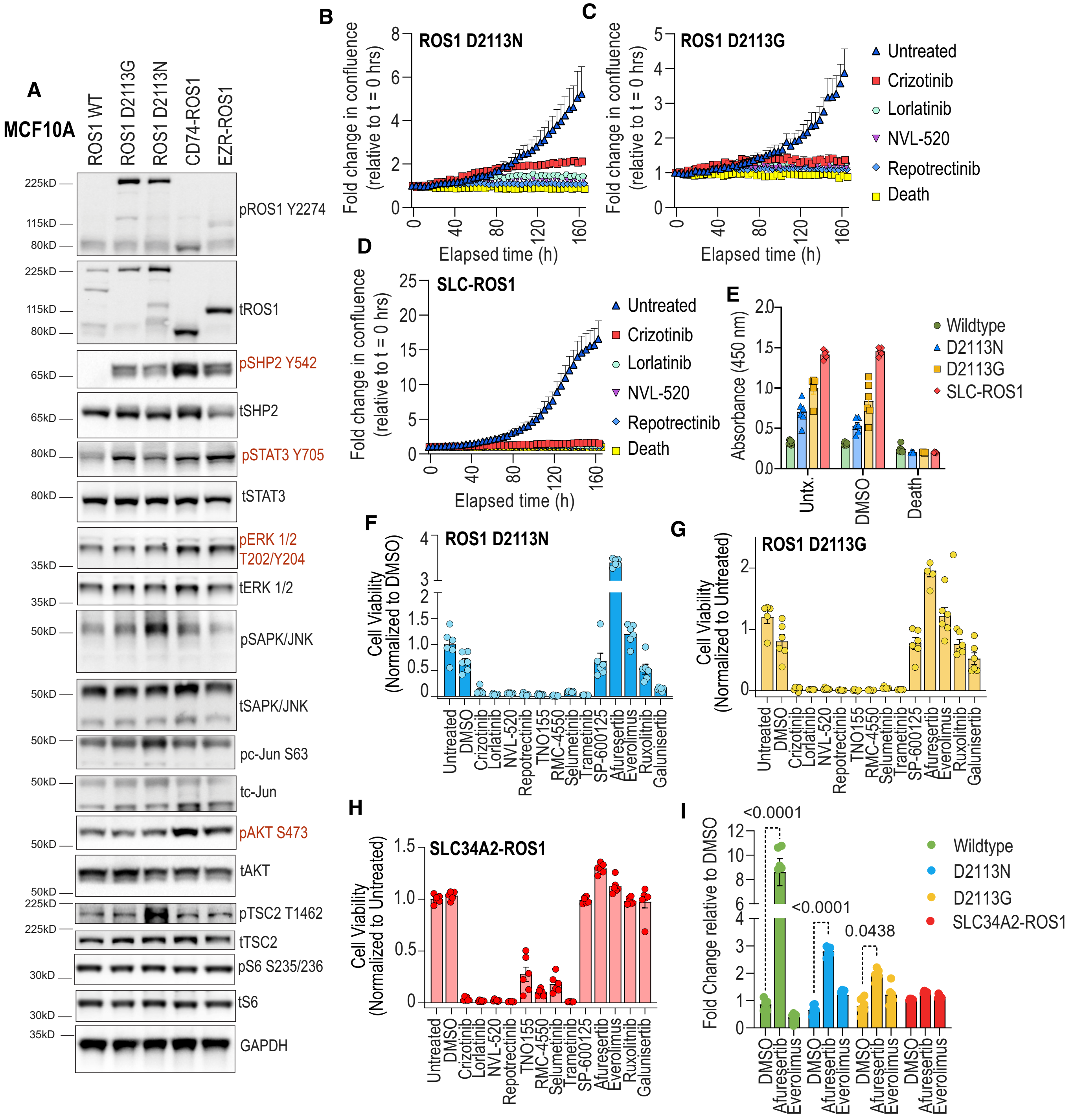
A
MCF10A
ROS1 WT
ROS1 D2113G
ROS1 D2113N
CD74-ROS1
EZR-ROS1
225kD
115kD
80kD
65kD
35kD
50kD
30kD
pROS1 Y2274
tROS1
pSHP2 Y542
tSHP2
pSTAT3 Y705
tSTAT3
pERK 1/2 T202/Y204
tERK 1/2
pSAPK/JNK
tSAPK/JNK
pc-Jun S63
tc-Jun
pAKT S473
tAKT
pTSC2 T1462
tTSC2
pS6 S235/236
tS6
GAPDH
B
ROS1 D2113N
C
ROS1 D2113G
Fold change in confluence (relative to t = 0 hrs)
Elapsed time (h)
Untreated
Crizotinib
Lorlatinib
NVL-520
Repotrectinib
Death
D
SLC-ROS1
E
Absorbance (450 nm)
Wildtype
D2113N
D2113G
SLC-ROS1
Untx.
DMSO
Death
F
ROS1 D2113N
Cell Viability (Normalized to DMSO)
G
ROS1 D2113G
Cell Viability (Normalized to Untreated)
Untreated
DMSO
Crizotinib
Lorlatinib
NVL-520
Repotrectinib
TNO155
RMC-4550
Selumetinib
Trametinib
SP-600125
Afuresertib
Everolimus
Ruxolitinib
Galunisertib
H
SLC34A2-ROS1
Cell Viability (Normalized to Untreated)
I
Fold Change relative to DMSO
<0.0001
<0.0001
0.0438
Wildtype
D2113N
D2113G
SLC34A2-ROS1
DMSO
Afuresertib
Everolimus

Figure EV4.

Figure EV4. Regulation of signaling pathways in MCF10A ROS1 variant expressing cells.

A Immunoblots performed on cell lysates generated from stable MCF10A cell lines expressing ROS1^{WT}, ROS1^{D2113N}, ROS1^{D2113G}, CD74-ROS1 and EZR-ROS1 fusion proteins. Phosphosite specific and total antibodies used are indicated in the blot labels. Following target proteins were interrogated: ROS1, SHP2, STAT3, ERK1/2, JNK/SAPK, c-Jun, AKT1, TSC2, S6, and GAPDH (Loading control). Red colored western blot labels indicate pathways that are upregulated either in ROS1 point mutations, ROS1 fusions or both. Prior to cell lysis, EGF withdrawal was done for 4 h.

B–D Inhibition of cell proliferation in ROS1 TKI treated MCF10A ROS1^{D2113N} (B), ROS1^{D2113G} (C) and SLC34A2-ROS1 (D) cell lines (N = 6, biological replicates). Cells were treated with 250 nM crizotinib, lorlatinib, NVL-520 and repotrectinib for the duration of the live-imaging experiment. “Death” indicates a cocktail of 10 μM staurosporine plus YM155 inhibitors that induce complete death and serve a positive control for cell death in live imaging and colorimetric cell viability assays.

E Colorimetric cell viability data from MCF10A ROS1^{WT}, ROS1^{D2113N}, ROS1^{D2113G}, and SLC34A2-ROS1 cell lines that were untreated, treated with DMSO, or “Death” cocktail as explained previously (N = 6, biological replicates).

F–H Colorimetric cell viability data from ROS1^{D2113N} (F), ROS1^{D2113G} (G), and SLC34A2-ROS1 (H) cell lines treated with the indicated signaling effector inhibitors (N = 6, biological replicates). 250 nM treatment with DMSO, crizotinib, lorlatinib, NVL-520, repotrectinib, TNO155, RMC-4550, selumetinib, trametinib, afuresertib, everolimus, SP-600125, and ruxolitinib. 500 nM treatment with galusertinib.

I Colorimetric cell viability data from MCF10A ROS1^{WT}, ROS1^{D2113N}, ROS1^{D2113G}, and SLC34A2-ROS1 treated with DMSO, 250 nM afuresertib, or 250 nM everolimus. P values from multiple comparison tests displayed in chart. Cell viability measurements displayed in figure were either raw absorbance of reduced tetrazolium salt, WST-8 (CCK-8), measured at 460 nm, or fold change of raw absorbance relative to DMSO control. Data in panels (E–I) was analyzed using two-way ANOVA with alpha of 0.05. Dunnett’s multiple comparison test was used. Statistical analyses are reported in Source Data. Error bars in figure represent mean ± SEM.

Source data are available online for this figure.

 © 2023 The Authors

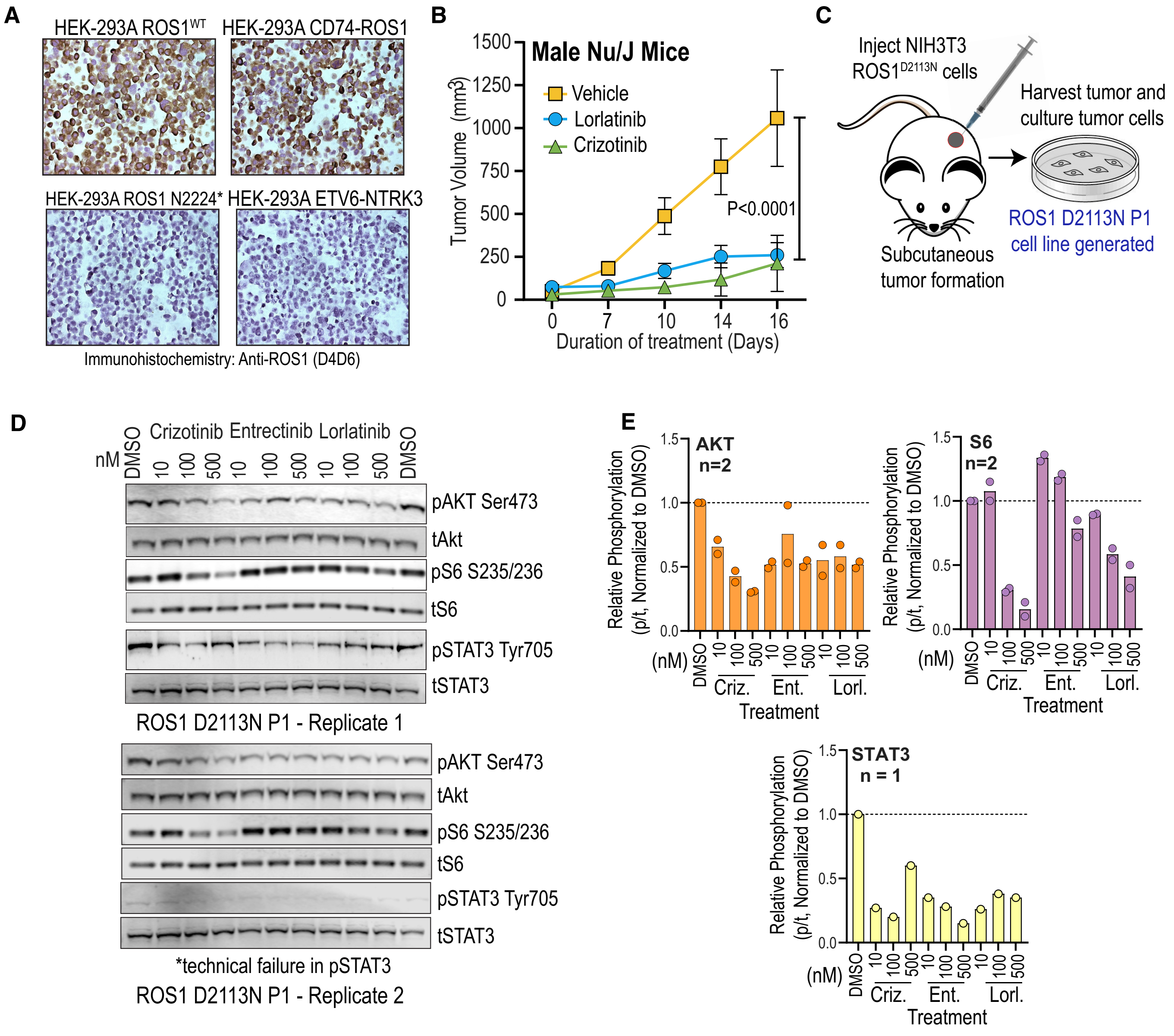

Figure EV5. ROS1^{D2113N} tumors and the tumor-derived cell line respond to ROS1-TKI treatment.

A Immunohistochemistry (ICC) of 20 μm sections from formalin-fixed, paraffin-embedded HEK-293A cell transduced with ROS1^{WT}, CD74-ROS1, ROS1^{N2224*}, and ETV6-NTRK3 to demonstrate specificity of ROS1 D4D6 antibody.

B Tumor growth of NIH-3T3 ROS1^{D2113N} cells subcutaneously injected into male Nu/J mice and treated for 14 days with vehicle, crizotinib (100 mg/kg), or lorlatinib (3 mg/kg). N = 5 apart from mice treated with crizotinib (N = 3), biological replicates. Two-way ANOVA with Dunnett's multiple comparisons test used to assess statistical significance (alpha 0.05). P value comparing vehicle vs. crizotinib (same value as vehicle vs. lorlatinib) on day 16 indicated within graph.

C Diagram showing generation of NIH-3T3 P1-ROS1^{D2113N} cell line from subcutaneous tumor in Nu/J mice.

D Immunoblots on NIH-3T3 P1-ROS1^{D2113N} cell lysates generated after treatment of cells with 10, 100 and 500 nM crizotinib, entrectinib and lorlatinib and immunoblotting with phospho-site specific antibodies as indicated in panel.

E Densitometry of replicate immunoblot experiments for evaluating effects of ROS1 TKI in NIH-3T3 P1-ROS1^{D2113N} tumor derived cells on AKT and S6 phosphorylation. N = 2 biological replicates except for phospho-STAT3, which is N = 1 because of technical failure in replicate experiment.

Source data are available online for this figure.

© 2023 The Authors
