# Supplementary figures and images for: Discovery of oncogenic ROS1 missense mutations with sensitivity to tyrosine kinase inhibitors
Source: EMBO Mol Med. 2023 Aug 17;15(10):e17367. doi: 10.15252/emmm.202217367 (PMC10565643; doi:10.15252/emmm.202217367)

pROS1 Y2274

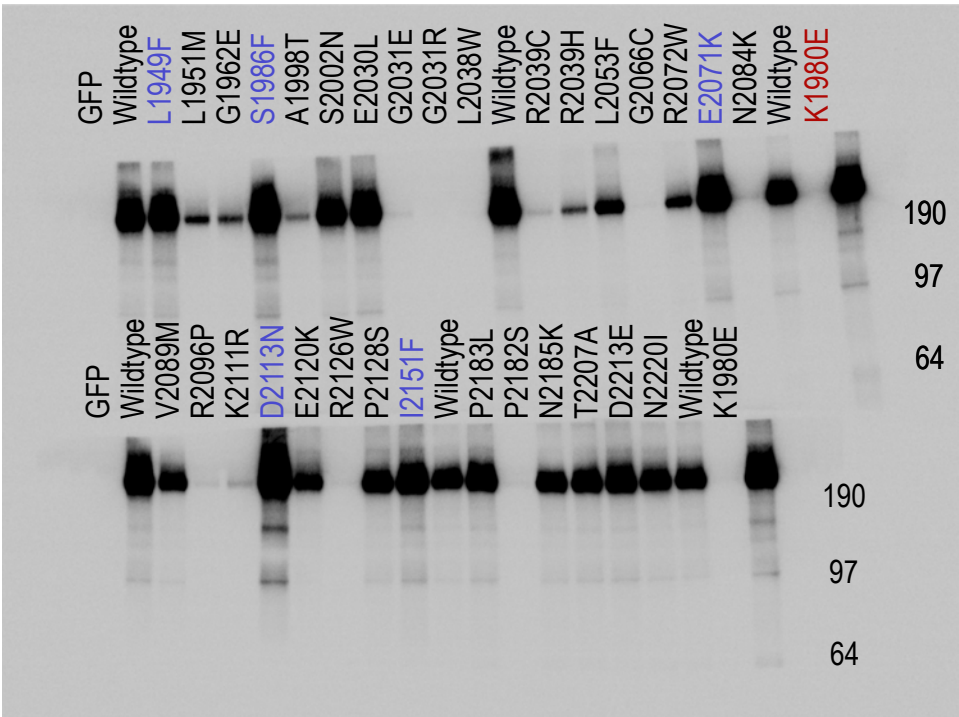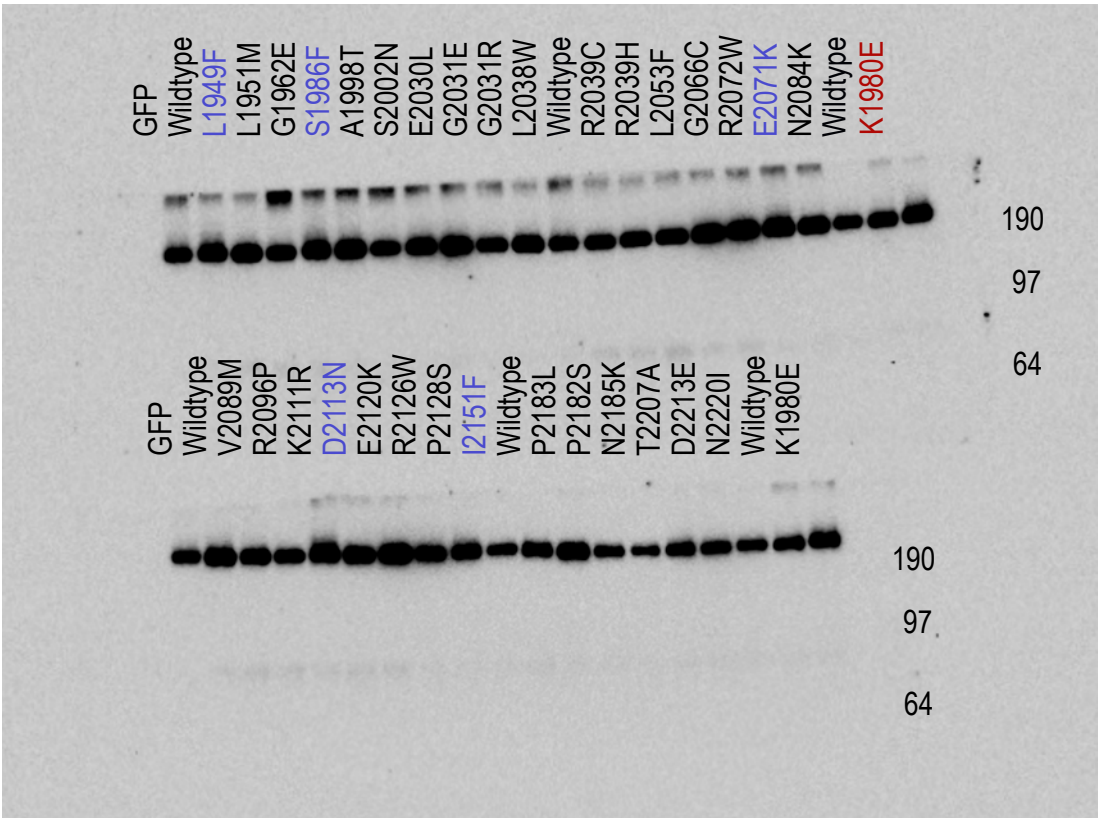

total ROS1

Supplement: Supplementary file 12 — Source Data for Figure 1 [file EMMM-15-e17367-s009.zip › Fig.1/1B.pdf]

# pROS1 Y2274

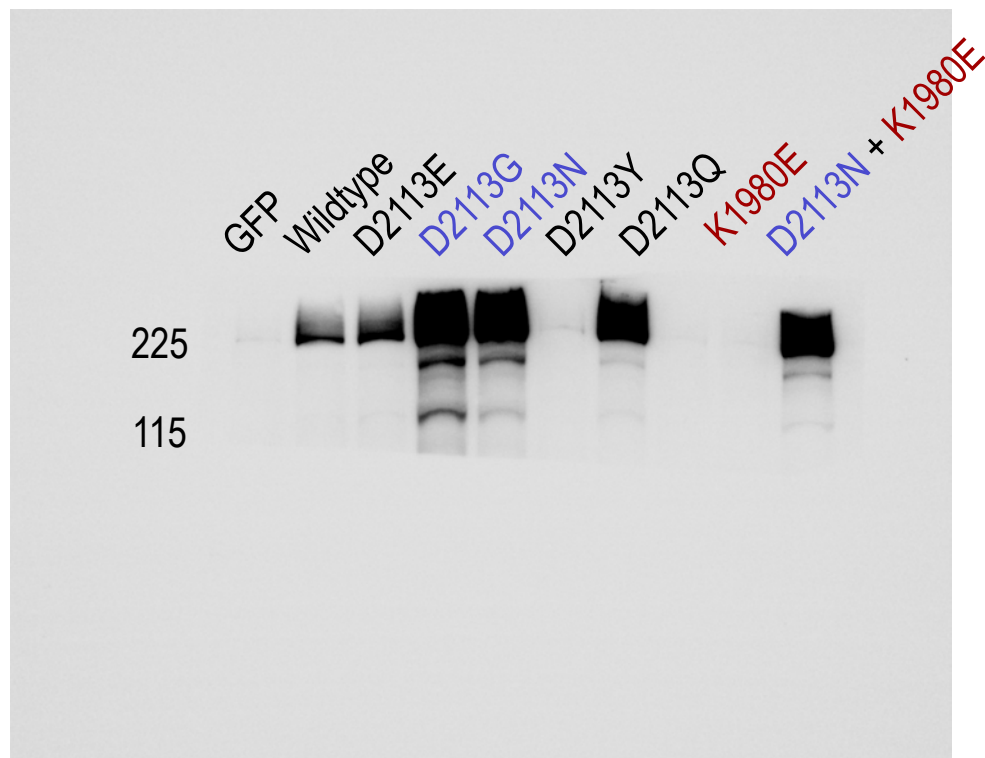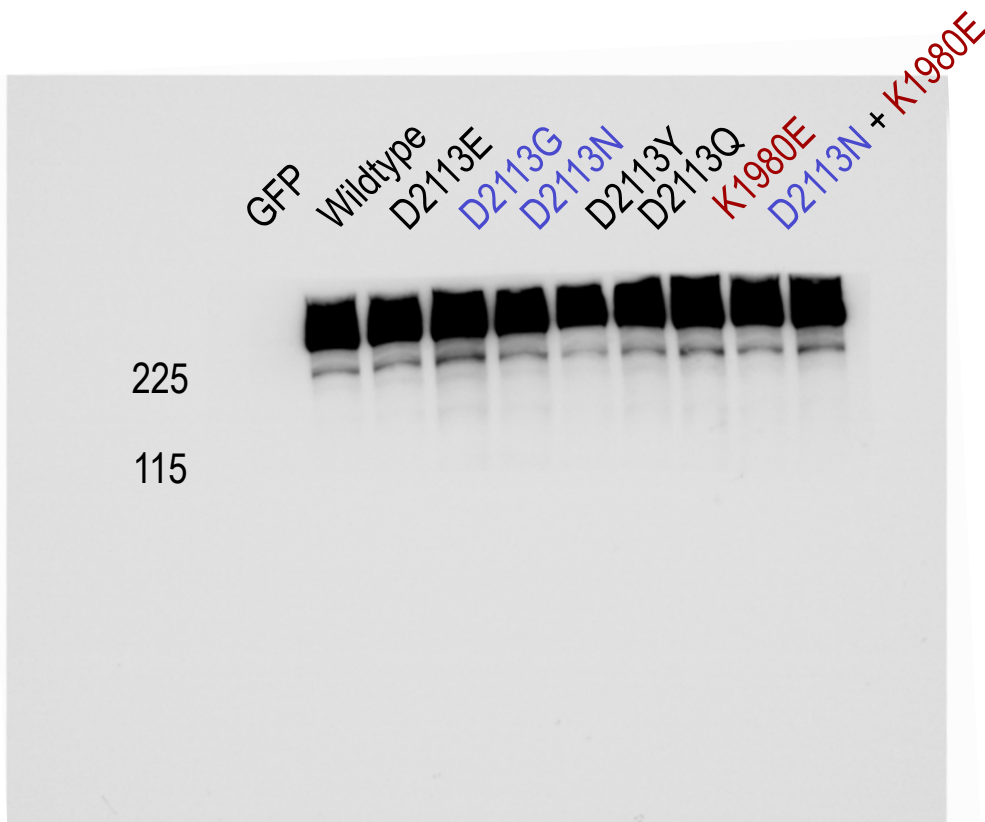

total ROS1

Supplement: Supplementary file 12 — Source Data for Figure 1 [file EMMM-15-e17367-s009.zip › Fig.1/1D.pdf]

# pROS1 Y2274

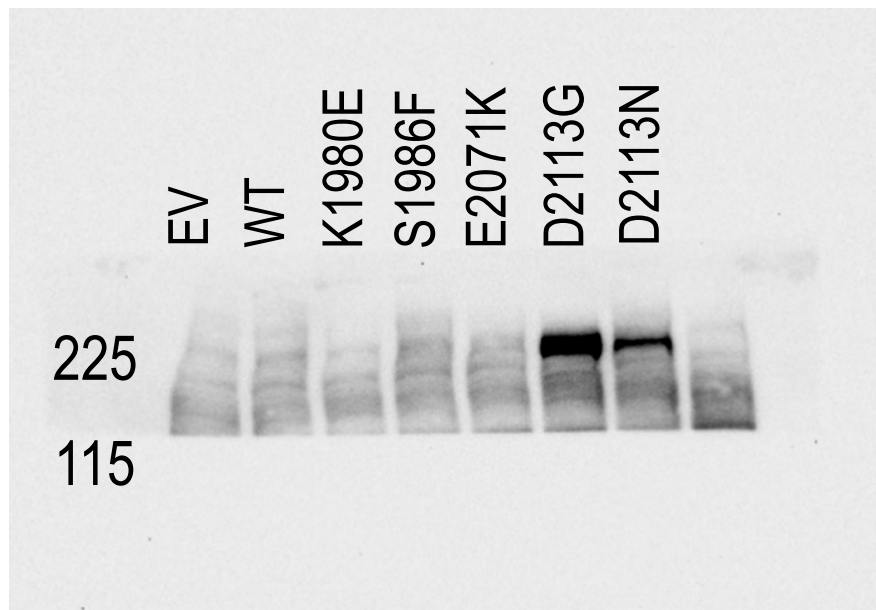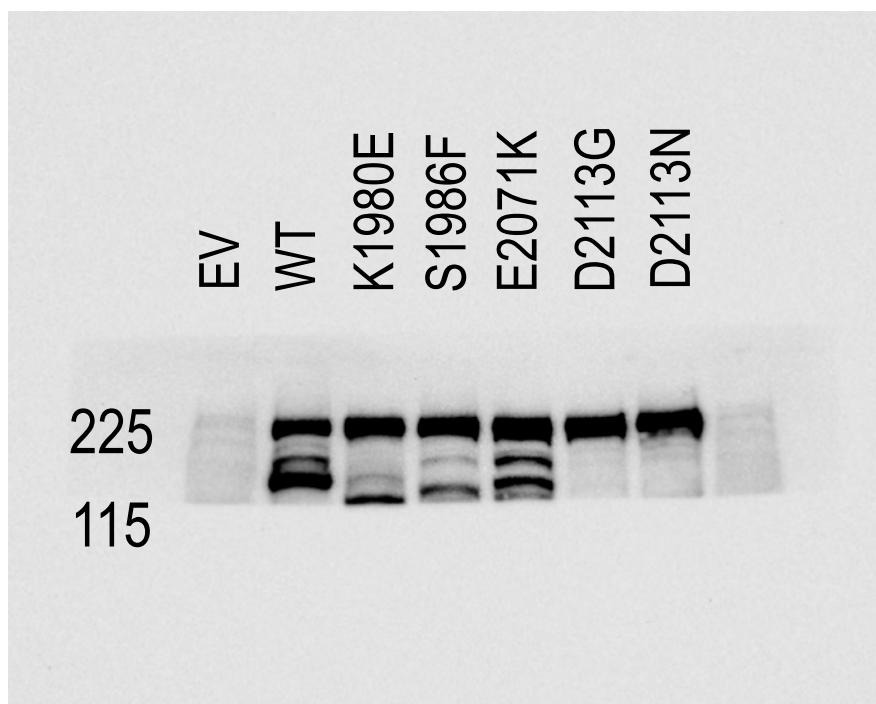

# total ROS1

Supplement: Supplementary file 13 — Source Data for Figure 2 [file EMMM-15-e17367-s005.zip › Fig.2/2D.pdf]

Empty Vector

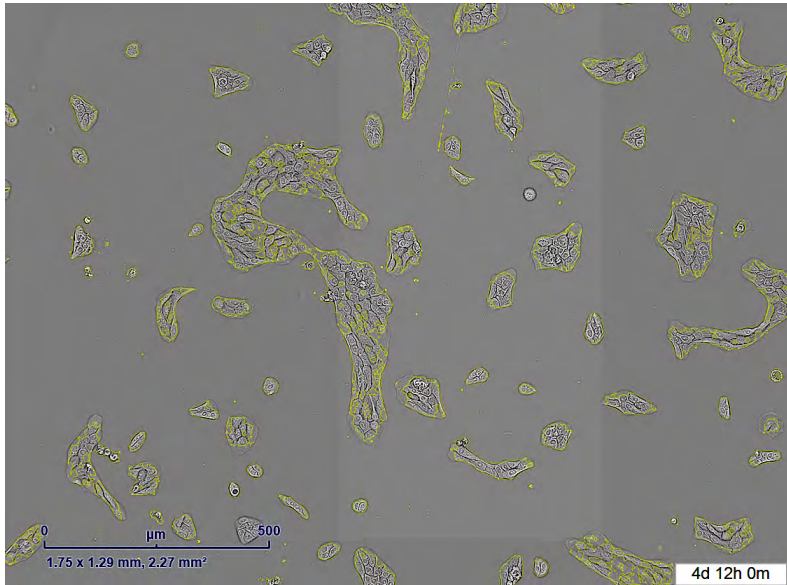

ROS1<sup>WT</sup>

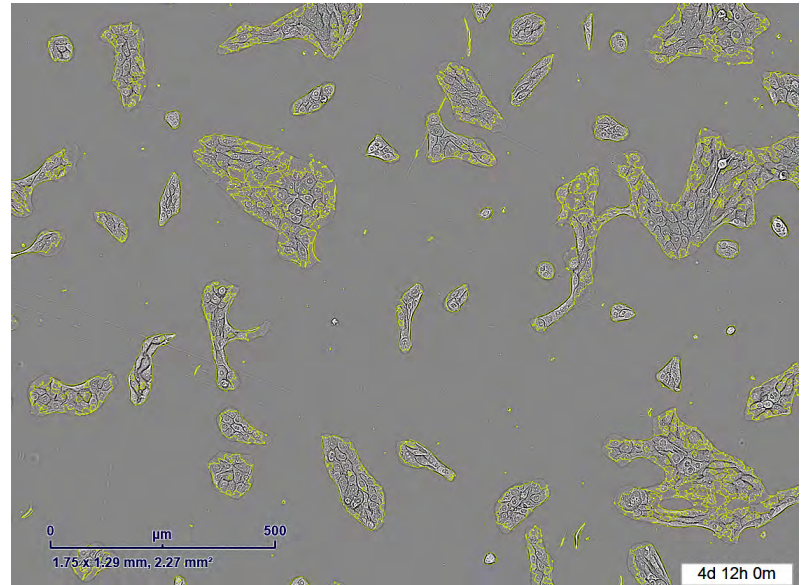

ROS1<sup>D2113G</sup>

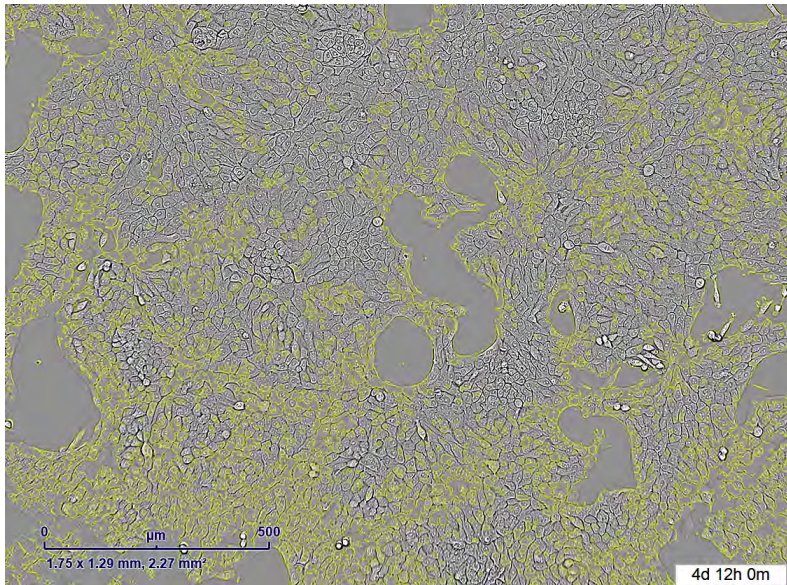

ROS1<sup>D2113N</sup>

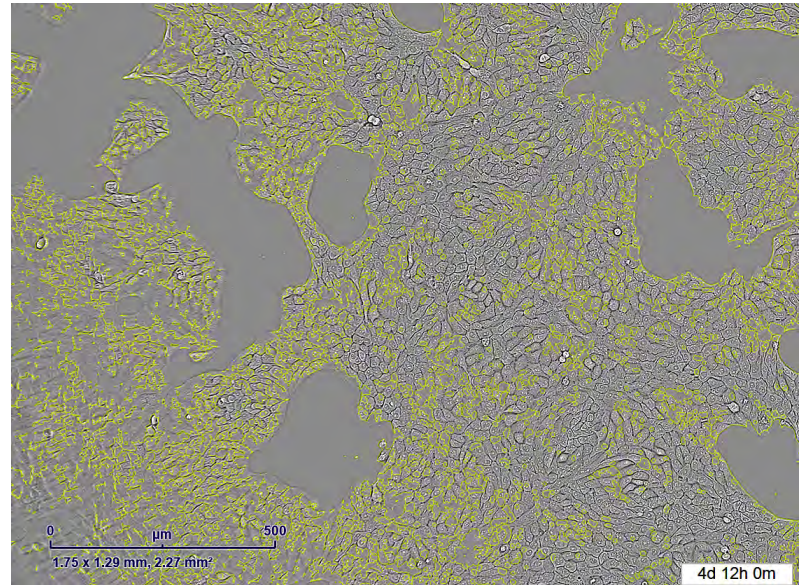

CD74-ROS1

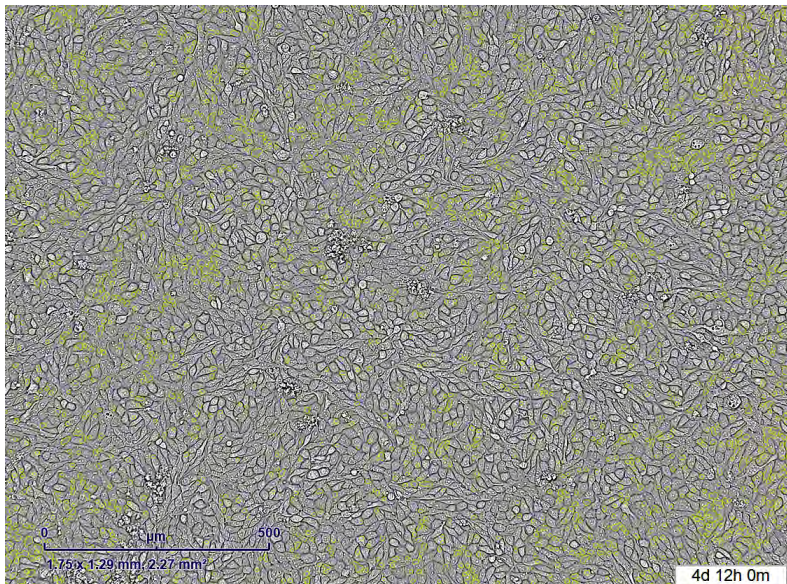

SLC-ROS1

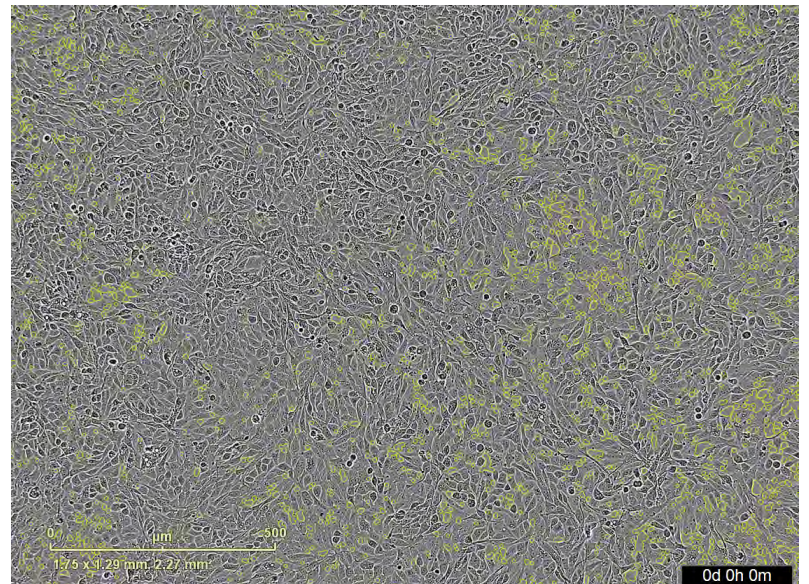

Supplement: Supplementary file 13 — Source Data for Figure 2 [file EMMM-15-e17367-s005.zip › Fig.2/2F.pdf]

## pROS1 Y2274

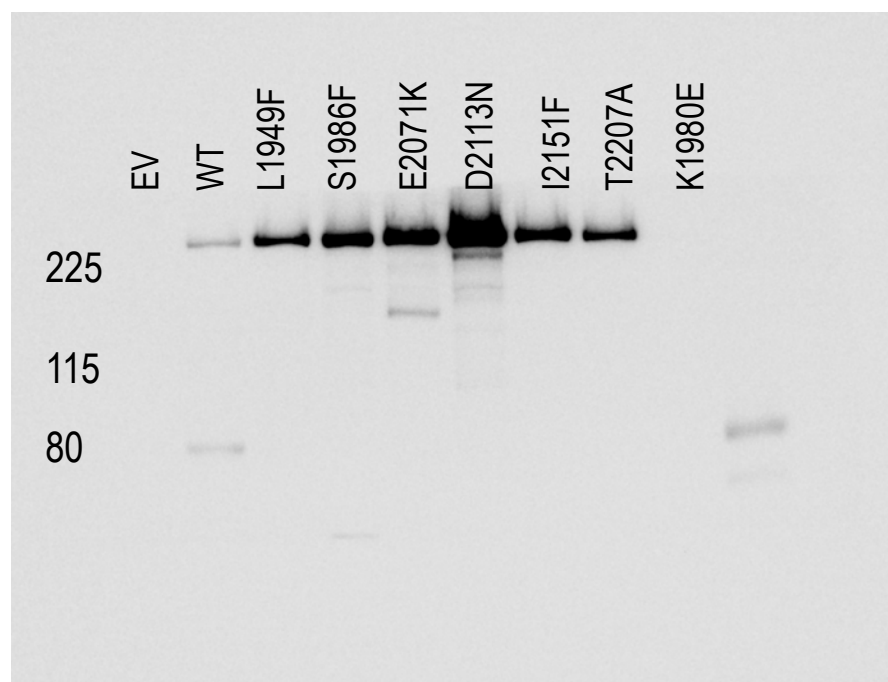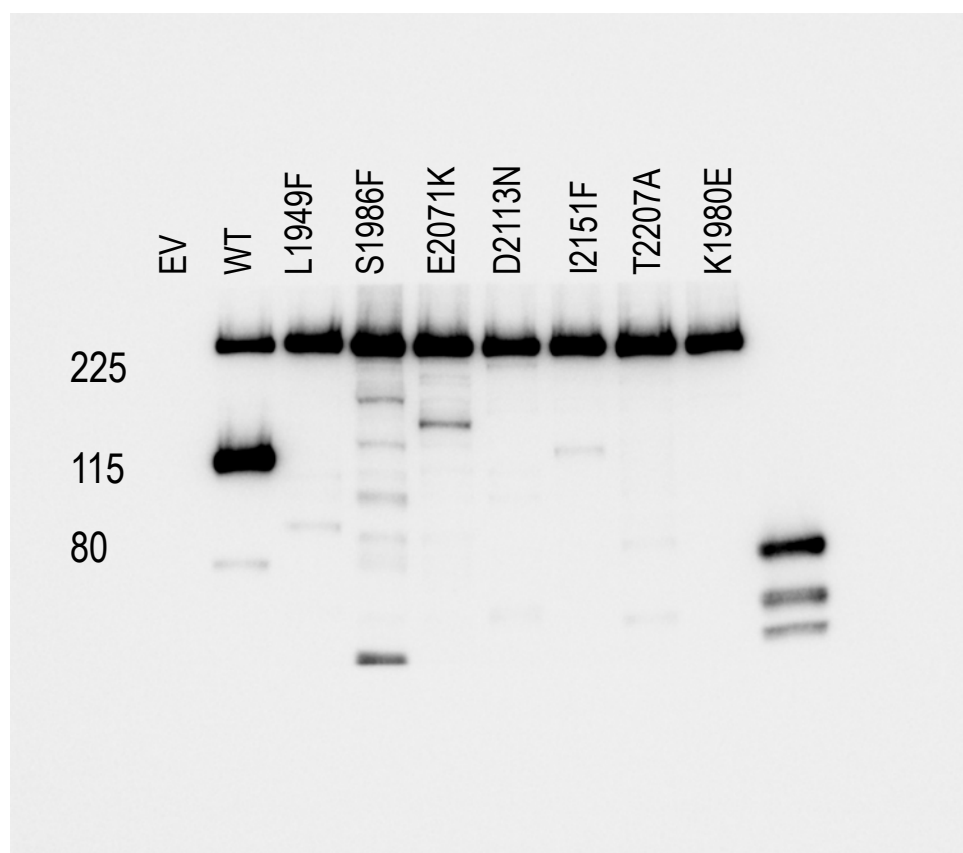

total ROS1

Supplement: Supplementary file 13 — Source Data for Figure 2 [file EMMM-15-e17367-s005.zip › Fig.2/2A.pdf]

pSTAT3

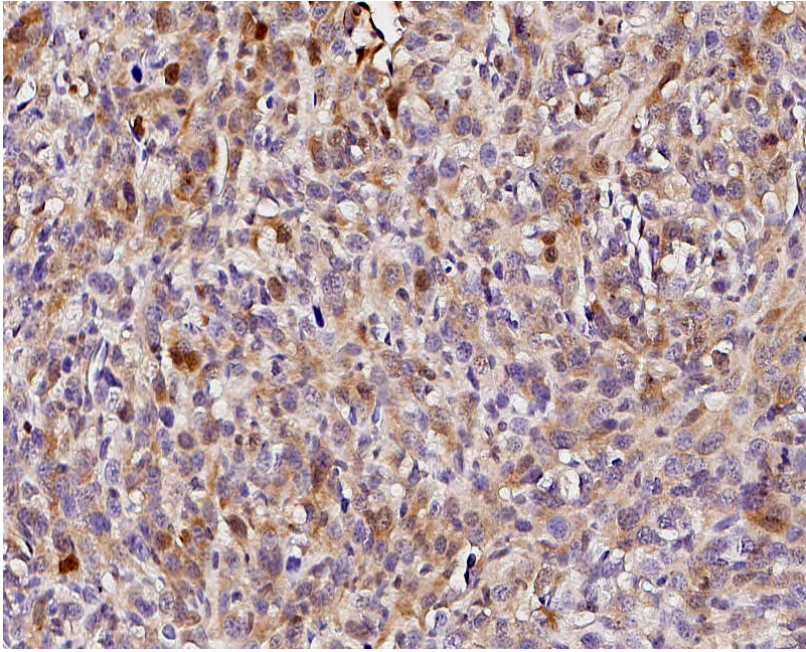

pSHP2

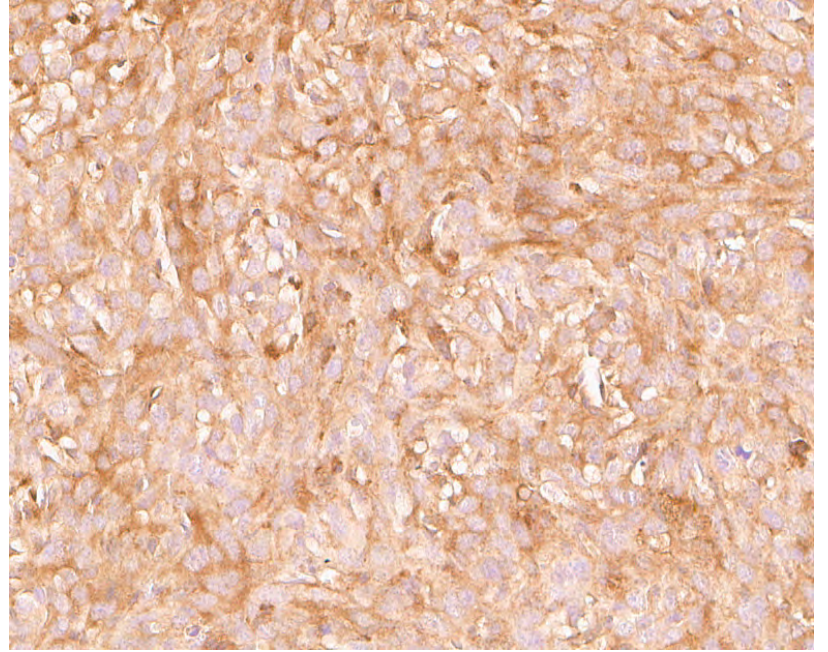

tROS1

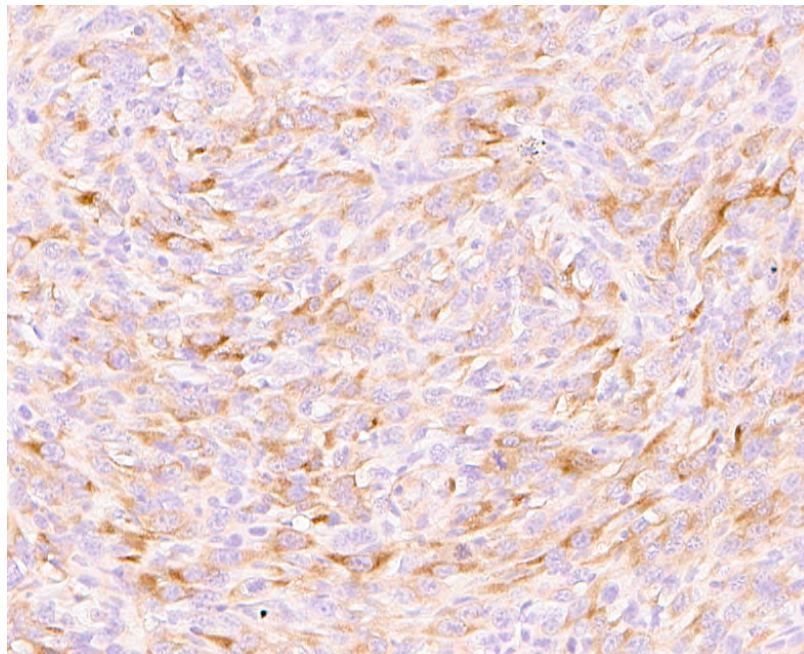

Supplement: Supplementary file 16 — Source Data for Figure 7 [file EMMM-15-e17367-s008.zip › Fig.7/7C.pdf]
