# Supplementary material for: Discovery of oncogenic ROS1 missense mutations with sensitivity to tyrosine kinase inhibitors
Source: EMBO Mol Med. 2023 Aug 17;15(10):e17367. doi: 10.15252/emmm.202217367 (PMC10565643; doi:10.15252/emmm.202217367)

HEK-293

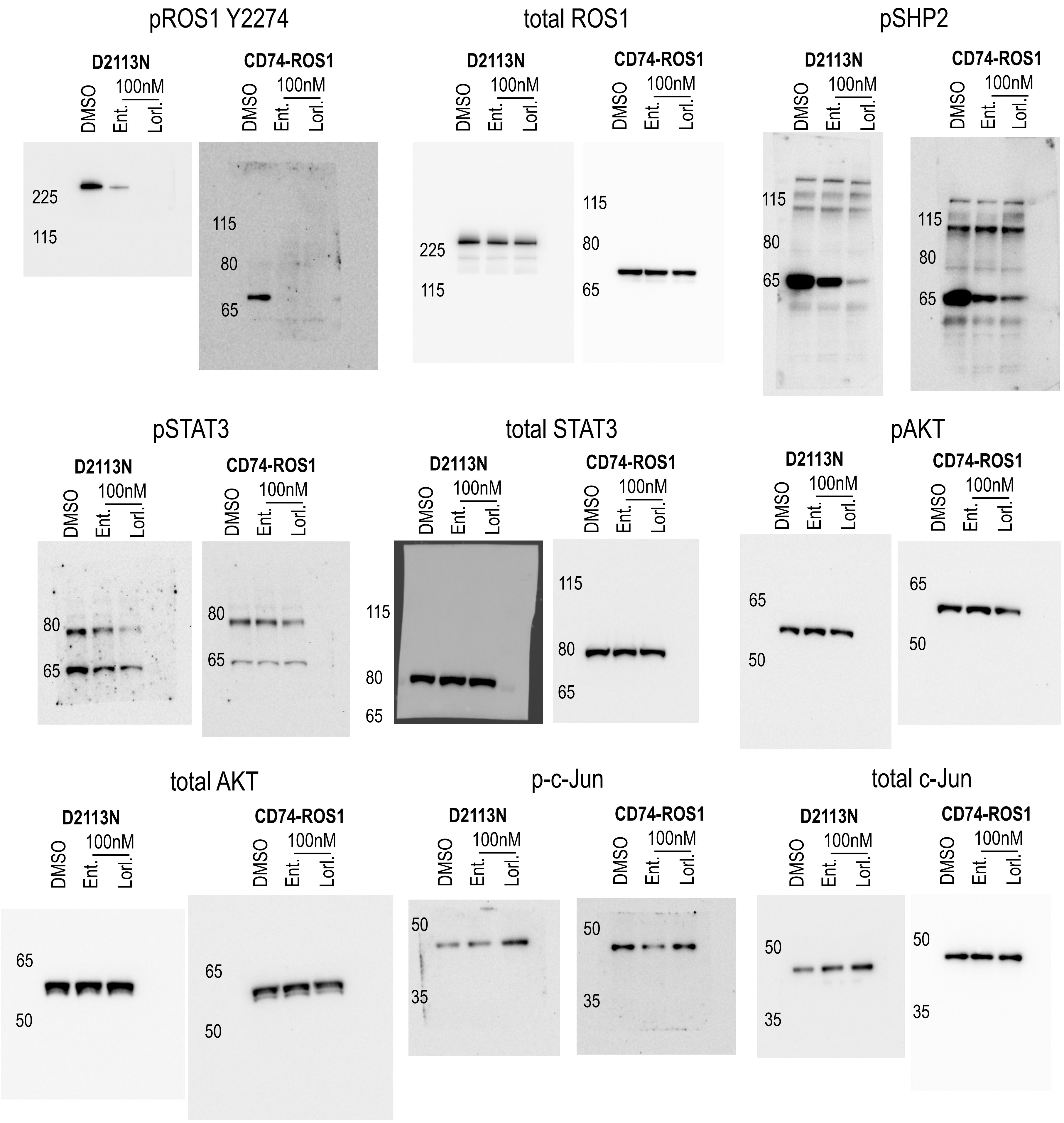

pROS1 Y2274
total ROS1
pSHP2
pSTAT3
total STAT3
pAKT
total AKT
p-c-Jun
total c-Jun
D2113N
CD74-ROS1
DMSO
100nM
Ent.
Lorl.
225
115
80
65
50
35

HEK-293 Blots (Contd.)

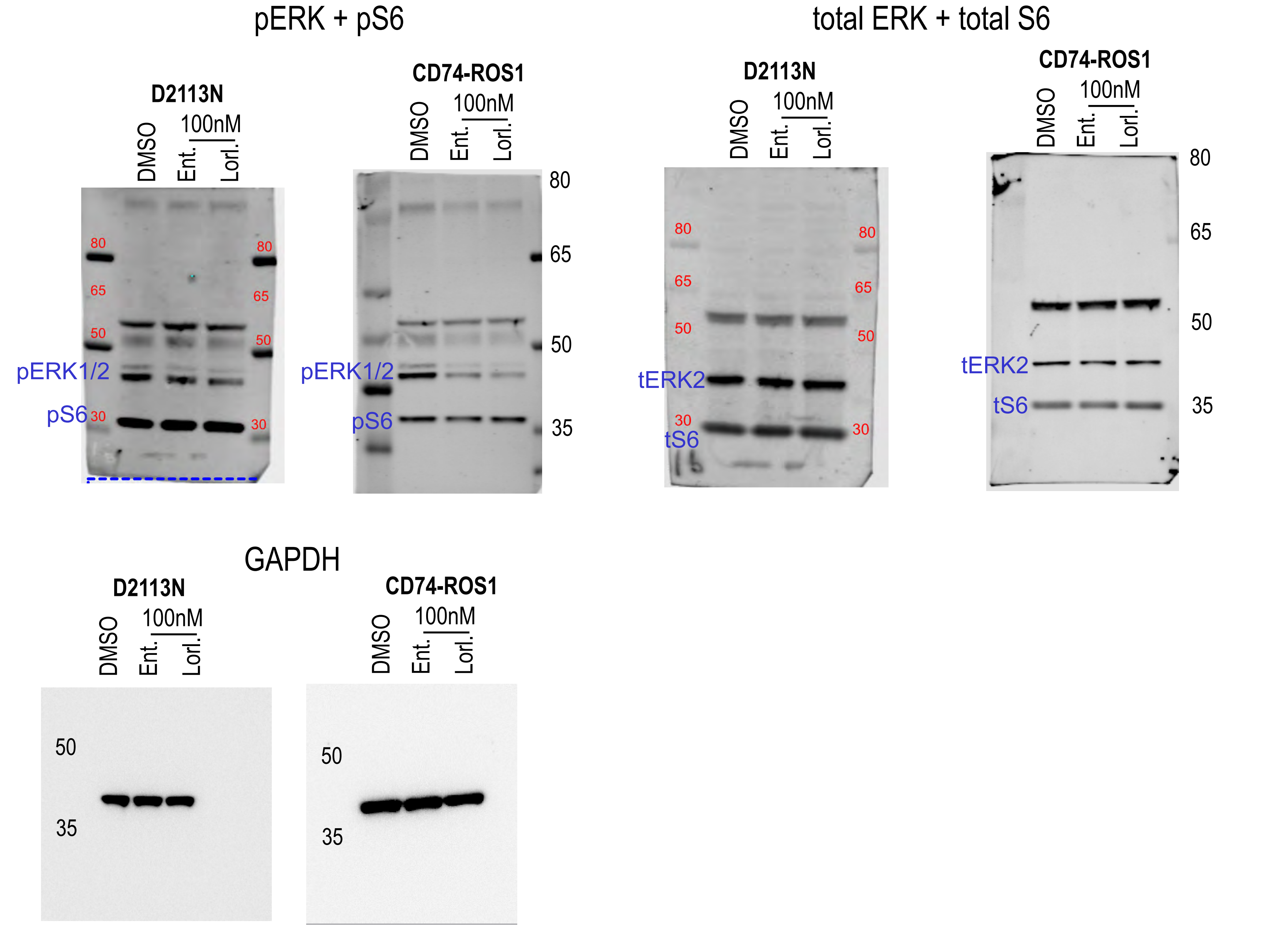

NIH-3T3 Blots

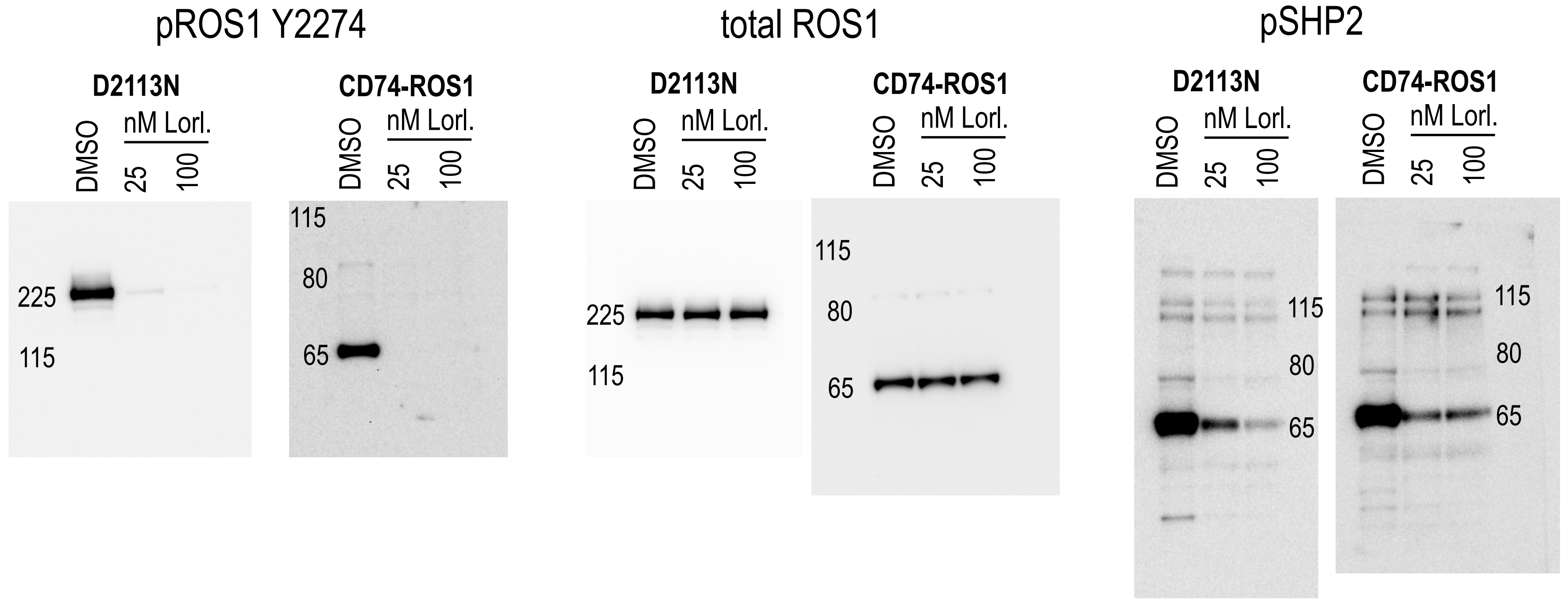

NIH-3T3 blots (contd.)

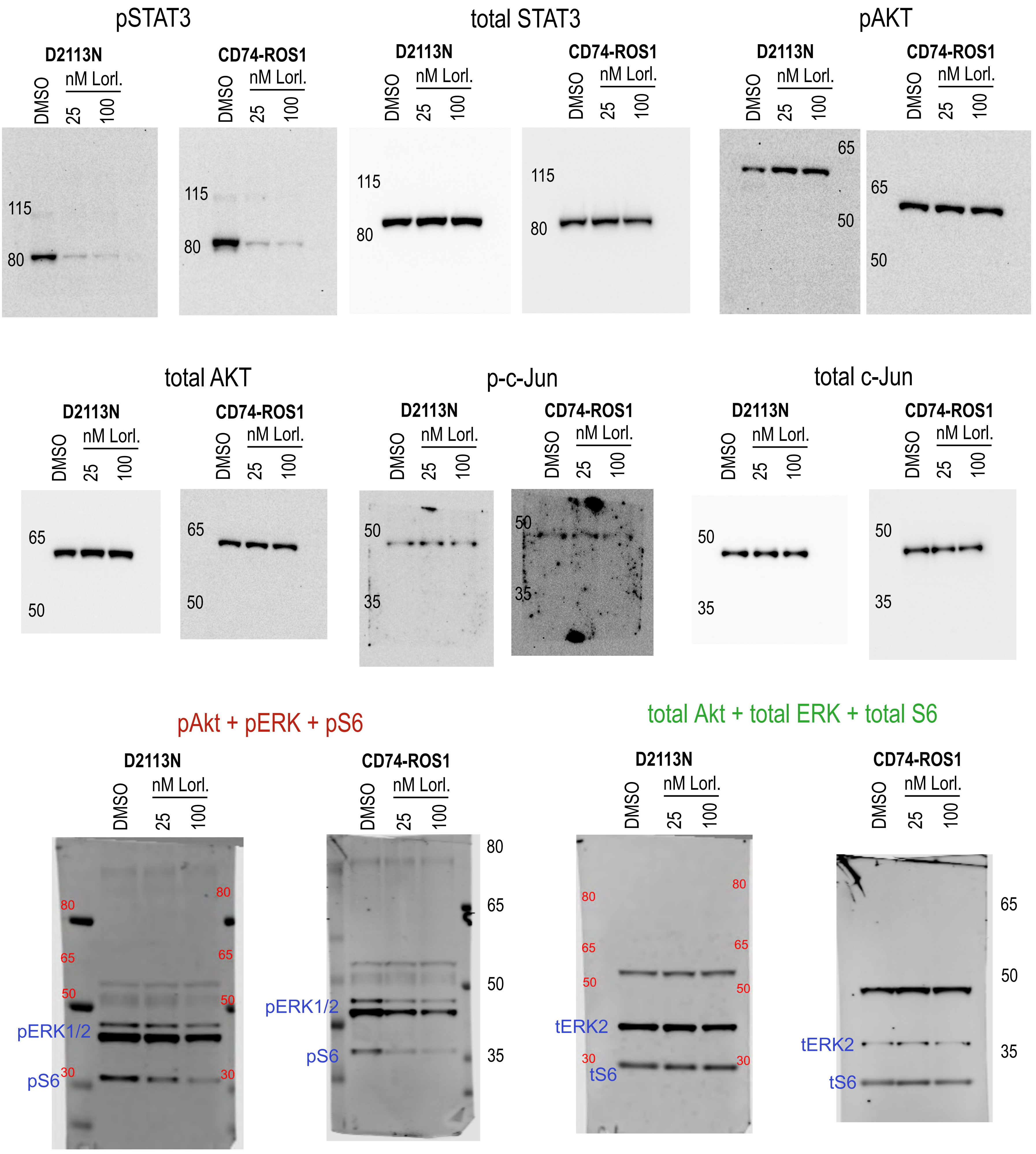

NIH-3T3 blots (contd.)

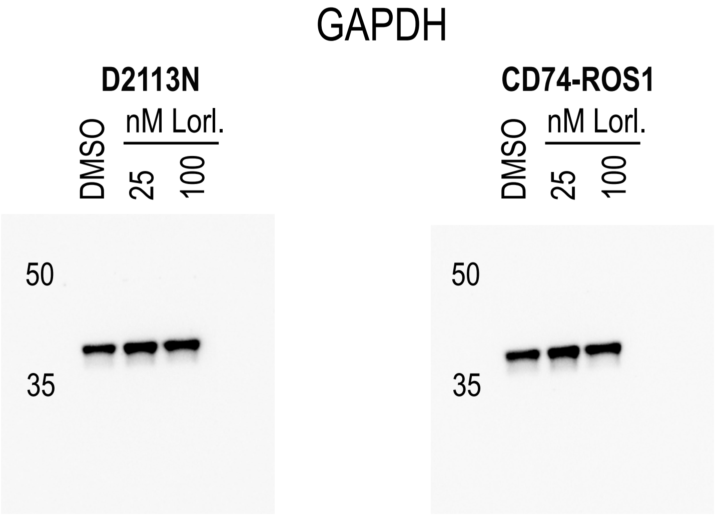

Supplement: Supplementary file 15 — Source Data for Figure 5 [file EMMM-15-e17367-s014.zip › Fig.5/5C.pdf]
