# Supplementary material for: Discovery of oncogenic ROS1 missense mutations with sensitivity to tyrosine kinase inhibitors
Source: EMBO Mol Med. 2023 Aug 17;15(10):e17367. doi: 10.15252/emmm.202217367 (PMC10565643; doi:10.15252/emmm.202217367)

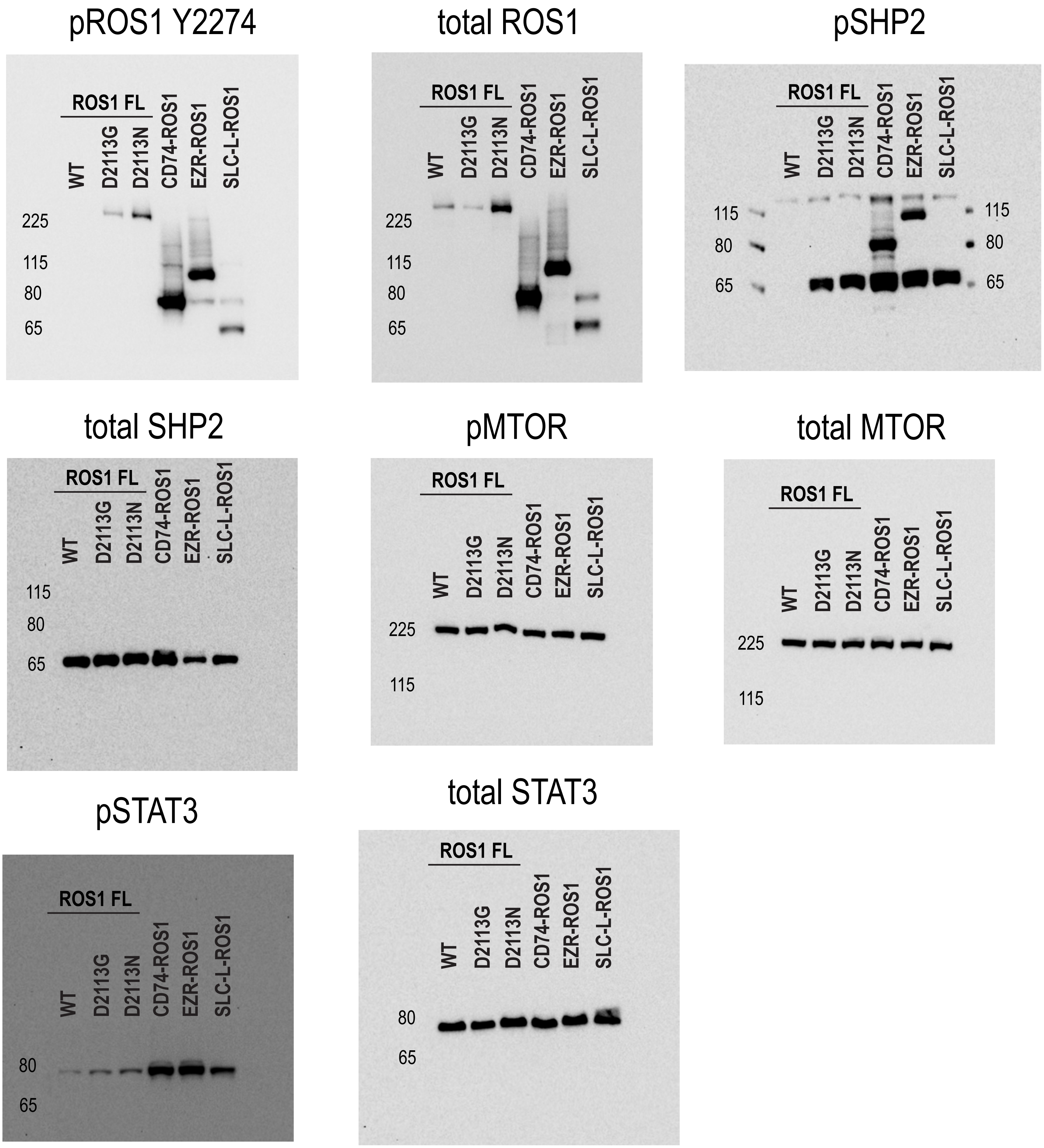
pROS1 Y2274
ROS1 FL
WT
D2113G
D2113N
CD74-ROS1
EZR-ROS1
SLC-L-ROS1
225
115
80
65
total ROS1
ROS1 FL
WT
D2113G
D2113N
CD74-ROS1
EZR-ROS1
SLC-L-ROS1
225
115
80
65
pSHP2
ROS1 FL
WT
D2113G
D2113N
CD74-ROS1
EZR-ROS1
SLC-L-ROS1
115
80
65
115
80
65
total SHP2
ROS1 FL
WT
D2113G
D2113N
CD74-ROS1
EZR-ROS1
SLC-L-ROS1
115
80
65
pMTOR
ROS1 FL
WT
D2113G
D2113N
CD74-ROS1
EZR-ROS1
SLC-L-ROS1
225
115
total MTOR
ROS1 FL
WT
D2113G
D2113N
CD74-ROS1
EZR-ROS1
SLC-L-ROS1
225
115
pSTAT3
ROS1 FL
WT
D2113G
D2113N
CD74-ROS1
EZR-ROS1
SLC-L-ROS1
80
65
total STAT3
ROS1 FL
WT
D2113G
D2113N
CD74-ROS1
EZR-ROS1
SLC-L-ROS1
80
65

p-c-Jun

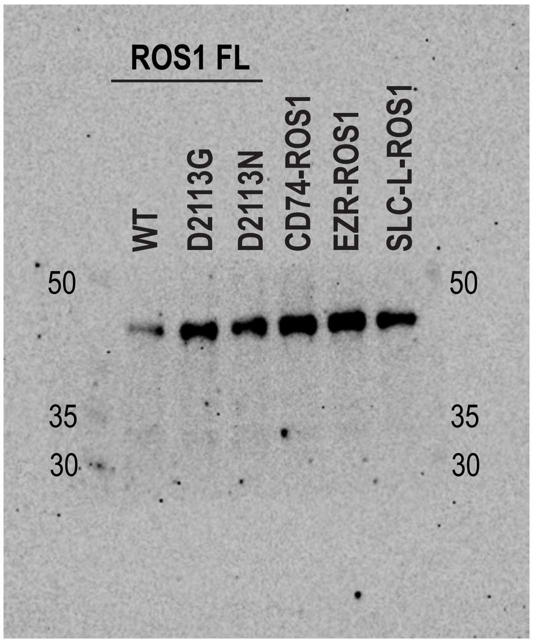

total c-Jun

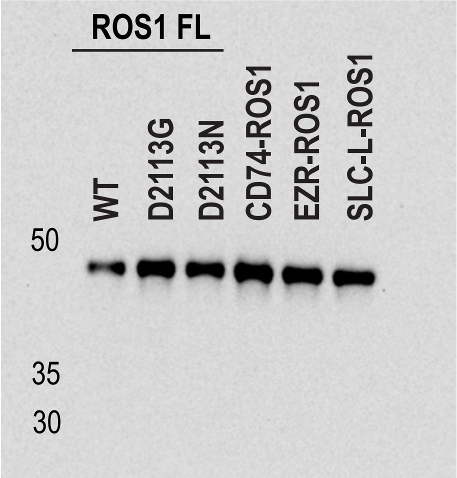

pERK + pS6

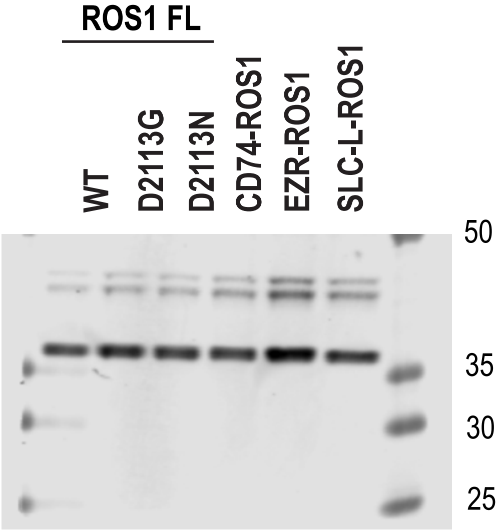

total ERK + total S6

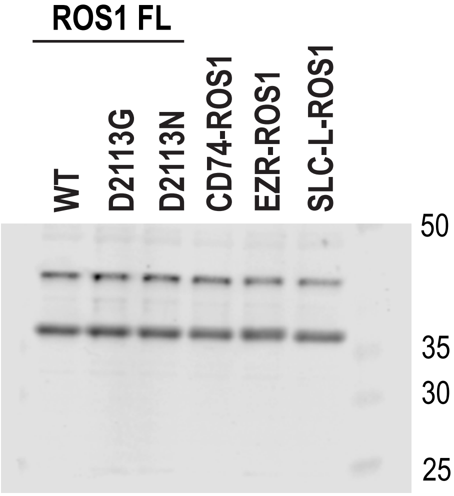

Supplement: Supplementary file 15 — Source Data for Figure 5 [file EMMM-15-e17367-s014.zip › Fig.5/5A.pdf]
