# Supplementary material for: Discovery of oncogenic ROS1 missense mutations with sensitivity to tyrosine kinase inhibitors
Source: EMBO Mol Med. 2023 Aug 17;15(10):e17367. doi: 10.15252/emmm.202217367 (PMC10565643; doi:10.15252/emmm.202217367)

Vehicle #1 0 days

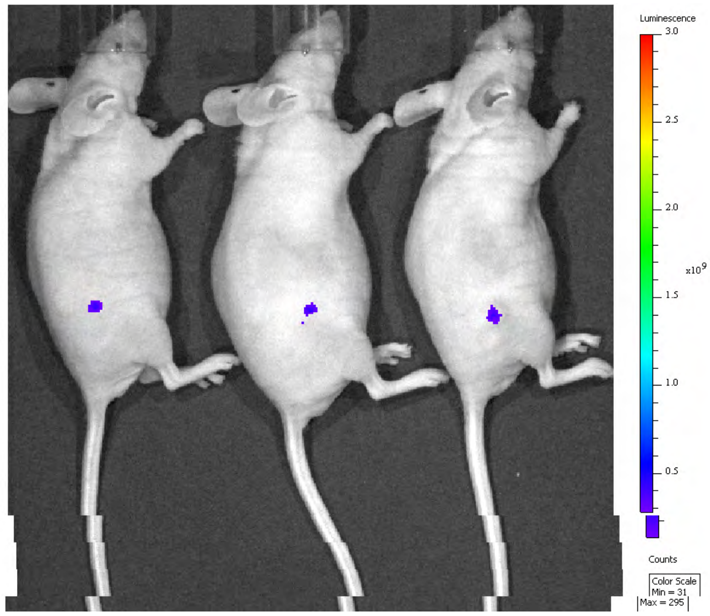

Vehicle #1 14 days

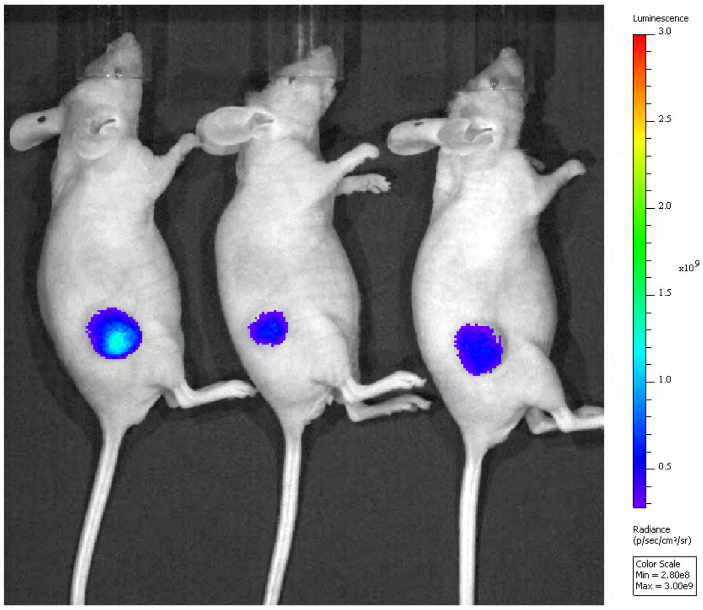

Vehicle #2 14 days

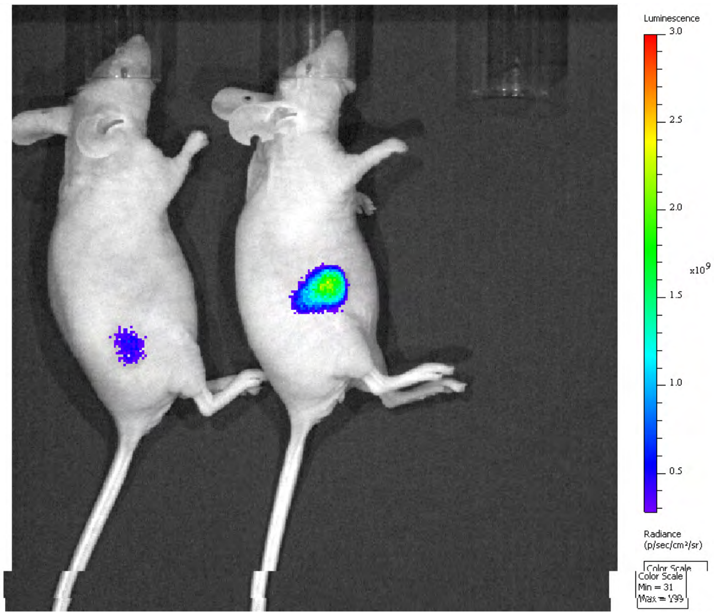

Lorlatinib #1 0 days

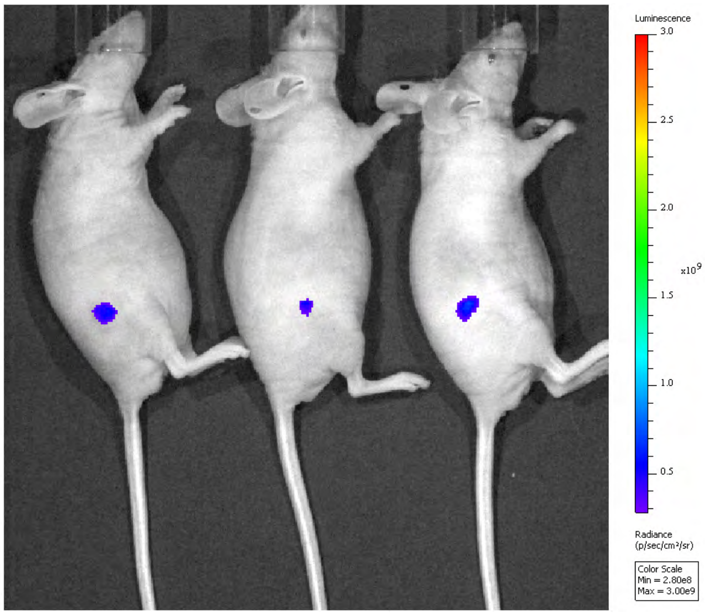

Lorlatinib #2 0 days

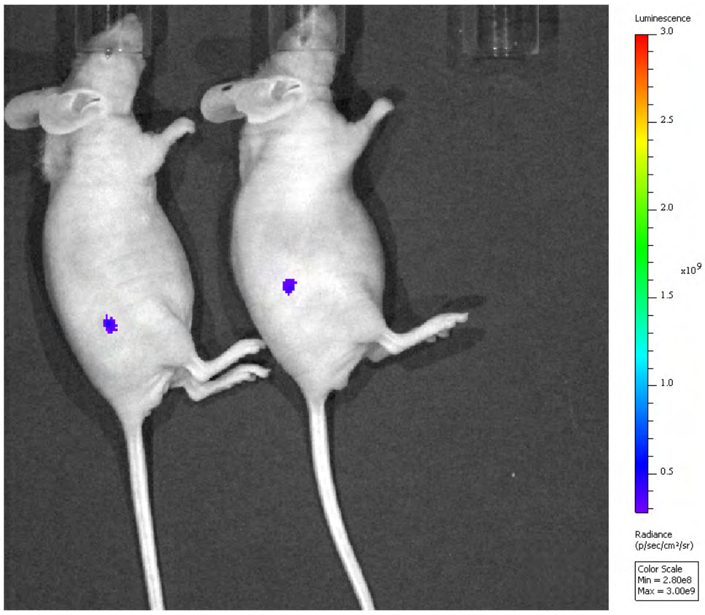

Lorlatinib #1 14 days

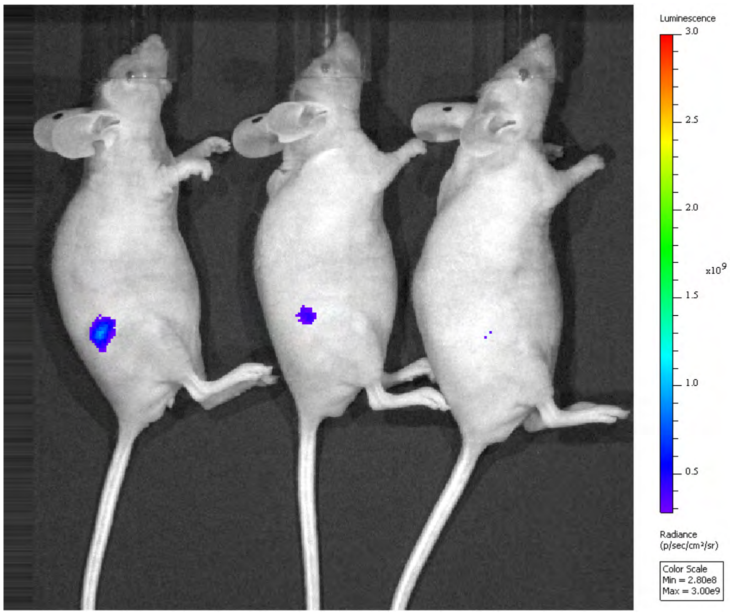

Lorlatinib #2 14 days

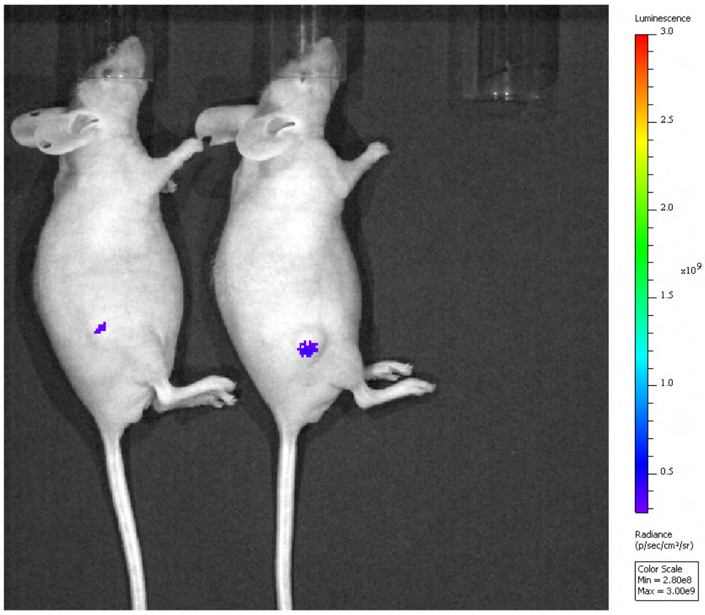

Supplement: Supplementary file 16 — Source Data for Figure 7 [file EMMM-15-e17367-s008.zip › Fig.7/7D.pdf]
