# Supplementary material for: Discovery of oncogenic ROS1 missense mutations with sensitivity to tyrosine kinase inhibitors
Source: EMBO Mol Med. 2023 Aug 17;15(10):e17367. doi: 10.15252/emmm.202217367 (PMC10565643; doi:10.15252/emmm.202217367)
Supplement: Supplementary file 16 — Source Data for Figure 7 [file EMMM-15-e17367-s008.zip › Fig.7/7G.pdf]

Set 1

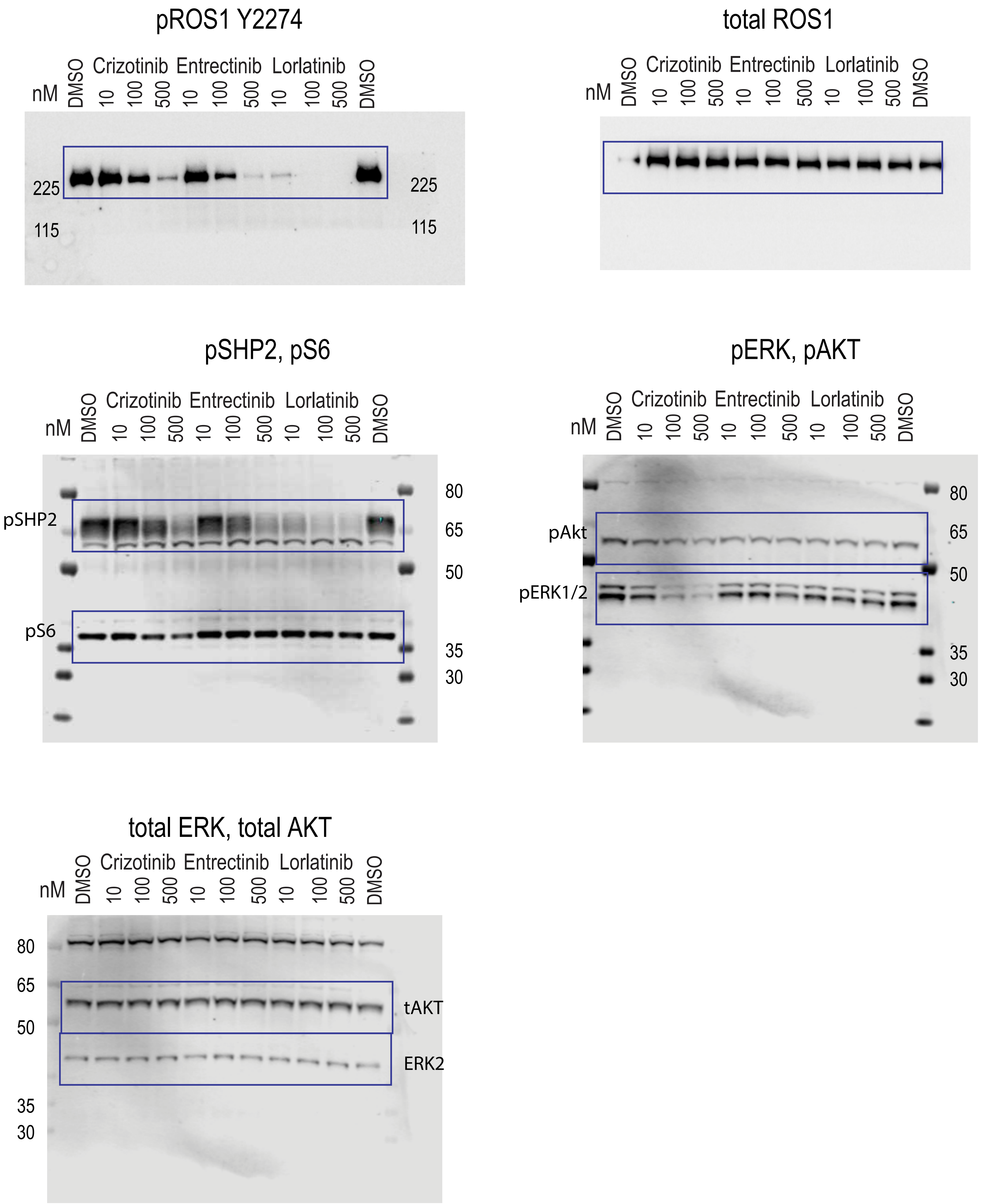
pROS1 Y2274
nM DMSO Crizotinib 10 100 500 Entrectinib 10 100 500 Lorlatinib 10 100 500 DMSO
225
115
225
115
total ROS1
nM DMSO Crizotinib 10 100 500 Entrectinib 10 100 500 Lorlatinib 10 100 500 DMSO
pSHP2, pS6
nM DMSO Crizotinib 10 100 500 Entrectinib 10 100 500 Lorlatinib 10 100 500 DMSO
pSHP2
pS6
80
65
50
35
30
pERK, pAKT
nM DMSO Crizotinib 10 100 500 Entrectinib 10 100 500 Lorlatinib 10 100 500 DMSO
pAkt
pERK1/2
80
65
50
35
30
total ERK, total AKT
nM DMSO Crizotinib 10 100 500 Entrectinib 10 100 500 Lorlatinib 10 100 500 DMSO
80
65
50
35
30
tAKT
ERK2

Set 2

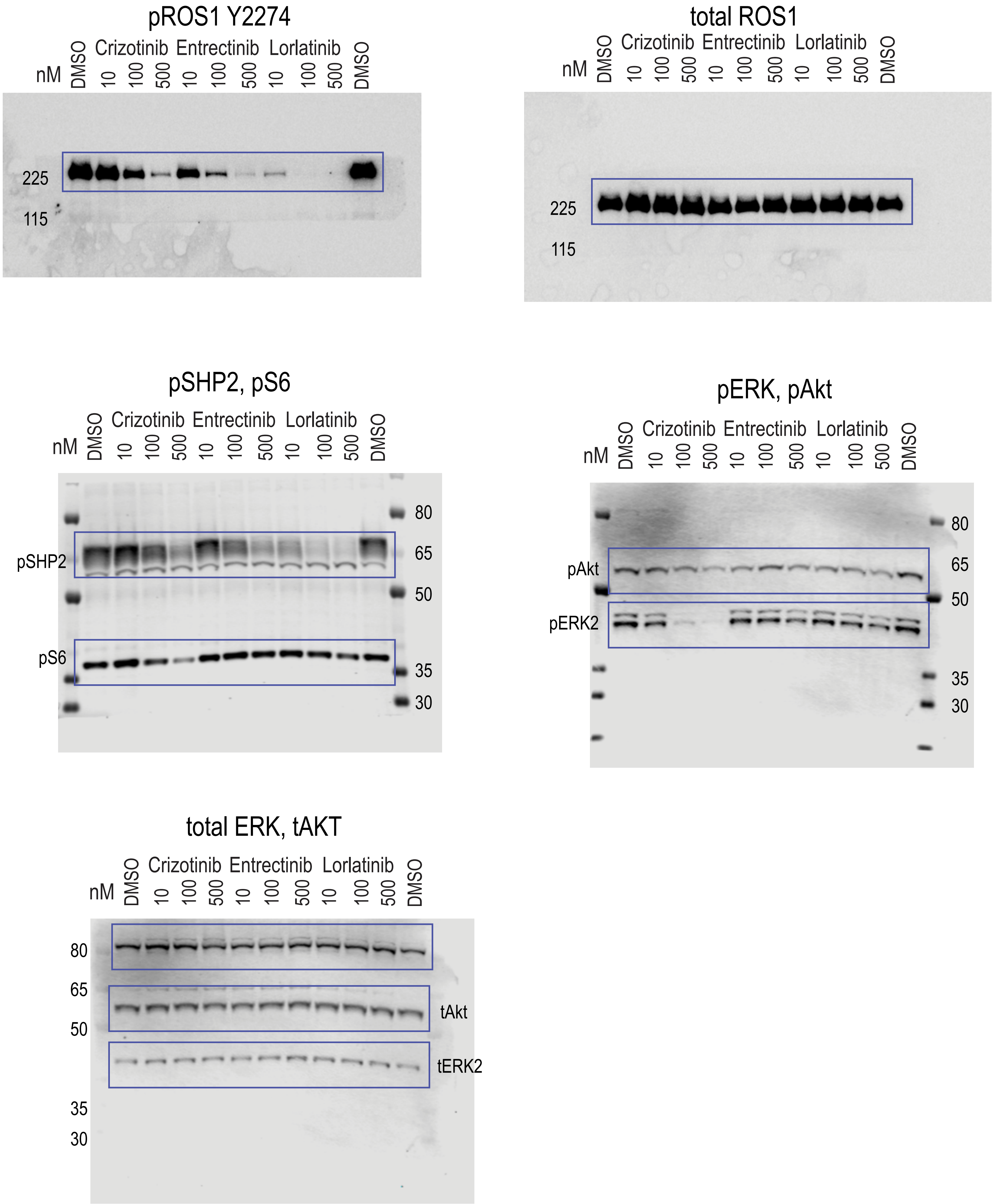
pROS1 Y2274
nM DMSO Crizotinib 10 100 500 Entrectinib 10 100 500 Lorlatinib 10 100 500 DMSO
225
115
total ROS1
nM DMSO Crizotinib 10 100 500 Entrectinib 10 100 500 Lorlatinib 10 100 500 DMSO
225
115
pSHP2, pS6
nM DMSO Crizotinib 10 100 500 Entrectinib 10 100 500 Lorlatinib 10 100 500 DMSO
pSHP2
pS6
80
65
50
35
30
pERK, pAkt
nM DMSO Crizotinib 10 100 500 Entrectinib 10 100 500 Lorlatinib 10 100 500 DMSO
pAkt
pERK2
80
65
50
35
30
total ERK, tAKT
nM DMSO Crizotinib 10 100 500 Entrectinib 10 100 500 Lorlatinib 10 100 500 DMSO
80
65
50
35
30
tAkt
tERK2
