# Supplementary material for: Discovery of oncogenic ROS1 missense mutations with sensitivity to tyrosine kinase inhibitors
Source: EMBO Mol Med. 2023 Aug 17;15(10):e17367. doi: 10.15252/emmm.202217367 (PMC10565643; doi:10.15252/emmm.202217367)
Supplement: Supplementary file 17 [file EMMM-15-e17367-s006.pdf]

EMBO Molecular Medicine

Discovery of oncogenic ROS1 missense mutations with sensitivity to tyrosine kinase inhibitors

Sudarshan Iyer, Kevin Nusser, Kristen Jones, Clare keddy, Pushkar Shinde, Catherine Beach, Erin Aguero, Jeremy Force, Ujwal Shinde, and Monika Davare
DOI: 10.15252/emmm.202217367

Corresponding author: Monika Davare (davarem@ohsu.edu)

Review Timeline:

Submission Date:	27th Dec 22
Editorial Decision:	1st Feb 23
Revision Received:	16th Jun 23
Editorial Decision:	19th Jul 23
Revision Received:	24th Jul 23
Accepted:	26th Jul 23

Editor: Lise Roth

Transaction Report:

(Note: With the exception of the correction of typographical or spelling errors that could be a source of ambiguity, letters and reports are not edited. Depending on transfer agreements, referee reports obtained elsewhere may or may not be included in this compilation. Referee reports are anonymous unless the Referee chooses to sign their reports.)

1st Feb 2023

Dear Dr. Davare,

Thank you for the submission of your manuscript to EMBO Molecular Medicine, and please accept my apologies for the delay in getting back to you as it took some time to recruit referees after the holiday break. We have now received feedback from the three reviewers who agreed to evaluate your manuscript. As you will see from the reports below, the referees acknowledge the interest of the study and are overall supporting publication of your work pending appropriate revisions.

Addressing the reviewers' concerns in full will be necessary for further considering the manuscript in our journal, and acceptance of the manuscript will entail a second round of review. In particular, the low frequency of the mutation should be clearly stated and discussed, and the signaling downstream from ROS1 (MAPK, PI3K) should be further investigated. EMBO Molecular Medicine encourages a single round of revision only and therefore, acceptance or rejection of the manuscript will depend on the completeness of your responses included in the next, final version of the manuscript. For this reason, and to save you from any frustrations in the end, I would strongly advise against returning an incomplete revision.

If you would like to discuss further the points raised by the referees, I am available to do so via email or video. Let me know if you are interested in this option.

Revised manuscripts should be submitted within three months of a request for revision; they will otherwise be treated as new submissions, except under exceptional circumstances in which a short extension is obtained from the editor.

When submitting your revised manuscript, please carefully review the instructions that follow below. We perform an initial quality control of all revised manuscripts before re-review; failure to include requested items will delay the evaluation of your revision.

We require:

1) A .docx formatted version of the manuscript text (including legends for main figures, EV figures and tables). Please make sure that the changes are highlighted to be clearly visible.

2) Individual production quality figure files as .eps, .tif, .jpg (one file per figure). For guidance, download the 'Figure Guide PDF' (https://www.embopress.org/page/journal/17574684/authorguide#figureformat).

3) At EMBO Press we ask authors to provide source data for the main figures. Our source data coordinator will contact you to discuss which figure panels we would need source data for and will also provide you with helpful tips on how to upload and organize the files.

4) A .docx formatted letter INCLUDING the reviewers' reports and your detailed point-by-point responses to their comments. As part of the EMBO Press transparent editorial process, the point-by-point response is part of the Review Process File (RPF), which will be published alongside your paper.

5) A complete author checklist, which you can download from our author guidelines (https://www.embopress.org/page/journal/17574684/authorguide#submissionofrevisions). Please insert information in the checklist that is also reflected in the manuscript. The completed author checklist will also be part of the RPF.

6) It is mandatory to include a 'Data Availability' section after the Materials and Methods. Before submitting your revision, primary datasets produced in this study need to be deposited in an appropriate public database, and the accession numbers and database listed under 'Data Availability'. Please remember to provide a reviewer password if the datasets are not yet public (see https://www.embopress.org/page/journal/17574684/authorguide#dataavailability).

In case you have no data that requires deposition in a public database, please state so in this section. Note that the Data Availability Section is restricted to new primary data that are part of this study.

7) For data quantification: please specify the name of the statistical test used to generate error bars and P values, the number (n) of independent experiments (specify technical or biological replicates) underlying each data point and the test used to calculate p-values in each figure legend. The figure legends should contain a basic description of n, P and the test applied. Graphs must include a description of the bars and the error bars (s.d., s.e.m.). Please provide exact p values.

8) Our journal encourages inclusion of *data citations in the reference list* to directly cite datasets that were re-used and obtained from public databases. Data citations in the article text are distinct from normal bibliographical citations and should directly link to the database records from which the data can be accessed. In the main text, data citations are formatted as

follows: "Data ref: Smith et al, 2001" or "Data ref: NCBI Sequence Read Archive PRJNA342805, 2017". In the Reference list, data citations must be labeled with "[DATASET]". A data reference must provide the database name, accession number/identifiers and a resolvable link to the landing page from which the data can be accessed at the end of the reference. Further instructions are available at .

9) We replaced Supplementary Information with Expanded View (EV) Figures and Tables that are collapsible/expandable online. A maximum of 5 EV Figures can be typeset. EV Figures should be cited as 'Figure EV1, Figure EV2" etc... in the text and their respective legends should be included in the main text after the legends of regular figures.
- For the figures that you do NOT wish to display as Expanded View figures, they should be bundled together with their legends in a single PDF file called *Appendix*, which should start with a short Table of Content. Appendix figures should be referred to in the main text as: "Appendix Figure S1, Appendix Figure S2" etc.
- Additional Tables/Datasets should be labeled and referred to as Table EV1, Dataset EV1, etc. Legends have to be provided in a separate tab in case of .xls files. Alternatively, the legend can be supplied as a separate text file (README) and zipped together with the Table/Dataset file.
See detailed instructions here:

10) The paper explained: EMBO Molecular Medicine articles are accompanied by a summary of the articles to emphasize the major findings in the paper and their medical implications for the non-specialist reader. Please provide a draft summary of your article highlighting
- the medical issue you are addressing,
- the results obtained and
- their clinical impact.
This may be edited to ensure that readers understand the significance and context of the research. Please refer to any of our published articles for an example.

11) For more information: There is space at the end of each article to list relevant web links for further consultation by our readers. Could you identify some relevant ones and provide such information as well? Some examples are patient associations, relevant databases, OMIM/proteins/genes links, author's websites, etc...

12) Author contributions: CRediT has replaced the traditional author contributions section because it offers a systematic machine readable author contributions format that allows for more effective research assessment. Please remove the Authors Contributions from the manuscript and use the free text boxes beneath each contributing author's name in our system to add specific details on the author's contribution. More information is available in our guide to authors.

13) Disclosure statement and competing interests: We updated our journal's competing interests policy in January 2022 and request authors to consider both actual and perceived competing interests. Please review the policy https://www.embopress.org/competing-interests and update your competing interests if necessary.

14) Every published paper now includes a 'Synopsis' to further enhance discoverability. Synopses are displayed on the journal webpage and are freely accessible to all readers. They include a short stand first (maximum of 300 characters, including space) as well as 2-5 one-sentences bullet points that summarizes the paper. Please write the bullet points to summarize the key NEW findings. They should be designed to be complementary to the abstract - i.e. not repeat the same text. We encourage inclusion of key acronyms and quantitative information (maximum of 30 words / bullet point). Please use the passive voice. Please attach these in a separate file or send them by email, we will incorporate them accordingly.

Please also suggest a striking image or visual abstract to illustrate your article as a PNG file 550 px wide x 300-600 px high.

15) As part of the EMBO Publications transparent editorial process initiative (see our Editorial at http://embomolmed.embopress.org/content/2/9/329), EMBO Molecular Medicine will publish online a Review Process File (RPF) to accompany accepted manuscripts.
In the event of acceptance, this file will be published in conjunction with your paper and will include the anonymous referee reports, your point-by-point response and all pertinent correspondence relating to the manuscript. Let us know whether you agree with the publication of the RPF and as here, if you want to remove or not any figures from it prior to publication.
Please note that the Authors checklist will be published at the end of the RPF.

EMBO Molecular Medicine has a "scooping protection" policy, whereby similar findings that are published by others during review or revision are not a criterion for rejection. Should you decide to submit a revised version, I do ask that you get in touch after three months if you have not completed it, to update us on the status.

I look forward to receiving your revised manuscript.

Yours sincerely,

Lise Roth

Lise Roth, PhD
Senior Editor
EMBO Molecular Medicine

Please note: When submitting your revision you will be prompted to enter your funding and payment information. This will allow Wiley to send you a quote for the article processing charge (APC) in case of acceptance. This quote takes into account any reduction or fee waivers that you may be eligible for. Authors do not need to pay any fees before their manuscript is accepted and transferred to the publisher.

EMBO Press participates in many Publish and Read agreements that allow authors to publish Open Access with reduced/no publication charges. Check your eligibility: https://authorservices.wiley.com/author-resources/Journal-Authors/open-access/affiliation-policies-payments/index.html

***** Reviewer's comments *****

Referee #1 (Comments on Novelty/Model System for Author):

If I interpret correctly the results presented the most frequent mutations found by the authors represent 15-25 cases (Fig. 1A) out of 138, 915 cases. That is, frequencies of 0.01 to 0,025 %. Is my interpretation correct? If so, it should be clearly stated. It is important when assessing the translational implications of this work.

Referee #1 (Remarks for Author):

In this manuscript, Lyer, S. R. et al functionally characterize mutant forms of the receptor tyrosine kinase ROS1 found in cancer. They find that some of these mutations, namely, D2113 to Q or G, over activate ROS1. Cells expressing these mutant forms of ROS1 are sensitive to specific tyrosine kinase inhibitors, such as crizotinib and lorlatinib. Thus, these mutations may be biomarkers of sensitivity to Ros inhibitors in the clinic.

While ROS1 gene rearrangements have been characterized, less is known about mutations. Thus, this manuscript addresses a timely issue. The authors showed that mutations in residue 2113 cause the overactivation of ROS1 and its likely transformation into an oncogene. The experiments shown are properly controlled and, in general, support the conclusions made by the authors.

To strengthen the manuscript, I would recommend the following:

1. Clearly state that the most frequent mutations found by the authors represent only 0.01 to 0.025% of the total cases (138,915) as per Fig. 1A. This is important when assessing the translational implications of this work.

2. Explain the discrepancy between the statement "We note that overexpression of wildtype ROS1 also increased colony formation potential compared to ROS1K1980E kinase dead or EV controls, albeit this was not significant" and the results shown in Fig. 2B where there is no difference between control cells expressing empty vector (EV), cells expressing wildtype ROS1, or cells expressing the kinase dead mutant.

3. Explain the choice of using the D2113N mutant for structural studies, as the D2113G mutant is comparably active in HEK293 cells (Fig. 1) and considerably more active in MCF10A cells (Fig. 2).

4. Further characterize the observation that minimal evidence of increased activity of either the canonical RAS-RAF-MEK-ERK or PI3K-AKT signaling pathways was observed in phosphoproteomic analysis as shown in Fig. 4, as this contrasts with current assumptions on other overactivated receptor tyrosine kinases, including ROS1 fusions. Analyzing the sensitivity of cells expressing the D2113 mutant or that of SLC-ROS1 to inhibitors of the MEK-ERK or PI3K-AKT pathways would be interesting.

Referee #2 (Comments on Novelty/Model System for Author):

Testing of mutant proteins in transformation assays and in vivo is adequate

Referee #2 (Remarks for Author):

Iyer and colleagues study the oncogenic capaity of missense mutations in the ROS1 oncogene and their sensitivity to FDA approved ROS1 inhibitors. This is relevant, because to date, there are no missense mutations in ROS1 for which the use of these ROS1 inhibitors is indicated. At present, only ROS1 fusions are eligible for treatment with ROS1 inhibitors.
Using public datasets, the authors identify 33 missense mutations in ROS1 that were each tested in functional assays and biochemical assays. Of these 33, only D2113N turned out to be highly active in auto-phosphorylation of ROS1 (indicative of kinase activation) as well as in transformation assays in NIH3T3 and MCF10A cells. Effects of this mutant on signaling was studied in HEK293 cells in comparison to wild type ROS1.Most relevant from a translational perspective was the finding that ROS1D2113N expressing NIH3T3 cells formed tumors in mice and were sensitive to FDA approved ROS1 inhibitors, thus suggesting that patients with particular ROS1 mutation might benefit from these drugs. This is relevant, as this mutation is also seen in patients with melanoma and oligodendroglioma.
Critique:
Overall, this is a very elegant and complete study. There is one major point that I feel needs to be addressed. The authors make a strong point that the mutant D2113N mutant differs in signaling downstream from the ROS1 fusion proteins, lacking signaling to MAPK and PI3K pathways. This is done in HEK293 cells without comparing D2113N to ROS fusions in these cells. There is additional data on signaling in NIH3T3 cells (Fig. 5G), but again, no comparison to ROS fusions. This is essential if the authors wish to claim differences in downstream signalling. In the present manuscript, this is simply not supported by data. Moreover, the data in Fig 5G show inhibition of pERK by crizotinib, which indicates that the D2113N oncogene does signal through the MAPK pathway.
Minor comment: the data of Figures 5A and E are easier to read is shown as growth curves.

Referee #3 (Comments on Novelty/Model System for Author):

The ROS1 oncogenic effects through fusion gene formations have been previously appreciated but little is known about the potential role of nonsense mutations found in the ROS1 kinase domain. Iyer et al identify several ROS1 mutations and characterized which ones may have oncogenic properties while also evaluating their sensitivity to ALK/ROS1 inhibitors. The authors isolated one mutation of special interest, ROS1 D2113N. They thoroughly studied the implications of this mutation to cancer using cell transformation assays, structural analyses, and pharmacological sensitivity to various inhibitors. For the most part the data are strong. However, the investigators never state the frequency of this mutation in different types of cancer, but based on other numbers it is probably very low. Without this information, it is not possible to judge the clinical impact of these studies. These data should be straight forward to access and include. Secondly, some of the major pathways that the authors claim to be differentially affected by the mutation in Fig 4 should be further validated using western blot analyses and include a fusion ROS1 as control. Lastly, effects on pSTAT3 and pERK in Fig 5 appear overstated.

Author Response to Referee Comments

We would like to thank the Referees for their time and constructive feedback. We have presented new data in existing figures and added two additional figures to address these questions. Where substantive data or interpretation related additions were made, the text color in the revised manuscript is blue; the specific page number and line numbers are also indicated in specific response below.

Specific Response:

Referee #1 (Comments on Novelty/Model System for Author):
If I interpret correctly the results presented the most frequent mutations found by the authors represent 15-25 cases (Fig. 1A) out of 138, 915 cases. That is, frequencies of 0.01 to 0,025 %. Is my interpretation correct? If so, it should be clearly stated. It is important when assessing the translational implications of this work.

Response: We thank the Referee for highlighting this point. We agree that given how rare these activating ROS1 mutations are, deliberate comment on this is warranted. To this end, we have added a table (**Figure EV1B**) that shows the frequency of these substitutions relative to total clinical cohort size interrogated. We also addressed this head-on in the Results section (**Page 18, Lines 459-460**) and in the Discussion section (**Page 32-33, Lines 823-829**).

Referee #1 (Remarks for Author):

In this manuscript, Lyer, S. R. et al functionally characterize mutant forms of the receptor tyrosine kinase ROS1 found in cancer. They find that some of these mutations, namely, D2113 to Q or G, over activate ROS1. Cells expressing these mutant forms of ROS1 are sensitive to specific tyrosine kinase inhibitors, such as crizotinib and lorlatinib. Thus, these mutations may be biomarkers of sensitivity to Ros inhibitors in the clinic.

While ROS1 gene rearrangements have been characterized, less is known about mutations. Thus, this manuscript addresses a timely issue. The authors showed that mutations in residue 2113 cause the overactivation of ROS1 and its likely transformation into an oncogene. The experiments shown are properly controlled and, in general, support the conclusions made by the authors.

To strengthen the manuscript, I would recommend the following:

1. Clearly state that the most frequent mutations found by the authors represent only 0.01 to 0.025% of the total cases (138,915) as per Fig. 1A. This is important when assessing the translational implications of this work.

Response: As stated above, we agree with the Referee that this is a salient point with relevance for translational implications. We have added a new table reflecting this in the **Figure EV1B (**and addressed this in the Results and the Discussion section as described above).

2. Explain the discrepancy between the statement "We note that overexpression of wildtype ROS1 also increased colony formation potential compared to ROS1K1980E kinase dead or EV controls, albeit this was not significant" and the results shown in Fig. 2B where there is no difference between control cells expressing empty vector (EV), cells expressing wildtype ROS1, or cells expressing the kinase dead mutant.

Response: The Referee is correct and we thank the Referee for pointing this out. This sentence was incorrectly stating data and were left in by accident. We have now edited this out in the revised manuscript.

3. Explain the choice of using the D2113N mutant for structural studies, as the D2113G mutant is comparably active in HEK293 cells (Fig. 1) and considerably more active in MCF10A cells (Fig. 2).

Response: We thank the Referee for the opportunity to clarify this. We initiated functional studies prior to the COVID-19 pandemic based on the cbioportal.org dataset available at that time, which largely included MSKCC, Metabric and TCGA cohorts. Subsequently, the AACR Genie dataset, which included cancer genomic sequencing data from larger and newer cohorts of patients, was integrated into and made accessible via the cbioportal.org portal. When we re-interrogated the datasets, we discovered additional D21113 substitutions, including D2113G and Y in the cancer genome. Given this, we repeated key wet laboratory studies including the D2113N and D2113G mutations, subsequently. However, the structural modelling studies had been completed prior to our re-interrogation of the newer cbioportal.org datasets. Our molecular dynamic simulation data suggests that D2113 in the wildtype restricts the conformational possibilities of A-loop in the

active kinase, whereas with the mutant, N2113, the loop flexibility in the active form is substantially increased. We hypothesize that this increase in the active-form conformational flexibility drives activation. An aspartate to asparagine mutation at the residue 2113 position represents the smallest possible structural change. In this context, change to a glycine 2113 represents one of the largest possible change in residue size. Given our data, we envision that the glycine 2113 mutation would even further increase the active form A-loop flexibility. Thus based on knowledge from D2113N mutation, we envision that similar dynamic changes in the A-loop of the DFG-in conformation will drive activation of the D2113G mutant kinase. We edited the Results section in the revised manuscript (**Page 23, Lines 576-581**) to comment on our prediction of the D2113G substitution impact on activation loop based on knowledge of D2113N. As shown, the biochemical (immunoblotting) and phenotypic (cell proliferation) data support that D2113G represents an even further increase in catalytic activation as compared to D2113N.

4. Further characterize the observation that minimal evidence of increased activity of either the canonical RAS-RAF-MEK-ERK or PI3K-AKT signaling pathways was observed in phosphoproteomic analysis as shown in Fig. 4, as this contrasts with current assumptions on other overactivated receptor tyrosine kinases, including ROS1 fusions. Analyzing the sensitivity of cells expressing the D2113 mutant or that of SLC-ROS1 to inhibitors of the MEK-ERK or PI3K-AKT pathways would be interesting.

Response: We thank the Referee for this excellent suggestion. We conducted real-time MCF10A cell proliferation studies using stable cell lines with or without treatment with the following inhibitors: SHP2 inhibitors (TNO155, RMC4550), MEK1/2 inhibitors (selumetinib and trametinib), JNK1/2/3 inhibitor (SP-600125), AKT inhibitor (afuresertib), MTOR inhibitor (everolimus), JAK2 inhibitor (ruxolitinib) and TGFBR1 inhibitor (galunisertib). These new results are shown in a new figure (**Figure 6** and **Expanded View 4**). Since this was a new dataset, the Results section was updated to discuss these findings on **Pages 28-29 (Lines 701 – 731)**.

Referee #2 (Comments on Novelty/Model System for Author):

Testing of mutant proteins in transformation assays and in vivo is adequate

Referee #2 (Remarks for Author):

Iyer and colleagues study the oncogenic capaity of missense mutations in the ROS1 oncogene and their sensitivity to FDA approved ROS1 inhibitors. This is relevant, because to date, there are no missense mutations in ROS1 for which the use of these ROS1 inhibitors is indicated. At present, only ROS1 fusions are eligible for treatment with ROS1 inhibitors.
Using public datasets, the authors identify 33 missense mutations in ROS1 that were each tested in functional assays and biochemical assays. Of these 33, only D2113N turned out to be highly active in auto-phosphorylation of ROS1 (indicative of kinase activation) as well as in transformation assays in NIH3T3 and MCF10A cells. Effects of this mutant on signaling was studied in HEK293 cells in comparison to wild type ROS1.Most relevant from a translational perspective was the finding that ROS1 D2113N expressing NIH3T3 cells formed tumors in mice and were sensitive to FDA approved ROS1 inhibitors, thus suggesting that patients with particular ROS1 mutation might benefit from these drugs. This is relevant, as this mutation is also seen in patients with melanoma and oligodendroglioma.

Critique:
Overall, this is a very elegant and complete study. There is one major point that I feel needs to be addressed. The authors make a strong point that the mutant D2113N mutant differs in signaling downstream from the ROS1 fusion proteins, lacking signaling to MAPK and PI3K pathways. This is done in HEK293 cells without comparing D2113N to ROS fusions in these cells. There is additional data on signaling in NIH3T3 cells (Fig. 5G), but again, no comparison to ROS fusions. This is essential if the authors wish to claim differences in downstream signalling. In the present manuscript, this is simply not supported by data. Moreover, the data in Fig 5G show inhibition of pERK by crizotinib, which indicates that the D2113N oncogene does signal through the MAPK pathway.

Response: We would like to thank the Referee for the positive feedback and important scientific suggestions to carefully delineate the downstream effector pathways invoked by activating variants in ROS1 full-length receptor as compared to oncogenic ROS1 fusions. We agree that additional studies were necessary to map the downstream effector signaling landscape of ROS1 receptor variants to ROS1 fusions. We have now performed new experiments with biological replicates, including the following:

(a) Immunoblotting of common signaling pathways activated downstream of ROS1^{D2113N}, ROS1^{D2113G} and ROS1 fusions (CD74-ROS1 and EZR-ROS1), including SHP2, ERK, AKT, mTOR, STAT3 and the potentially newer targets, JNK and/or cJun. These data were generated from stable HEK293A, NIH3T3 and MCF10A cell model systems.

(b) Real time cell proliferation as well as cell viability assays to interrogate the activity of inhibitors to SHP2 (TNO155, RMC4550), MEK1/2 (selumetinib, trametinib), JNK1/2/3 (SP600125), AKT (afuresertib) MTOR1 (everolimus), JAK1/2 (ruxolitinib), and TGFBR1 (galunisertib) in the MCF10A breast epithelial stable cell lines expressing wildtype ROS1, ROS1 D2113N, ROS1 D2113G and SLC-ROS1.

These data are now presented in new **Figures 5 and 6 and Figure EV4**. The data are reported in the Results section on **Pages 26 – 29, lines 647-731**.

(c) We would like to take the opportunity respond in regards to the complete inhibition of pERK1/2 by crizotinib as opposed to more modest or partial inhibition by entrectinib and lorlatinib in the P1-ROS1^{D2113N} cells (Fig. 5G of original submission, Fig. 7G of revision). As shown in the table below, in addition to targeting ROS1 with nanomolar potency, crizotinib, entrectinib and lorlatinib have additional kinase targets. Crizotinib has higher potency for MET than ROS1. In contrast, lorlatinib a newer inhibitor, is has higher potency and relatively higher selectivity for ROS1 than crizotinib. This is corroborated in the immunoblots showing auto-phosphorylation at ROS1 Y2274 blots where 10 nM lorlatinib invokes nearly complete inhibition of ROS1 autophosphorylation, whereas crizotinib <u>at the same concentration</u> only modestly inhibits ROS1. Based on the relatively selective pharmacological inhibition profile of lorlatinib compare to the polypharmacology of crizotinib, we conclude that crizotinib inhibits multiple RTKs that congruently contribute to the activation of MEK/ERK in NIH3T3 cells. In comparison, the contribution of ROS1 to ERK1/2 activation can be best appreciated in the lorlatinib-treated cells where partial inhibition of ERK1/2 is indeed observed. We have edited the manuscript to reflect these and other data that show that ROS1^{D2113N} does activate MEK/ERK pathway, albeit more modestly than ROS1 fusions and potentially conditioned by cell lineage (**Page 30**, **lines 766-776**).

	Crizotinib#	Entrectinib&	Lorlatinib*
Other Kinases Inhibited at with IC_{50} < 100 nM	MET (8-11 nM)<br>ALK (20-24 nM)<br>RON (80nm)<br>ROS1 (~25 nM)	TRKA, B or C (2-6 nM)<br>ROS1 (5 nM)<br>ALK (28nM)	ALK (< 1 nM)<br>ROS1 (< 1 nM)

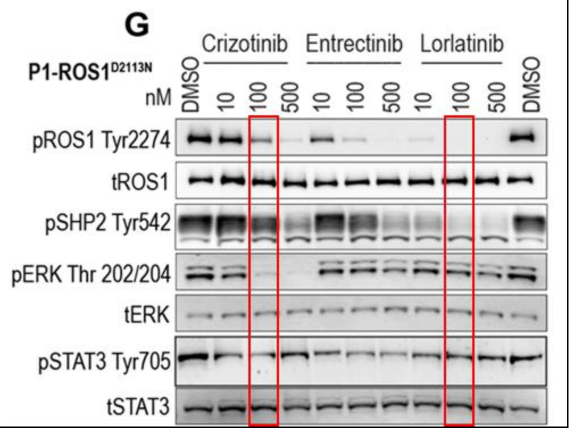

\# https://pubs.acs.org/doi/10.1021/jm2007613
& https://doi.org/10.1158/1535-7163.MCT-15-0758
* https://doi.org/10.1016/j.ccell.2015.05.010 & https://doi.org/10.1073/pnas.1420785112

Minor comment: the data of Figures 5A and E are easier to read is shown as growth curves.
<u>Response:</u> We agree and have now shown the tumor growth data in **Figure 7A, 7E** and **Figure EV5B**, as growth curves. (Note, Figure 5 from original submission is Figure 7 in the revised manuscript).

Referee #3 (Comments on Novelty/Model System for Author):

The ROS1 oncogenic effects through fusion gene formations have been previously appreciated but little is known about the potential role of nonsense mutations found in the ROS1 kinase domain. Iyer et al identify several ROS1 mutations and characterized which ones may have oncogenic properties while also evaluating their sensitivity to ALK/ROS1 inhibitors. The authors isolated one mutation of special interest, ROS1 D2113N. They thoroughly studied the implications of this mutation to cancer using cell transformation assays, structural analyses, and pharmacological sensitivity to various inhibitors. For the most part the data are strong. However, the investigators never state the frequency of this mutation in

different types of cancer, but based on other numbers it is probably very low. Without this information, it is not possible to judge the clinical impact of these studies. These data should be straight forward to access and include.

Response: We agree that given how rare these activating ROS1 mutations are, deliberate comment on this is warranted. To this end, we have added a table (**Figure EV1B**) that clearly shows the frequency of these substitutions relative to total clinical cohort size interrogated. We also addressed this in the Results section (**Page 18, Lines 459-460)** and in the Discussion section **(Page 32-33, Lines 823-829)**.

Secondly, some of the major pathways that the authors claim to be differentially affected by the mutation in Fig 4 should be further validated using western blot analyses and include a fusion ROS1 as control.

Response: We agree with the Referee on these points. To this end, we have now updated the manuscript the following new data sets:

- Western blotting from transiently transfected HEK293A cells with expression of ROS1 wildtype, ROS1^{D2113N}, ROS1^{D2113G} mutations compared to CD74-ROS1, EZR-ROS1 and SLC34A2-ROS1 fusions. These data are shown in **Figure 5A, B** of the revised manuscript. We observe that both the point mutations and the fusions activate SHP2, ERK1/2 and STAT3 phosphorylation, albeit the fusions have more robust activity. The Results section of the manuscript was updated to include these data (**Page 26, Lines 647 – 668**).

- Western blotting of lysates from HEK293A and NIH3T3 cells stably expressing ROS1^{D2113N} and CD74-ROS1 fusion after treatment with the ROS1 TKIs, lorlatinib and entrectinib. These data are now included in **Figure 5C, D** and findings described in the results section on **Pages 26-27, Lines 669 – 686**. These data broadly show that ROS1 TKI inhibits ERK1/2 more robustly in the ROS1 fusion cells compared to the ROS1 point mutation cells (although there is significant inhibition in the point mutant cells, albeit weaker than in ROS1 fusions).

- Western blotting of lysates from MCF10A cells stably expressing ROS1 wildtype (WT), ROS1 D2113N, ROS1 D2113G, CD74-ROS1, and EZR-ROS1 fusion. These data are shown in **Figure EV4**, and findings are reported in the Results section on **Page 27-28, Lines 687 – 700**.

Lastly, effects on pSTAT3 and pERK in Fig 5 appear overstated.

Response: Densitometry on replicate experimental data (**Figure 7G-J, Figure EV5D, E**) show that lorlatinib partially inhibits pERK1/2 and pSTAT3 in the P1-ROS1^{D2113N} tumor derived cell line. We have edited the Results section (**Page 30-31, Lines 764-778**).

19th Jul 2023

Dear Dr. Davare,

Thank you for the submission of your revised manuscript to EMBO Molecular Medicine, and please accept my apologies for the delay in getting back to you in this busy time of the year. We have now received the reports from the 3 referees who re-reviewed your manuscript. As you will see, they are overall supportive of publication pending minor revisions, and we will therefore be able to accept your manuscript once the following points will be addressed:

1/ Referees' comments: please address the remaining concerns/questions from referee #3 in writing.

2/ Main manuscript text:
- Thank you for addressing the data editors' queries. As they have not been able to review the EV figure legends, we would encourage to review those as well and align them to the formatting and presentation of the main figure legends regarding bars, error bars and nature of replicates.
- Please accept the previous changes, remove the blue text, and only keep in track changes mode any new modification.
- Please note that the email to Kevin Nusser bounced.
- The manuscript should respect the following order, please correct accordingly: abstract, introduction, results, discussion, materials & methods, data availability section, acknowledgments, disclosure statement and competing interests, references, main figure legends, expanded figure legends
- Materials and methods:
o Antibodies: please provide dilutions/concentrations used
o Please provide a statement on inclusion/exclusion criteria
- Data Availability: please simply indicate "This study includes no data deposited in external repositories."

3/ Figures and Appendix:
- Your manuscript contains error bars based on n=2 (Figure 5D). Please note that error bars and statistics cannot be used in this case.
- Please confirm that your manuscript does not include an Appendix file.

4/ Thank you for providing source data. Please merge the file "Source Data for questioned images" with the regular Source Data files. Source data should be submitted as 1 file per figure for the main figures, and 1 file for EV/Appendix figures

5/ Thank you for providing The paper explained. I included minor modifications, please let me know if you agree or amend as you see fit, and insert the text in the final manuscript file:

Problem:
The functional consequences of numerous ROS1 mutations discovered in the cancer genome are not characterized and remain as variants of unknown significance, hindering understanding of their clinical relevance and potential for targeted therapies. In this study, we focused on investigating the impact of somatic missense mutations residing in the kinase domain of ROS1.
Results:
The majority of studied ROS1 mutations have either neutral or deleterious impact on the catalytic function of the tyrosine kinase. However, a rare subset of mutations was activating. Among these, asparagine or glycine substitutions at the aspartate 2113 position in the activation loop of ROS1 robustly activated catalytic activity and promoted ROS1 TKI-sensitive oncogenic transformation and tumor formation in vivo.
Impact:
The identification of gain-of-function mutations in ROS1 and the corresponding therapeutic response to existing FDA-approved ROS1 TKIs offer potential personalized treatment strategies for ROS1-altered cancers.

6/ Thank you for providing "For more information". Would you agree to only keep:
1. https://www.cbioportal.org/
2. https://biit.cs.ut.ee/gprofiler/gost

7/ Synopsis:
- Thank you for providing a synopsis image. Please resize it as a jpeg/png/tiff fil 550 px wide x 200-400 px high, and make sure the text remains legible.
- I modified your synopsis text to fit our style and format, please let me know if you agree with the following:

The current understanding of ROS1 oncogenes is predominantly focused on ROS1 kinase fusion proteins, limiting clinical interventions with tyrosine kinase inhibitors (TKIs). Here, activating point mutations in ROS1 were investigated.
- D2113N and D2113G were identified as activating point mutations through functional screenings and AACR Genie database.

- ROS1 D2113N mutation was suggested to exert a major dynamic effect within the kinase domain by increasing the flexibility of the A-loop in the active (DFG-in) conformation.
- ROS1 D2113N and D2113G substitutions induced ROS1 TKI sensitive cell transformation in vitro, and ROS1 D2113N drove crizotinib and lorlatinib-sensitive tumor formation in vivo.
- D2113N and D2113G downstream signalling effects overlapped with those of ROS1 fusion oncogenes, but were not identical.
- Pharmacological inhibition of signalling effectors such as SHP2 and MEK1/2 robustly inhibited ROS1 mutation driven cell proliferation.

8/ As part of the EMBO Publications transparent editorial process initiative (see our Editorial at http://embomolmed.embopress.org/content/2/9/329), EMBO Molecular Medicine will publish online a Review Process File (RPF) to accompany accepted manuscripts.
This file will be published in conjunction with your paper and will include the anonymous referee reports, your point-by-point response and all pertinent correspondence relating to the manuscript. Let us know whether you agree with the publication of the RPF and as here, if you want to remove or not any figures from it prior to publication.
Please note that the Authors checklist will be published at the end of the RPF.

I look forward to receiving your revised manuscript.

Yours sincerely,

Lise Roth

Lise Roth, PhD
Senior Editor
EMBO Molecular Medicine

***** Reviewer's comments *****

Referee #1 (Comments on Novelty/Model System for Author):

The experiments presented fully support the conclusions made by the authors.

Referee #1 (Remarks for Author):

The authors have satisfactorily addressed the concerns on the previous version of the manuscript.

Referee #2 (Comments on Novelty/Model System for Author):

The ROS mutations are rather rare, which limits medical impact, but the data show that these rare mutations are actionable, so still relevant medically

Referee #2 (Remarks for Author):

The authors have now included comparison of mutant ROS to ROS translocations. The revisions are appropriate.

Referee #3 (Comments on Novelty/Model System for Author):

The authors have made significant revisions in response to reviewer comments. Overall, the technical approaches in this manuscript are strong, but unfortunately the potential clinical impact of the revised manuscript is still perceived to be low. Any conclusions about lineage based differences in signaling of the ROS1 fusions versus mutations is not appropriate given only 2-3 cell lines were compared. In Fig Expanded View 4, use of 250 nM for all the kinase inhibitors leads to inadequate inhibition by some. For example, the JNK inhibitor has to be used at uM concentrations.

Response to Referees:

Referee #1 (Comments on Novelty/Model System for Author):

The experiments presented fully support the conclusions made by the authors.

Referee #1 (Remarks for Author):

The authors have satisfactorily addressed the concerns on the previous version of the manuscript.

Referee #2 (Comments on Novelty/Model System for Author):

The ROS mutations are rather rare, which limits medical impact, but the data show that these rare mutations are actionable, so still relevant medically

Referee #2 (Remarks for Author):

The authors have now included comparison of mutant ROS to ROS translocations. The revisions are appropriate.

Response to Referees #1 and 2: Thank you for reviewing the revised manuscript. We appreciated your original comments that prompted additional experiments to ultimately enhance the quality and depth of the manuscript.

Referee #3 (Comments on Novelty/Model System for Author):

The authors have made significant revisions in response to reviewer comments. Overall, the technical approaches in this manuscript are strong, but unfortunately the potential clinical impact of the revised manuscript is still perceived to be low. Any conclusions about lineage based differences in signaling of the ROS1 fusions versus mutations is not appropriate given only 2-3 cell lines were compared. In Fig Expanded View 4, use of 250 nM for all the kinase inhibitors leads to inadequate inhibition by some. For example, the JNK inhibitor has to be used at uM concentrations.

Response: Thank you for the opportunity to address these concerns.

(a) In the manuscript, we acknowledge that the incidence of oncogenic ROS1 point mutations is relatively low. However, in the era of post-genomic precision medicine and repurposing FDA-approved agents for personalized treatments (n = 1) based on biomarkers of response, we believe that even if a single patient can benefit from this treatment, there is meaningful clinical impact. Moreover, our study represents the first investigation, to the best of our knowledge, which sheds light on ROS1's pathogenic potential through receptor mutations, not just fusions. We respectfully propose that when examining a genomic query with only a snapshot in time, the observed incidence may appear low. Nonetheless, we respectfully request that you consider the significance of these data towards the potential benefits for individual patients in the context of precision medicine.

(b) We appreciate this concern in regards to making conclusions regarding signaling pathway activation given the limited model cell types used in our studies. Given this, we have edited the Results section to simply state the data as found in MCF10A cells and removed any references to comparison between cell lines or cell lineages. Please refer to Pages 15-16, Lines 378-393 in the revised manuscript (PDF document with tracked changes).

(c) We selected the 250 nM concentration for our experiment due to the prohibitive scale of performing dose-response testing on all agents. For most of the inhibitors used, we referred to reported cell-based or in vitro (recombinant protein) IC50s and opted for concentrations equivalent to, or 1.5-10 times higher than those required to achieve full effects (e.g., ROS1, MEK1/2, and MTOR/AKT inhibitors). We hoped that this approach would help us avoid off-target effects associated with micromolar concentrations. For instance, the Ki of SP600125 for recombinant JNK1/2/3 is 190 nM, thus we chose 250 nM. However, we acknowledge that in the case of SP-600125, our strategy resulted in using this inhibitor at lower levels than in previously reported studies, as correctly pointed out by the Referee.

To address this concern, we have added a caveat in the Results section (Page 17, Lines 425-426) to acknowledge the use of SP-600125 at a lower concentration than in previous studies. Notably, it is important to consider that at a 10-micromolar concentration, SP600125 inhibits 58 other kinases with 80% or higher inhibitory efficiency, as observed in recombinant kinase studies. We refer to the SP600125 profiling data from the Library of Integrated Network-based Cellular Signatures (LINCS) database. Despite the lower concentration used, we believe that our finding, wherein an effect was elicited with SP600125 at a concentration as low as 250 nM, is surprising and noteworthy. Therefore, we believe respectfully suggest that we should report this observation in the context of our study.

Considering that the primary focus of this paper revolves around the discovery of new oncogenic mutations in ROS1 and the evaluation of ROS1 TKI activity in this context, we sincerely hope that this relatively minor concern does not diminish the overall impact of our findings.

nall Molecule Nar	Protein Name	Activity (% of Control)	Assay compound conc	Conc unit	% Inhibition
SP600125	JNK1	0	10	uM	100
SP600125	MP2K4	0	10	uM	100
SP600125	MKNK2	0	10	uM	100
SP600125	RPS6KA4(Kin.Dor	0	10	uM	100
SP600125	IKKA	0.1	10	uM	99.9
SP600125	MYLK4	0.3	10	uM	99.7
SP600125	BIKE	0.6	10	uM	99.4
SP600125	MP2K3	0.7	10	uM	99.3
SP600125	MYLK	0.7	10	uM	99.3
SP600125	PLK4	0.7	10	uM	99.3
SP600125	ANKK1	1	10	uM	99
SP600125	TTK	1	10	uM	99
SP600125	GAK	1.2	10	uM	98.8
SP600125	DAPK1	1.4	10	uM	98.6
SP600125	STK16	1.4	10	uM	98.6
SP600125	MST3	1.8	10	uM	98.2
SP600125	MINK	1.9	10	uM	98.1
SP600125	RIPK4	2.2	10	uM	97.8
SP600125	MST4	2.4	10	uM	97.6
SP600125	AAK1	2.6	10	uM	97.4
SP600125	RIOK3	2.6	10	uM	97.4
SP600125	RPS6KA6(Kin.Dor	3	10	uM	97
SP600125	DAPK3	3.2	10	uM	96.8
SP600125	RPS6KA2(Kin.Dor	3.2	10	uM	96.8
SP600125	CLK2	3.4	10	uM	96.6
SP600125	RPS6KA4(Kin.Dor	3.4	10	uM	96.6
SP600125	PHKG2	3.6	10	uM	96.4
SP600125	RPS6KA1(Kin.Dor	3.8	10	uM	96.2
SP600125	DAPK2	4.1	10	uM	95.9
SP600125	ULK1	4.2	10	uM	95.8
SP600125	BMPR2	5	10	uM	95
SP600125	CDK16	5.2	10	uM	94.8
SP600125	SNARK	5.8	10	uM	94.2
SP600125	MP2K2	6	10	uM	94
SP600125	ULK2	6.4	10	uM	93.6
SP600125	MK09	6.6	10	uM	93.4
SP600125	JNK3	7.2	10	uM	92.8
SP600125	MP2K1	7.2	10	uM	92.8
SP600125	CAMKK2	10	10	uM	90
SP600125	RIOK1	10	10	uM	90
SP600125	ULK3	11	10	uM	89
SP600125	BMPR1B	12	10	uM	88
SP600125	IKKE	12	10	uM	88
SP600125	MP2K6	12	10	uM	88
SP600125	RPS6KA5(Kin.Dor	12	10	uM	88
SP600125	CAMK1D	13	10	uM	87
SP600125	CSNK1D	13	10	uM	87
SP600125	IKKB	13	10	uM	87
SP600125	PIM3	14	10	uM	86
SP600125	TAOK1	14	10	uM	86
SP600125	CAMK1	15	10	uM	85
SP600125	NEK1	15	10	uM	85
SP600125	TNK1	15	10	uM	85
SP600125	CAMK1G	17	10	uM	83
SP600125	TNIK	18	10	uM	82
SP600125	KKCC1	20	10	uM	80
SP600125	DRAK1	20	10	uM	80
SP600125	PRKR	20	10	uM	80

These data are a subset from the LINCS KINOMEScan profiling: https://lincs.hms.harvard.edu/kinomescan/

26th Jul 2023

Dear Dr. Davare,

Thank you for sending the revised files. I am pleased to inform you that your manuscript is now accepted for publication in EMBO Molecular Medicine!

Before we can send it to our publisher, we would need a resized synopsis (the current one is still too big, and when I tried to resize it, the text became illegible). Please send us a resized file (550 x 300-600 px) via email, and we will upload it in the submission system.

We would like to remind you that as part of the EMBO Publications transparent editorial process initiative, EMBO Molecular Medicine will publish a Review Process File online to accompany accepted manuscripts. If you do NOT want the file to be published or would like to exclude figures, please immediately inform the editorial office via e-mail.

Please read below for additional IMPORTANT information regarding your article, its publication and the production process.

Congratulations on your interesting work!

With kind regards,

Lise Roth

Lise Roth, Ph.D
Senior Editor
EMBO Molecular Medicine

Follow us on Twitter @EmboMolMed
Sign up for eTOCs at embopress.org/alertsfeeds

*** *** *** IMPORTANT INFORMATION *** *** ***

SPEED OF PUBLICATION
The journal aims for rapid publication of papers, using using the advance online publication "Early View" to expedite the process: A properly copy-edited and formatted version will be published as "Early View" after the proofs have been corrected. Please help the Editors and publisher avoid delays by providing e-mail address(es), telephone and fax numbers at which author(s) can be contacted.

Should you be planning a Press Release on your article, please get in contact with embomolmed@wiley.com as early as possible, in order to coordinate publication and release dates.

LICENSE AND PAYMENT:

All articles published in EMBO Molecular Medicine are fully open access: immediately and freely available to read, download and share.

EMBO Molecular Medicine charges an article processing charge (APC) to cover the publication costs. You, as the corresponding author for this manuscript, should have already received a quote with the article processing fee separately. Please let us know in case this quote has not been received.

Once your article is at Wiley for editorial production you will receive an email from Wiley's Author Services system, which will ask you to log in and will present you with the publication license form for completion. Within the same system the publication fee can be paid by credit card, an invoice, pro forma invoice or purchase order can be requested.

Payment of the publication charge and the signed Open Access Agreement form must be received before the article can be published online.

PROOFS

You will receive the proofs by e-mail approximately 2 weeks after all relevant files have been sent o our Production Office. Please return them within 48 hours and if there should be any problems, please contact the production office at embopressproduction@wiley.com.

Please inform us if there is likely to be any difficulty in reaching you at the above address at that time. Failure to meet our deadlines may result in a delay of publication.

All further communications concerning your paper proofs should quote reference number EMM-2022-17367-V3 and be directed to the production office at embopressproduction@wiley.com.

EMBO Press Author Checklist

Corresponding Author Name: Monika Davare	
Journal Submitted to: EMBO Molecular Medicine	
Manuscript Number: EMM-2022-17367	

USEFUL LINKS FOR COMPLETING THIS FORM
The EMBO Journal - Author Guidelines
EMBO Reports - Author Guidelines
Molecular Systems Biology - Author Guidelines
EMBO Molecular Medicine - Author Guidelines

Reporting Checklist for Life Science Articles (updated January
This checklist is adapted from Materials Design Analysis Reporting (MDAR) Checklist for Authors. MDAR establishes a minimum set of requirements in transparent reporting in the life sciences (see Statement of Task: 10.31222/osf.io/9sm4x). Please follow the journal's guidelines in preparing your
Please note that a copy of this checklist will be published alongside your article.

Abridged guidelines for figures
1. Data
The data shown in figures should satisfy the following conditions:
- the data were obtained and processed according to the field's best practice and are presented to reflect the results of the experiments in an accurate and unbiased manner.
- ideally, figure panels should include only measurements that are directly comparable to each other and obtained with the same assay.
- plots include clearly labeled error bars for independent experiments and sample sizes. Unless justified, error bars should not be shown for technical
- if n<5, the individual data points from each experiment should be plotted. Any statistical test employed should be justified.
- Source Data should be included to report the data underlying figures according to the guidelines set out in the authorship guidelines on Data

2. Captions
Each figure caption should contain the following information, for each panel where they are relevant:
- a specification of the experimental system investigated (eg cell line, species name).
- the assay(s) and method(s) used to carry out the reported observations and measurements.
- an explicit mention of the biological and chemical entity(ies) that are being measured.
- an explicit mention of the biological and chemical entity(ies) that are altered/varied/perturbed in a controlled manner.
- the exact sample size (n) for each experimental group/condition, given as a number, not a range;
- a description of the sample collection allowing the reader to understand whether the samples represent technical or biological replicates (including how many animals, litters, cultures, etc.).
- a statement of how many times the experiment shown was independently replicated in the laboratory.
- definitions of statistical methods and measures:
  - common tests, such as t-test (please specify whether paired vs. unpaired), simple χ2 tests, Wilcoxon and Mann-Whitney tests, can be unambiguously identified by name only, but more complex techniques should be described in the methods section;
  - are tests one-sided or two-sided?
  - are there adjustments for multiple comparisons?
  - exact statistical test results, e.g., P values = x but not P values < x;
  - definition of 'center values' as median or average;
  - definition of error bars as s.d. or s.e.m.

Please complete ALL of the questions below.
Select "Not Applicable" only when the requested information is not relevant for your study.

Materials

Newly Created Materials	Information included in the manuscript?	In which section is the information available? (Reagents and Tools Table, Materials and Methods, Figures, Data Availability Section)
New materials and reagents need to be available; do any restrictions apply?	Yes	Data Availability Section

Antibodies	Information included in the manuscript?	In which section is the information available? (Reagents and Tools Table, Materials and Methods, Figures, Data Availability Section)
For **antibodies** provide the following information:<br>- Commercial antibodies: RRID (if possible) or supplier name, catalogue number and or/clone number<br>- Non-commercial: RRID or citation	Yes	Catalog Number, Supplier Name and RRID is provided in the Materials and Methods Section

DNA and RNA sequences	Information included in the manuscript?	In which section is the information available? (Reagents and Tools Table, Materials and Methods, Figures, Data Availability Section)
**Short novel DNA or RNA including primers, probes**: provide the sequences.	Not Applicable	

Cell materials	Information included in the manuscript?	In which section is the information available? (Reagents and Tools Table, Materials and Methods, Figures, Data Availability Section)
**Cell lines:** Provide species information, strain. Provide accession number in repository **OR** supplier name, catalog number, clone number, and/**OR** RRID.	Yes	Information provided in Materials and Methods Section
**Primary cultures:** Provide species, strain, sex of origin, genetic modification status.	Not Applicable	
Report if the cell lines were recently **authenticated** (e.g., by STR profiling) and tested for mycoplasma contamination.	Yes	Information provided in Materials and Methods Section

Experimental animals	Information included in the manuscript?	In which section is the information available? (Reagents and Tools Table, Materials and Methods, Figures, Data Availability Section)
**Laboratory animals or Model organisms:** Provide species, strain, sex, age, genetic modification status. Provide accession number in repository **OR** supplier name, catalog number, clone number, **OR** RRID.	Yes	Information provided in Materials and Methods Section
**Animal observed in or captured from the field:** Provide species, sex, and age where possible**.**	Not Applicable	
Please detail **housing and husbandry conditions**.	Yes	Information provided in Materials and Methods Section

Plants and microbes	Information included in the manuscript?	In which section is the information available? (Reagents and Tools Table, Materials and Methods, Figures, Data Availability Section)
**Plants:** provide species and strain, ecotype and cultivar where relevant, unique accession number if available, and source (including location for collected wild specimens).	Not Applicable	
**Microbes:** provide species and strain, unique accession number if available, and source.	Not Applicable	

Human research participants	Information included in the manuscript?	In which section is the information available? (Reagents and Tools Table, Materials and Methods, Figures, Data Availability Section)
If collected and within the bounds of privacy constraints report on age, sex and gender or ethnicity for all study participants.	Not Applicable	

Core facilities	Information included in the manuscript?	In which section is the information available? (Reagents and Tools Table, Materials and Methods, Figures, Data Availability Section)
If your work benefited from core facilities, was their service mentioned in the acknowledgments section?	Yes	In the Acknowledgements Section.

Design

Study protocol	Information included in the manuscript?	In which section is the information available? (Reagents and Tools Table, Materials and Methods, Figures, Data Availability Section)
If study protocol has been **pre-registered, provide DOI in the manuscript**. For clinical trials, provide the trial registration number **OR** cite DOI.	Not Applicable	
Report the **clinical trial registration number** (at ClinicalTrials.gov or equivalent), where applicable.	Not Applicable	

Laboratory protocol	Information included in the manuscript?	In which section is the information available? (Reagents and Tools Table, Materials and Methods, Figures, Data Availability Section)
Provide DOI OR other citation details if **external detailed step-by-step protocols** are available.	Not Applicable	

Experimental study design and statistics	Information included in the manuscript?	In which section is the information available? (Reagents and Tools Table, Materials and Methods, Figures, Data Availability Section)
Include a statement about **sample size** estimate even if no statistical methods were used.	Yes	In Figure Legends
Were any steps taken to minimize the effects of subjective bias when allocating animals/samples to treatment (e.g. **randomization procedure**)? If yes, have they been described?	Yes	Information provided in Materials and Methods Section
Include a statement about **blinding** even if no blinding was done.	Yes	Information provided in Materials and Methods Section
Describe **inclusion/exclusion criteria** if samples or animals were excluded from the analysis. Were the criteria pre-established?<br><br>If sample or data points were omitted from analysis, report if this was due to attrition or intentional exclusion and provide justification.	Not Applicable	
For every figure, are **statistical tests** justified as appropriate? Do the data meet the assumptions of the tests (e.g., normal distribution)? Describe any methods used to assess it. Is there an estimate of variation within each group of data? Is the variance similar between the groups that are being statistically compared?	Yes	Statistical methods are described in Figure Legends and Material and Methods.

Sample definition and in-laboratory replication	Information included in the manuscript?	In which section is the information available? (Reagents and Tools Table, Materials and Methods, Figures, Data Availability Section)
In the figure legends: state number of times the experiment was **replicated** in laboratory.	Yes	Figure Legend.
In the figure legends: define whether data describe **technical or biological replicates**.	Yes	Figure Legend.

Ethics

Ethics	Information included in the manuscript?	In which section is the information available? (Reagents and Tools Table, Materials and Methods, Figures, Data Availability Section)
Studies involving **human participants**: State details of **authority granting ethics approval** (IRB or equivalent committee(s), provide reference number for approval.	Not Applicable	
Studies involving **human participants**: Include a statement confirming that **informed consent** was obtained from all subjects and that the experiments conformed to the principles set out in the WMA Declaration of Helsinki and the Department of Health and Human Services Belmont Report.	Not Applicable	
Studies involving **human participants: For** publication of **patient photos**, include a statement confirming that consent to publish was obtained.	Not Applicable	
Studies involving experimental **animals**: State details of **authority granting ethics approval** (IRB or equivalent committee(s), provide reference number for approval. Include a statement of compliance with ethical regulations.	Yes	Information provided in Materials and Methods Section
Studies involving **specimen and field samples:** State if relevant **permits** obtained, provide details of authority approving study; if none were required, explain why.	Not Applicable	

Dual Use Research of Concern (DURC)	Information included in the manuscript?	In which section is the information available? (Reagents and Tools Table, Materials and Methods, Figures, Data Availability Section)
Could your study fall under dual use research restrictions? Please check biosecurity documents and list of **select agents and toxins** (CDC): https://www.selectagents.gov/sat/list.htm	Not Applicable	
If you used a select agent, is the security level of the lab appropriate and reported in the manuscript?	Not Applicable	
If a study is subject to dual use research of concern regulations, is the name of the **authority granting approval and reference number** for the regulatory approval provided in the manuscript?	Not Applicable	

Reporting

The MDAR framework recommends adoption of discipline-specific guidelines, established and endorsed through community initiatives. Journals have their own policy about requiring specific guidelines and recommendations to complement MDAR.

Adherence to community standards	Information included in the manuscript?	In which section is the information available? (Reagents and Tools Table, Materials and Methods, Figures, Data Availability Section)
State if relevant guidelines or checklists (e.g., **ICMJE, MIBBI, ARRIVE, PRISMA**) have been followed or provided.	Not Applicable	
For **tumor marker prognostic studies,** we recommend that you follow the **REMARK** reporting guidelines (see link list at top right). See author guidelines, under 'Reporting Guidelines'. Please confirm you have followed these guidelines.	Not Applicable	
For **phase II and III randomized controlled trials**, please refer to the **CONSORT** flow diagram (see link list at top right) and submit the CONSORT checklist (see link list at top right) with your submission. See author guidelines, under 'Reporting Guidelines'. Please confirm you have submitted this list.	Not Applicable	

Data Availability

Data availability	Information included in the manuscript?	In which section is the information available? (Reagents and Tools Table, Materials and Methods, Figures, Data Availability Section)
Have **primary datasets** been deposited according to the journal's guidelines (see 'Data Deposition' section) and the respective accession numbers provided in the Data Availability Section?	Not Applicable	
Were **human clinical and genomic datasets** deposited in a public access-controlled repository in accordance to ethical obligations to the patients and to the applicable consent agreement?	Not Applicable	
Are **computational models** that are central and integral to a study available without restrictions in a machine-readable form? Were the relevant accession numbers or links provided?	Not Applicable	
If publicly available data were reused, provide the respective **data citations in the reference list**.	Yes	Citation for Databases in References and URLs in "For more information"
